# Sulfide and Oxide Inorganic Solid Electrolytes for All-Solid-State Li Batteries: A Review

**DOI:** 10.3390/nano10081606

**Published:** 2020-08-15

**Authors:** Mogalahalli V. Reddy, Christian M. Julien, Alain Mauger, Karim Zaghib

**Affiliations:** 1 Centre of Excellence in Transportation Electrification and Energy Storage (CETEES), Institute of Research Hydro-Québec, 1806, Lionel-Boulet Blvd., Varennes, QC J3X 1S1, Canada; MogalahalliVenkatesh.VenkatashamyReddy@hydroquebec.com; 2 Institut de Minéralogie, de Physique des Matériaux et de Cosmochimie (IMPMC), Sorbonne Université, UMR-CNRS 7590, 4 place Jussieu, 75252 Paris, France; alain.mauger@sorbonne-universite.fr; 3 Department of Mining and Materials Engineering, McGill University, Wong Building, 3610 University Street, Montreal, QC H3A OC5, Canada

**Keywords:** electrolytes, solid state, nanomaterials, sulfides, oxides, all-solid-state batteries, energy storage, composites

## Abstract

Energy storage materials are finding increasing applications in our daily lives, for devices such as mobile phones and electric vehicles. Current commercial batteries use flammable liquid electrolytes, which are unsafe, toxic, and environmentally unfriendly with low chemical stability. Recently, solid electrolytes have been extensively studied as alternative electrolytes to address these shortcomings. Herein, we report the early history, synthesis and characterization, mechanical properties, and Li^+^ ion transport mechanisms of inorganic sulfide and oxide electrolytes. Furthermore, we highlight the importance of the fabrication technology and experimental conditions, such as the effects of pressure and operating parameters, on the electrochemical performance of all-solid-state Li batteries. In particular, we emphasize promising electrolyte systems based on sulfides and argyrodites, such as LiPS_5_Cl and β-Li_3_PS_4_, oxide electrolytes, bare and doped Li_7_La_3_Zr_2_O_12_ garnet, NASICON-type structures, and perovskite electrolyte materials. Moreover, we discuss the present and future challenges that all-solid-state batteries face for large-scale industrial applications.

## 1. Introduction

**Figure 1:**
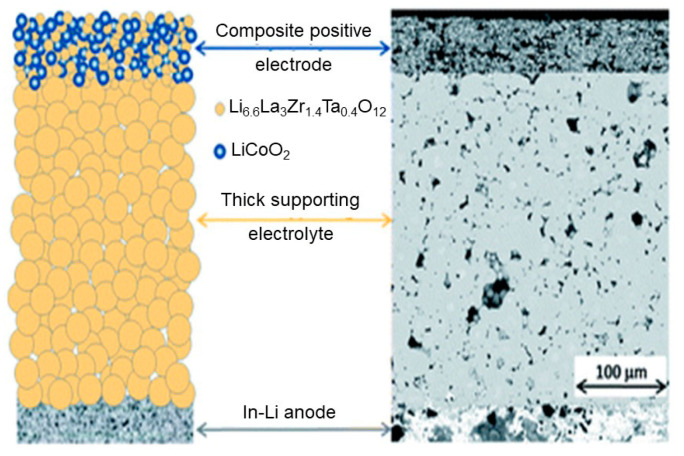
Schematic diagram of the fabricated electrolyte for all-solid-state Li batteries and its cross-sectional scanning electron micrograph. Reproduced with permission from [13]. Copyright 2018 Royal Society of Chemistry.

Inorganic oxide and sulfide materials have recently been studied as solid electrolytes for all-solid-state batteries (ASSBs) owing to their high safety profile, wide temperature window, and better mechanical properties than those of liquid electrolytes. Solid-state electrolytes (SSEs) can be widely used for solid-state Li batteries [1,2], sensors [3,4], fuel cells [1], Li-air [1,5,6], and Li-S [7] batteries. Although solid-state electrolytes can be used for all these different applications, we focused mainly on electrolytes for all-solid-state Li batteries. Recently, Reddy et al. [8] summarized the early history of Li batteries. In brief, a Li battery consists of a cathode (positive electrode), an electrolyte (Li ionic conductor), a separator, and an anode (negative electrode). The cathode material consists of either LiCoO_2_ (LCO), Li(Ni*_x_*Mn*_y_*Co_z_)O_2_ (NMC), LiFePO_4_ (LFP), or LiMn_2_O_4_ (LMO), and in some cases intercalated binary oxides, whereas Li metal, Li-In alloys, graphite, Li_4_Ti_5_O_12_ (LTO), or Si, Sn-Co-C mixed composites are used as anode materials [2]. In addition, Li batteries use liquid [9], gel polymer [10,11,12], or combinations of polymer and solid electrolytes. The electrode preparation techniques for all-solid-state lithium batteries (ASSLBs) differ from those of commercial Li batteries. Furthermore, the fabrication technologies of oxide and sulfide electrolyte-based ASSBs are different. For example, carbon is used as a conductive additive during the fabrication of sulfide electrolytes but not for the fabrication of oxide electrolytes. Moreover, depending on the mechanical properties of sulfide electrolytes, a suitable stack pressure is required for the assembly of ASSBs. Oxide solid electrolytes require high-temperature (>700 °C) sintering to improve the particle-particle contact between electrode and electrolyte. The general schematic diagram of ASSBs is presented in Figure 1. The ideal electrolyte materials for ASSBs should feature the following important properties: (i) High ionic conductivity of 10^−3^ S cm^−1^ at room temperature, (ii) low electronic conductivity of <10^−8^ S cm^−1^, which prevents their self-discharge, (iii) wide electrochemical potential window, (iv) good chemical stability over the operating temperature range and toward the electrodes, (v) transference number of approximately 1, (vi) matching thermal expansion coefficients with the cathode materials, (vii) good chemical stability; no crystal structure phase transformation should occur for the electrode active materials up to/near their sintering temperatures, (viii) their sintering temperature should match that of the electrode active materials, and (xv) low toxicity and cost effective [13].

Many researchers have investigated new solid electrolytes to replace flammable liquid electrolytes or improve the performance of existing solid electrolytes and elucidate their fundamental properties and technological developments. Huggins (1977) [14], Weppner (1981) [15], Kulkarni et al. (1984) [16], Minami (1985) [17], Pardel and Ribes (1989) [18], Adachi et al. (1996) [19], Owens (2000) [20], Thangadurai and Weppner (2002) [21], Knauth (2009) [22], and Fergus (2010) [23] published reviews on solid electrolytes. The journal *Solid State Ionics* devoted to these materials was created in 1980. This has been considered a hot research topic worldwide and has generated many publications. To highlight the advances on solid electrolyte fundamentals and electrode/electrolyte interface, analysis and its applications have been reviewed by many workers. We highlight a few important reviews in the following section.

The large number of reviews on solid electrolytes published during the last five years was attributed to the increasing interest in the use of solid electrolytes for electric vehicles (EVs) applications owing to their safety. Tatsumisago et al. [24] and Sakuda et al. [25] published important reviews on sulfide electrolytes, while Thangadurai et al. [26,27] reviewed garnet electrolytes. Furthermore, the fundamentals of ASSBs were reviewed by several authors [28,29,30,31,32,33,34,35]. The number of reviews on various aspects of electrolytes, cathodes, mechanical properties, and interface engineering has grown exponentially since 2018 [36,37,38,39,40,41,42,43,44,45,46,47,48,49,50,51,52,53,54,55,56,57,58,59,60,61,62,63,64,65,66,67,68,69,70,71,72,73,74,75,76,77,78,79,80,81,82,83,84,85,86,87,88,89,90,91,92,93,94,95,96,97,98,99,100,101,102,103,104,105,106,107,108,109,110,111,112,113,114,115]. For example, Famprikis et al. [51] and Zhang et al. [116] reported on the fundamentals of electrolytes and Oudenhoven et al. [117], Julien and Mauger [60], and Rambabu et al. [118] reviewed the technology of solid-state microbatteries. Moreover, in situ and ex situ techniques were explored for elucidating the solid electrode/electrolyte interfaces [40,67,80,98,119,120,121,122,123] and computational methods were reviewed by Xiao et al. [94] for understanding the conduction mechanisms in both oxide and sulfide electrolytes.

Herein, we report the brief history of each electrolyte system, summarize the recent advances in solid electrolytes (oxides vs. sulfides) for ASSB applications, highlight the importance of the cell fabrication technology and process parameters on the electrochemical storage performance, mechanical properties, and interfacial mechanisms of the cells, and examine the challenges of the large-scale fabrication of ASSBs. Furthermore, we summarize the important recent reports on electrolyte materials. Owing to the vast literature on this topic, we were unable to include and highlight all the pertinent publications in this review; however, some of the older publications are referenced in the most recent reviews.

## 2. Ionic Conduction in the Solid State

### 2.1. Ionic Conduction

In an idealized crystalline structure, there is little space for an ion to diffuse. The available space is only limited for vibration around its equilibrium position. In real systems, the degree of disorder that generates point defects (Schottky or Frenkel defects) results in vacant sites in the crystal and any ion in the immediate vicinity can jump from lattice site to lattice site. Ionic conduction is provoked by the motion of some positively (or negatively) charged ions, which “hop” under the influence of an electric field *F*. This ionic conductivity σ_i_ is expressed by:

σ_i_ = *n*_i_*e µ*_i_, (1)

where *n*_i_ is the number of ions per unit volume, *µ*_i_ the mobility of ions and *e* their charge. To move through the crystalline network, ions must have sufficient energy to pass an energy barrier *E*_a_. Thus, *n*_i_ in Equation (1) depends on the defect concentration in the crystal. So, in ionic frameworks, the movement of ions is in fact the movement of vacancies. Regarding the defect concentration, a useful classification of solid-state ionic conductors was proposed by Rice and Roth [124] as follows:

- Type I: Ionic solids with low concentration of defects ~10^18^ cm^−3^ at room temperature. They include compounds with poor ionic conduction (NaCl, LiCl, etc.).
- Type II: Ionic solids with high concentration of defects ~10^20^ cm^−3^ at room temperature. They are good ionic conductors (“fast-ionic conductors”, FICs), which belong to the class of materials of “vacancy migration”.
- Type III: Best FICs, which have a “molten” sub-lattice or “liquid like” structure of the mobile ions whose concentration is typically 10^22^ cm^−3^. The conduction mechanism in such FICs is mostly “interstitial”.

In practice, for a useful solid electrolyte, the electronic conductivity σ_e_ is undesirable and the transference number *t*_i_ is defined as the ratio of the ionic conductivity to the total conductivity

*t*_i_ = σ_i_ /(σ_e_ + σ_i_) ≈ 1. (2)

**Figure 2:**
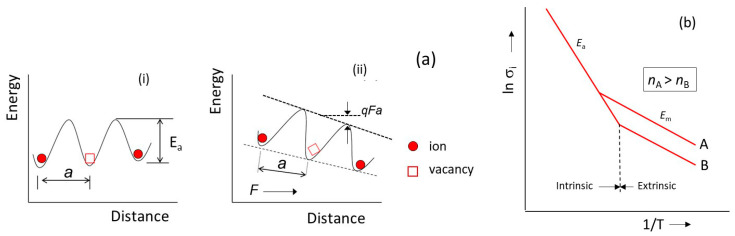
(**a**) Scheme of the potential barrier, which an ion has to overcome to exchange its site with a vacancy: (ii) Without external electric field and (ii) with external electric field. (**b**) Arrhenius plot of the ionic conductivity (ln σ_i_ vs. 1/*T*). The intrinsic and extrinsic regions are characterized by different *E*_a_ values.

In the one-dimensional (1D) model, the probability per unit time (*P*) for a vacancy to move to the next position in the absence of electric field is given by:

(3) $$P=\nu_{0}\exp\left(-\frac{E_{a}}{k_{B}T}\right),$$

where ν_0_ is the attempt frequency, *T* is the absolute temperature, *k*_B_ is the Boltzmann constant, and *E*_a_ is the potential barrier height or activation energy. Under an electric field, the barrier height is changed by the quantity *eFa* (see Figure 2a), where *a* is the lattice constant. The probabilities for the vacancy to move in the direction of the field (*P*′) and in the opposite direction to the field (*P*″) can be written as:

(4) $$P^{\prime }=\nu_{0}\exp\left(-\frac{E_{a}+\frac{1}{2}eFa}{k_{B}T}\right),$$

(5) $$P^{\prime \prime }=\nu_{0}\exp\left(-\frac{E_{a}-\frac{1}{2}eFa}{k_{B}T}\right).$$

The velocity of the vacancy in the lattice is expressed by:

(6) $$\nu_{i}=a(P^{\prime \prime }-P^{\prime })=a\nu_{0}\exp\left(-\frac{E_{a}}{k_{B}T}\right)\times 2\sinh\left(\frac{eFa}{2k_{B}T}\right).$$

For low electric field, *eFa* << *k*_B_*T*, taking the Taylor series expansion of sinh(*x*) ≈ *x*, the last term equals to *eFa/2k*_B_*T* and Equation (6) is simplified to:

(7) $$\nu_{i}=\frac{a^{2}eF\nu_{0}}{k_{B}T}\exp\left(-\frac{E_{a}}{k_{B}T}\right).$$

Hence, the mobility of vacancies is expressed as:

(8) $$\mu_{i}=\frac{a^{2}e\nu_{0}}{k_{B}T}\exp\left(-\frac{E_{a}}{k_{B}T}\right).$$

Combining Equations (1) and (8), the ionic conductivity can be expressed as:

(9) $$\sigma_{i}=\frac{n_{i}a^{2}e\nu_{0}}{k_{B}T}\exp\left(-\frac{E_{a}}{k_{B}T}\right),$$

which can be simplified (Arrhenius equation), in which the first term *σ_0_* *= n*_i_*a*^2^*e*^2^ν_0_/*k*_B_*T* is the conductivity pre-factor:

(10) $$\sigma_{i}=\sigma_{0}\exp\left(-\frac{E_{a}}{k_{B}T}\right).$$

Note that, in polycrystalline materials, *E*_a_ appears to be dependent on the crystallite size. The Nernst–Einstein relation relates the ionic conductivity to the diffusion coefficient of ions as:

(11) $$\sigma_{i}=\frac{n_{i}e^{2}D_{i}}{k_{B}T}.$$

The typical Arrhenius plot for an idealized ionic conductor shown in Figure 2b presents two regions. At low temperature, the conductivity (activation energy *E*_m_) is dominated by the mobility of extrinsic defects. The carrier (ion) concentration is fixed by doping. For example, an improved conductivity of 0.5 mS cm^−1^ at room temperature was obtained for Li_6_PS_5_Cl doped with few mol% of LiCl. At high temperature, the conductivity is due to thermally formed intrinsic defects. The carrier concentration varies with temperature and the slope reflects the activation energy *E*_a_, required for the creation of vacancies. *E*_a_ is obtained from the slope of the semi-logarithmic Arrhenius plot (Equation 10):

(12) $$\ln\sigma_{i}=\ln\sigma_{0}-\frac{E_{a}}{k_{B}T},$$

(13) $$E_{a}=\frac{\Delta \ln\sigma_{i}}{\Delta \left(\frac{1}{T}\right)}\times k_{B},$$

with *k*_B_ = 1.38 × 10^−23^ J K^−1^, *E*_a_ is expressed in Joule or in eV (using the conversion 1 eV = 1.6 × 10^−19^ J).

In many substances, not only in solid polymer electrolytes (SPEs) and ionic conducting glasses (ICGs) but also in Li_0.5_La_0.5_TiO_3_ perovskite-type FICs [125], for example, the ionic conductivity does not follow the Arrhenius law due to strong ion–ion interactions. The temperature dependence of the dc conductivity can be fitted to an empirical Vogel–Fulcher–Tamman (VFT) function of the form:

(14) $$\sigma_{i}=\frac{A}{\sqrt{T}}\exp\left(-\frac{B}{k_{B}\left(T-T_{0}\right)}\right),$$

where *A* is the pre-exponential factor, *B* is the activation energy, and *T*_0_ is the temperature at which the free volume to transfer Li^+^ ions is zero. Usually, *T*_0_ is the same as the glass transition temperature (*T*_g_) in SPEs or glassy electrolytes. The ‘‘nonexponentiality’’ observed in electrical conductivity relaxation has been examined using several models, such as the coupling model [126], diffusion-controlled model, [127] or the jump relaxation model [128].

### 2.2. Ionic Transport Models

Several classes of transport models for the high ionic conduction in FICs have been developed (for a summary, see [129]). Thus, theories, i.e., discrete and continuous models of conduction, have played a central role in the field of FICs for optimization of materials. The reader can find a detailed description in review articles by Mahan [130], Boyce and Huberman [131], Dieterich et al. [132], and Geisel [133]. Specific and indirect assumptions are involved in most of the models such as microstructure, distribution, and local environment of ions.

- Continuous models are concerned with the motion of ions as Brownian particles in periodic potential. This approach allows the complete description of the dynamics of superionic conductors and explains the local motion in vacant sites of the host lattice (i.e., the local motion includes relaxation and oscillating processes).
- Discrete models are hopping or random-walk models, which have long been used to study diffusion processes. There are rather simple, and a complete discussion of their dynamical properties is possible. The situation is the following: The lattice defines a periodic array of sites where the mobile ions can sit. An ion placed at one site is licked out of it after a certain time and hops away. Discrete models are applied to ionic conductors where the diffusing ions are well localized about given lattice sites over most of the time.

A common feature of these models is the fact that only the sub-system of diffusing ions is treated explicitly. This simplification can be justified by the fact that usually the characteristic rate τ^−1^ for particle jump is much smaller than a typical lattice vibrational frequency ω_D_ with ω_D_ τ >>1 [132].

Transport models proposed to explain the high ionic conductivity include the weak electrolyte model [134], the random site model [135], the dynamic structure model [136], the diffusion pathway model [137], the modified random network (MRN) model [138], the dynamic cluster model [139], the cluster-bypass model [140], jump relaxation model [141], lattice-gas model [132], and liquid-like model [132]. These models are briefly presented as follows.

The *weak electrolyte model* proposed by Ravaine [134] is applied for the ionic transport in materials with lack of long-range order (glasses). Conversely, *µ*_i_ is assumed to be independent of ion concentration, and only weakly temperature dependent, whereas *n*_i_ depends strongly on both concentration and temperature.

The *random site model* considers the existence of a wide continuous distribution of alkali ion sites of differing free energies. A clear distribution between mobile and immobile species cannot be made; thus, in this case, the summation of conductivities (Equation (1)) must be performed over the entire distribution of ions [135].

In the *dynamic structure model* reported by Maas et al. [136], the ion transport in glass is presented by postulating the existence of a *site memory effect* to visualize the formation of conducting pathways. This quantitative theory explains the general occurrence of the mixed cation (alkali) effect in glassy material and, in addition, shows that the anomalous dependence of conductivity on the modifier content in single alkali glasses follows a simple power-law relation.

In the *diffusion pathway model*, the spatial dependence of the conductivity is understood by the possible ion transport in the grains and at the grain boundaries, including intergranular pathways within and between grains. Polycrystalline model can quantify the impact of grain boundaries on conductivity as a function of grain size. Such insights provide valuable fundamental understanding of the role of grain boundaries. The lowest energy of grain boundaries the higher electrochemical performance.

The *modified random network (MRN) model* is appropriate to describe the ionic transport in glasses [138], which comprise two interlacing sublattices: Domains constructed from network former and inter-network regions made up of modifier. For example, in oxide glasses, the strong correlations associated with the network forming units masked the weak correlations between modifying cations and the oxygen sublattice.

The *dynamic cluster model* [139] is based on the idea that ion-hopping processes are directly coupled to localized structural relaxations occurring in glass even below *T*_g_, while the *cluster-bypass model* [140] states that ion diffusion occurs within microregions or clusters of material resembling to crystal. In the *jump relaxation model* described by Funke [141], two competing relaxation processes are considered after each initial forward hop of a charged defect: The backward hop of the defect and the forward motion of the surrounding “defect cloud”. The model yields the power-law of the frequency dependent conductivity.

In the *lattice gas model*, the role of ion interactions with respect to static properties is most easily investigated by considering the system of conducting ions as a lattice gas. Such a model is characterized by a Hamiltonian, which gives the energy of the various possible configurations. Each configuration is specified by a set of occupation numbers referring to the different lattice sites [132].

The *liquid-like model* is applicable to the best ionic conductors characterized by very low potential barriers *W*_B_ ≈ *k*_B_*T*, where *T* ≈ 10^3^ K [132]. Therefore, the probability of finding an ion between preferred lattice sites becomes non-negligible and a discrete lattice gas model is no longer adequate. The mutual repulsion of ions leads to an effective single particle barrier, which differs from the bare potential *W*_B_. Such effect is important with respect to transport properties and its discussion requires a continuous many-particle model. The statics of continuous systems to be described is that of a liquid embedded in a periodic medium, for which the total energy is the sum of the periodic single particle potential determined by the forces acting between mobile ions and the cage ions and the pair potential, wich consists of a short-range repulsive part, the Coulomb part, and a phonon mediated part.

The *bond-valence method* has been used to model both absolute ionic conductivity and activation energy from the “pathway volume” approach. This pathway volume–conductivity relation was found to hold for glassy and crystalline FICs with silver ion conductivities [142] and La_2/3−_*_x_*Li_3_*_x_*TiO_3_ [143]. Due to the disordered Li sublattice, the Li^+^ ionic conduction in garnet-type electrolytes is facilitated by a cooperative-type migration instead of a single hopping process with a very small time-scale for fluctuations at intermediate positions [144]. This mechanism was investigated by *ab-initio* and classical molecular dynamics (MD) studies [145,146]. In the *jump diffusion model*, the dynamics of the hopping motion of the mobile ions was investigated by Bruesch et al. [147] considering the Brownian motion in a periodic lattice that included the effect of polarizability of the lattice and correlated jumps of ions relevant to superionic conductors. In a modified model, Funke [148] has taken into account the repulsive interaction between mobile ions resulting in a “cage effect”. Because of the cage effect, the ions tend to stay at some distance from each other.

### 2.3. Impedance Spectroscopy

Ionic conductivity of the solid-state electrolytes is generally measured by the ac complex impedance method (i.e., electrochemical impedance spectroscopy (EIS)). All samples are analyzed within wide range of temperature with a small bias amplitude of 5–10 mV in the frequency range of 10^6^ Hz ~10^−2^ Hz (pulsation ω). Data are analyzed from the Nyquist plot (−*Z*″ vs. *Z*′), the imaginary part −*Z*″(ω) (capacitive) of the impedance against the real part *Z*′(ω) (resistive) [149,150]. The conductivity σ_i_ (in Scm^−1^) is calculated using the equation:

(15) $$\sigma_{i}=\frac{1}{R_{b}}\frac{d}{S},$$

where *d* denotes the electrolyte thickness (in cm), *S* is the cross-sectional area of the electrode (in cm^2^), and *R*_b_ is the bulk electrolyte resistance (in Ω). 

**Figure 3:**
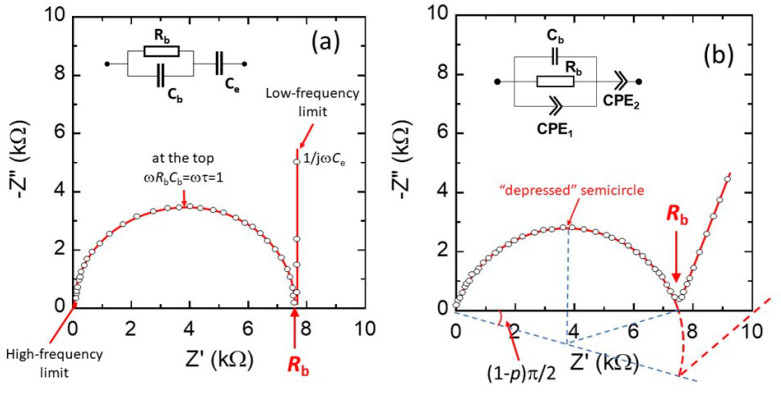
(**a**) Nyquist impedance plot of an idealized fast-ionic conductor (FIC). The semicircle centered at *R*_b_/2 represents the response of the *R*_b_, *C*_b_ parallel element and straight line is the capacity of the electrolyte/electrode interface of impedance 1/*j*ω*C*_e_. (**b**) Nyquist impedance plot of a FIC sample. The depressed semicircle reflects the combination of *R*_b_, *C*_b_, *CPE*_1_ and the inclined straight line represents the double-layer capacity of the inhomogeneous electrode surfaces.

For an idealized FIC, the bulk resistance is the quantity obtained from the diameter of the semicircle in the Nyquist plot as shown in Figure 3a. The vertical line in the low-frequency region reflects the capacity formed by the dielectric FIC sandwiched between two metallic electrodes. The equivalent circuit model (inset) consists of the parallel combination of the bulk resistance *R*_b_ and the geometry capacity *C*_b_ of the FIC (parallel plate capacitor) expressed by:

(16) $$C_{b}=\frac{\varepsilon^{\prime }\varepsilon_{0}S}{d},$$

where *ε*′ is the permittivity of the material and *ε*_0_ is the free-space permittivity (8.854 × 10^−14^ F cm^−1^). This *R*_b_,*C*_b_ element is in series with a capacity of impedance 1/*j*ω*C*_e_ (*j* = √−1), which represents the electrolyte/electrode interface). The ideal impedance of the bulk *Z*_b_ is given by the expression:

(17) $$Z_{b}=\frac{R_{b}}{1+j\omega R_{b}C_{b}}=\;{Z^{\prime }}_{b}\;+\;j{Z^{\prime \prime }}_{b},$$

where *Z*′_b_ and *Z*″_b_ are the real and imaginary part of the bulk impedance. The −*Im*(*Z*_b_) vs. *Re*(*Z*_b_) plot exhibits a standard semicircle centered at *R*_b_/2. The real and imaginary parts of the impedance are given by Equations (18) and (19):

(18) $${Z^{\prime }}_{b}=\frac{R_{b}}{1+\omega^{2}R_{b}^{2}C_{b}^{2}},$$

(19) $${Z^{\prime \prime }}_{b}=-\frac{\omega R_{b}^{2}C_{b}}{1+\omega^{2}R_{b}^{2}C_{b}^{2}}.$$

The experimental Nyquist plot of a FIC sample placed between two stainless-steel electrodes is shown in Figure 3b. This diagram deviates from the ideal impedance spectrum as the capacitor in EIS experiments often does not behave ideally. The impedance spectrum consists of a depressed semicircle, which can be visualized by the equivalent circuit including the parallel association of the bulk resistance *R*_b_ with the capacitance *C*_b_ and a constant phase elements (CPE_1_), which represents the geometry capacity and the effects of dipolar relaxation (i.e., system with a distribution of time constants), respectively. Similarly, CPE_2_ replaces the pure C_e_ capacitance due to surface roughness of the electrode/FIC interfaces. The impedance of a CPE is expressed as:

(20) $$Z_{CPE}=T{\left(j\omega \right)}^{-p}=T\omega^{-p}\left[\cos\left(\frac{p\pi }{2}\right)-j\sin\left(\frac{p\pi }{2}\right)\right],$$

where *p* is the exponent of CPE (0 < *p* ≤ 1) and *T* is the CPE constant (10^−3^ < *T* < 10^−6^). The constant phase is *ϕ* = − *p*π/2.

**Figure 4:**
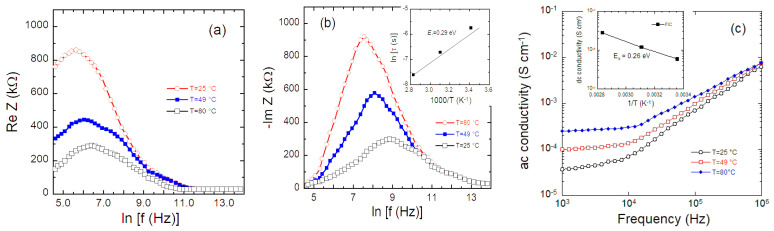
(**a**) Plot of Re(*Z*) vs. ln (*f*). (**b**) Plot of -Im(*Z*) vs. ln (*f*) and (inset) determination of the activation energy *E*_τ_ of the relaxation time. (**c**) Frequency dependence of the ac conductivity and (inset) determination of the activation energy *E*_a_ of σ_dc_.

Figure 4a,b show the frequency dependence of the real *Z*′(ω) and imaginary −*Z*”(ω) part of the impedance, respectively, of a FIC sample measured at three temperatures. At ω > 10^3^ Hz, the plots of Figure 4a show a decrease of Z′ vs. frequency, so that σ(ω) increases with frequency (see Figure 4c). At low frequency (*f* ≈ 1 kHz), σ(ω) increases importantly with temperature. At high frequencies, however, Z′(ω) becomes almost temperature independent so that the *Z*′(ω) curves at different temperatures merge approximately in a single curve. This is due to the release of space charges caused by reduction in barrier properties of the material [151,152]. This unique curve at high frequency shows a dip, which is associated with charge carrier hopping in the material. On the other hand, *Z*″ = −Im(*Z*(ω)) reaches a maximum, which shifts towards higher frequency with temperature. This is attributed to the active conduction through the grain boundaries of the sample. The peak broadening observed with increasing temperature is attributed to a temperature-dependent relaxation process in the material. The asymmetric broadening of the peaks indicates the spread of relaxation time in the sample.

The frequency *f*_m_, at which −Im(*Z*(ω)) goes through a maximum, corresponds to the single relaxation time, which fulfills the relation 2π*f*_m_τ_m_ = 1. For a thermally activated relaxation process, the variation of τ with *T* obeys an Arrhenius law given by [153,154]:

(21) $$\tau =\tau_{0}\exp\left(\frac{E_{\tau }}{k_{B}T}\right),$$

where τ_0_ is the pre-exponential factor and *E*_τ_ is the activation energy. The inset in Figure 4b shows the temperature dependence of the relaxation time of FIC sample. When the mean relaxation time of the process is measured in fraction of milliseconds, it implies slow relaxation, which can be imposed by permanent molecular dipoles, ion defects of a dipolar type, or mobile hopping charge carriers [31]. 

The ac conductivity σ_ac_ (Figure 4c) obeys the power law [153]:

σ(ω) = σ_ac_ = σ_0_ + *A* ω^n^, (22)

where σ_0_ is the dc conductivity (at ω ≈ 0), *A* is a thermally activated quantity, and *n* is the fractional constant, which is 0.5 < *n* < 0.8 for an ionic conductor [155]. The frequency exponent *n* (Equation (4)) can be analyzed by a mechanism based on charge carrier hopping between defect sites proposed by Elliott [156]:

(23) $$n=\frac{\partial (\ln\sigma_{ac})}{\partial (\ln\omega )}=1-\frac{6k_{B}T}{E_{m}},\;$$

where *E*_m_ is the maximum barrier height (energy of the transport charge). Using Equation (22), from the slope of curves in Figure 4c, one can derive at the highest frequency with *n* and the value of *E*_m_ at room temperature.

**Figure 5:**
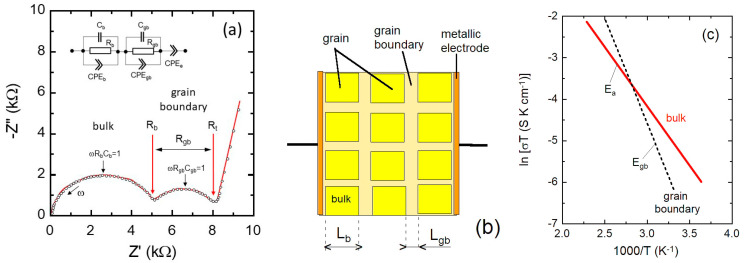
(**a**) Impedance spectrum of a polycrystalline FIC. The equivalent circuit employed to fit the Nyquist plot is shown in inset. (**b**) Scheme of the” brickwork model” of intra- and intergrains in ceramic placed between two metallic electrodes for impedance measurements. (**c**) Typical Arrhenius curves of the conductivities for bulk and grain boundary showing increased intergain activation energy.

In practice, solid electrolytes are mainly polycrystalline ceramics with a microstructure composed of intragrains (bulk) of dimension *L*_b_ separated from each other by a boundary (intergrain) of thickness *L*_gb_ [102,157]. The typical impedance spectrum of polycrystalline FIC (Figure 5a) displays two distinct depressed semi-circles: In the high-frequency range attributable to bulk (intragrain) and in medium-frequency region assignable to grain boundary (intergrain) domains [157]. Thus, the Nyquist plot can be visualized by the equivalent circuit (inset in Figure 5a) including the additional parallel association of the intergrain resistance *R*_gb_ with the capacitance *C*_gb_ and a constant phase elements (CPE_gb_). The value of *R*_gb_ is obtained from the difference of the intercepts on the Z′ axis:

*R*_gb_ = *R*_t_ − *R*_b_, (24)

where *R*_t_ is the total resistance and *C*_gb_ is calculated by applying the equation of the frequency at the semi-circle maximum (ωR_gb_C_gb_ = 1). Irvine et al. [158] considered the factors controlling the magnitude of the grain boundary impedance using a “brickwork model” (Figure 5b) for an idealized ceramic with cube-shaped grains separated by intergrains of impedance *Z*_gb_. From the inverse relation between dielectric thickness and capacitance (Equation 16), for this idealized case, Equation (25) indicates the quality of the sintering and the nature of the narrow intergranular regions:

(25) $$\frac{C_{b}}{C_{gb}}=\frac{L_{gb}}{L_{b}}.$$

For well-sintered samples, generally, the overall impedance of intergrains is 2–3 times greater than the impedance of grains. Typical Arrhenius plot of the conductivities of bulk and grain boundaries is shown in Figure 5c, which display different conduction mechanisms with increase of the intergrain activation energy (*E*_gb_ > *E*_a_).

## 3. Sulfide Solid Electrolytes

Owing to their high Li^+^ ion conductivity at room temperature, sulfide-based materials are more promising electrolytes than oxide-based ones [159,160,161,162,163,164,165,166,167,168,169,170,171,172,173,174,175,176,177,178,179,180,181,182,183,184,185,186,187,188,189,190,191,192,193,194,195,196,197,198,199,200,201,202,203,204,205,206,207,208,209,210,211,212,213,214,215,216,217,218,219,220,221,222,223,224,225,226,227,228,229,230,231,232,233,234,235,236,237,238,239,240,241,242,243,244,245,246,247,248,249,250,251,252,253,254,255,256,257,258,259,260,261,262,263,264,265,266,267,268,269,270,271,272,273,274,275,276,277,278,279,280,281,282,283,284,285,286,287,288,289,290,291,292,293,294,295,296,297,298,299,300,301,302,303,304]. In addition, sulfide-based electrolytes are relatively soft and deformable. Furthermore, the polarizability of sulfide-based electrolytes is higher than that of oxide-based electrolytes, which leads to the attraction between the Li^+^ ions and sulfide framework being weaker than that between the Li^+^ ions and oxide framework and the mobility of sulfide-based electrolytes being higher than that of the oxide-based ones. In 1996, Otto [159] reported that the conductivity of the Li_2_O–Li_2_Cl_2_–Li_2_SO_4_–SiO_2_–B_2_O_3_ (35:10:30:12.5:12.5) glass system was 3.3 × 10^−6^ and 9.7 × 10^−2^ S cm^−1^ at 25 and 350 °C, respectively. In 1997, Calès et al. [160] reported ionic conductivities of 1.0 × 10^−3^ S cm^−1^ at 300 °C for the B_2_O_3_–Li_2_O–Li*X* (*X* = F, Cl, Br, I) and B_2_O_3_–Li_2_O–Li_2_SO_4_ borate-based glassy electrolytes; their publication led the search for new sulfide-based electrolyte systems. In 1981, Mercier et al. [161] reported that the room-temperature conductivity of Li_2_S–P_2_S_5_–LiI (Li_4_P_2_S_7_∙LiI) was 10^−3^ S cm^−1^. In 1986, Pradel and Ribes [162,163] studied *x*Li_2_S(1−*x*)SiS_2_ (*x* ≤ 0.6) and Li_2_S–*M* (*M* *=* SiS*_2_,* GeS*_2_,* P*_2_*S*_5_,* B*_2_*S*_3_,* As*_2_*S*_3_*) glasses. Furthermore, in 1986 and 1987, Kennedy [164,165] reported the melt quenching synthesis method and performed conductivity studies on Li_2_S–SiS_2_ Li*X* (*X* = Br, I); in addition, in 1988 and 1989, Kennedy and Zhang [166,167] investigated the SiS_2_–P_2_S_5_–Li_2_S–Li_2_S–LiI system, where Li*X* acted as an interstitial dopant to improve the ionic conductivity. Rao and Seshasayee [168] conducted molecular dynamics (MD) simulation studies of the *x*(0.4Li_2_S–0.6P_2_S_5_)–(1 − *x*)LiI and *x*(0.5Li_2_S–0.5P_2_S_5_)–(1 − *x*)LiI (*x* = 0.9, 0.75) superionic sulfide glasses ternary systems and attributed their high room-temperature ionic conductivity to the presence of non-bridging S atoms around the diffusing Li atoms. Moreover, the decrease in the glass transition temperature (*T*_g_) of these systems was ascribed to the presence of iodine atoms, which led to the plasticization of the structure, rendering it less rigid and decrease in P–P bonds caused by the modifying action of the Li atoms, which also weakened the glass matrix and contributed to the decrease in *T*_g_.

From 1986 to 1989, Akridge and Vourlis [169], Balkanski et al. [170], Meunier et al. [171], Creus et al. [172] and Jones and Akridge [173,174] introduced and developed the thin-film electrolyte concept. In 1995, Takada et al. [175] reported that when ASSBs featuring thin-film cells with the Li*M*O_2_ (*M* = Co, Ni)/Li_3_PO_4_ (LPO)–Li_2_S–SiS_2_/Li metal electrochemical chain, were cycled at a current rate of 64 µA cm^−2^ in the voltage range of 2.0–3.8 V, their capacity ranged from 80–90 mAh g^−1^. Subsequently, different glassy and nanocrystalline sulfide-based electrolytes have been explored by researchers worldwide.

Many research groups studied Li–P–S-based glasses, glass-ceramics, argyrodites, Li_6_PS_5_*X* (*X* = Cl, Br, I), thio-LISICONs, and Li_11−*x*_*M*_2−*x*_P_1+*x*_S_12_ (*M* = Ge, Sn, and Si) as electrolytes [176,177]. Among all reported electrolyte compositions, Li_6_PS_5_Cl, β-Li_3_PS_4_ (β-LPS), and Li_7_P_2_S_8_I have been the most studied owing to their excellent conductivity and remarkable mechanical properties, which facilitated the fabrication of ASSBs. Few reviews, such as those published by Zhang et al. [176] and Takada [177] focused on sulfide-based electrolytes. Herein, we highlight the most important recent studies and focus more on the fabrication technologies, importance of stack pressure on different electrolyte systems, and role of the electrode and cell fabrication techniques on the electrochemical properties of ASSBs.

### 3.1. Argyrodite Electrolytes

In 2008, Deiseroth et al. [178] introduced a new Li_6_PS_5_*X* (*X* = Cl, Br, I) Li-argyrodite fast-ion conductor and reported that the preliminary room-temperature conductivity values of this material were in the range of 10^−2^–10^−3^ Scm^−1^. This work opened the avenue for the further understanding of the structural and physical properties of solid-state electrolytes and facilitated the development of ASSBs. Argyrodite presents high conductivity; moreover, argyrodite-based batteries are easier to fabricate than those featuring oxide-based solid electrolytes, and therefore, below, we summarize a series of reports on the synthesis, fabrication, and interfacial properties of argyrodite electrolytes [179,180,181,182,183,184,185,186,187,188,189,190,191,192,193,194,195,196,197,198,199,200,201,202,203,204,205].

**Figure 6:**
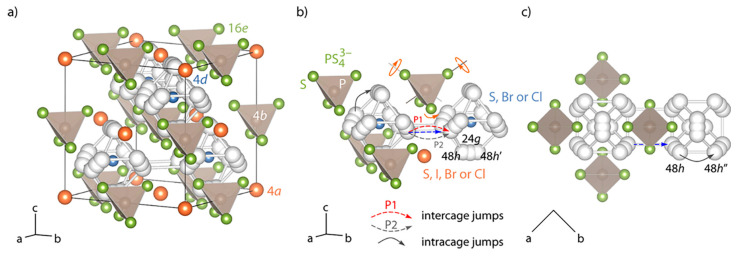
(**a**) Crystal structure of argyrodite-type Li_6_PS_5_*X* that crystallizes with cubic symmetry in the space group *F*43*m*. In Li_6_PS_5_Cl, the Li^+^ ions solely occupy the 24*g* positions of the split site 48*h*−24*g*−48*h*′. In compounds with *X* = Br and I, they are distributed over the 24*g* sites and the 48*h* positions. P resides on 4*b*. The 16*e* is fully occupied by S^2−^ forming PS_4_^3−^ tetrahedra. Whereas in Li_6_PS_5_I, the halide anions occupy only the 4*a* sites; in Li_6_PS_5_Br, the occupation factors, according to neutron diffraction, amount to 78% (4*a*) and 22% (4*d*). For Li_6_PS_5_Cl, the occupation factors are 39% (4a) and 62% (4*d*); thus, the majority of the Cl anions occupy the inner centers of the Li cages, which are too small for I^−^. (**b**) Intracage and intercage Li diffusion pathways: Hopping between two Li cages (48*h*−48*h*″, see also (**c**)), either following a direct or curved pathway, could be influenced by S^2−^ anions of a nearby PS_4_^3−^ tetrahedral. The jump distance depends on the lattice constant and, thus, on halogen substitution. Possible rotational jumps are indicated that may open or block the Li^+^ pathway. (**c**) The same cutout as in (**a**) but viewed along the c-axis. Two S^2−^ anions of the PS_4_^3−^ tetrahedra are located slightly above the direct 48*h*−48*h*″ exchange pathway. Rotational jumps of the PS_4_^3−^ tetrahedra could also influence the intracage jumps. Reproduced with permission from Ref. [179]. Copyright 2019, American Chemical Society.

(i) Li_6_PS_5_*X* (*X* = Cl, Br, I) compounds are isostructural with Cu- and Ag-argyrodite materials with cubic unit cells (*F-*43*m* space group) (Figure 6a–c) [179]. In this cubic structure, Li^+^ ions are randomly distributed over the remaining tetrahedral interstices (48 *h* and 24 *g* Wyckoff sites), in which P atoms occupy the tetrahedral interstices (4*b* sites), while 16*e* sites are fully occupied by S^2−^ forming a network of isolated PS_4_ tetrahedra. *X* anions form a face centered cubic (fcc) lattice (4*a* and 4*c* sites). Li occupy the 24*g* site in the Li_6_PS_5_Cl lattice, whereas they are distributed over the 24*g* and 48*h* sites in the Li_6_PS_5_Br framework [180]. Li^+^ ion diffusion occurs via these partially occupied positions, which form hexagonal cages connected to each other via the interstitial sites around the *X*^−^ and S^2−^ ions for Li_6_PS_5_Cl and Li_6_PS_5_I, respectively. Rao and Adams [181] reported that the lattice parameters of the polycrystalline Li_6_PS_5_Cl, Li_6_PS_5_Br, and Li_6_PS_5_I powders were *a* = 9.85, 9.98, and 10.142 Å, respectively. Observed differences in the lattice parameter values are due to differences in the ionic radii (*r*) of the anions in Li_6_PS_5_X, i.e., *r*(S^2^^−^) = 1.84 Å, *r*(Cl^−^) = 1.81 Å, *r*(Br^−^) = 1.95 Å, and *r*(I^−^) = 2.16 Å.

(ii) In 2011, Rao and Adams [181] and Rao et al. [182] synthesized Li_6_PS_5_*X* (*X* = Cl, Br, I) and performed neutron diffraction, conductivity, and bond valence computational studies on them. They reported the presence of a three-dimensional (3D) pathway network for the long-range ion conduction of all Li_6_PS_5_*X* (*X* = Cl, Br, I) phases, which consisted of interconnected low-energy local pathway cages [180]. The experimentally measured ionic conductivity at 25 °C of Li_6_PS_5_Cl, Li_6_PS_5_Br, and Li_6_PS_5_I prepared by ball milling followed by heating at 550 °C in inert atmosphere are in the range 1.9 × 10^−4^–7.0 × 10^−3^ S cm^−1^ and calculated activation energies in the range 0.26–0.41 eV (Table 1) [180,181,182,183,184,185,186]. Further, Boulineau et al. [183] reported the effect of enhancement of the conductivity of Li_6_PS_5_Cl from 2× 10^−4^ S cm^−1^ to 1.33 × 10^−3^ S cm^−1^ when the ball milling time varies from 1 h to 10 h. Rao and Adams [181] compared the values of *E*_a_ determined by both experimental and computational method for Li_6_PS_5_*X* with *X*= Cl, Br, I in the range 0.25–0.38 eV. Camacho-Forero and Balbuena [184] performed ab initio calculations and determined that conductivity, activation energy, and the diffusion coefficient of Li^+^ ions at 27 °C were 0.17 × 10^−3^ S cm^−1^, 0.37 eV, and 1.2 × 10^−9^ cm^2^s^−1^ for Li_6_PS_5_Cl and 6.07 × 10^−3^ S cm^−1^, 0.27 eV, and 5.8 × 10^−9^ cm^2^s^−1^ for Li_6_PS_5_I, respectively. The reported diffusion coefficient value of Li_6_PS_5_Cl was reported to be two orders of magnitude lower than that determined using ^7^Li nuclear magnetic resonance (NMR) (7.7 × 10^−8^ cm^2^s^−1^ at 40 °C) [179]. According to Camacho-Forero and Balbuena [184], the ionic conductivity of Li_6_PS_5_I was significantly lower than those of Li_6_PS_5_Cl and Li_6_PS_5_Br.

(iii) Argyrodite electrolytes can be synthesized using different methods [169,178,179,180,181,182,183,184,185,186,187,188,189,190,191,192,193,194,195,196,197,198,199,200,201,202,203,204,205,206,207,208], such as the conventional sealed tube solid-state reaction [169], ball milling [181,183,187], and solution-based methods [189,208].

(iv) The conductivities of argyrodite electrolytes depend on the preparation method, grain boundary contributions, and conductivity measurement method and fabrication technique of pelletized samples, including sintering cold-pressed pellets that influences the density of the specimens [183]. Based on previous literature studies, conductivity values are also influenced by cooling rate [186], porosity, and pore distribution [190]. Lower Li^+^ ion conductivities, in the range of 10^−5^–10^−4^ mS cm^−1^, were reported when the electrolytes were synthesized via the solution-based method, which were attributed to the presence of additional impurity phases in the compounds [189].

(v) Deiseroth et al. [185], Yu et al. [191,192,193], Hanghofer [179], Ganapathy et al. [194], Epp et al. [197], and Adeli et al. [198] used the solid-state NMR method to characterize the structure and dynamics. Results of the chemical shifts from ^31^P and ^6^Li MAS NMR spectra [179] are 85 and 1.6 ppm for *X* = Cl, 93.9 and 1.49 ppm for *X* = I, and 96.3 and 1.3 ppm for *X* = Br nanostructured samples synthesized by the solid-state and ball milling methods. The conductivity, *E_a_*, and Li-jump rate values obtained from NMR measurements were 10^−3^–10^−2^ S cm^−1^, 0.2 eV, and 10^9^ s^−1^, respectively, for Li_6_PS_5_Br and Li_6_PS_5_I [197].

(vi) The reported electrochemical stability potential window of Li_6_PS_5_*X* (*X* = Cl, Br, I) was determined to be 0–7 V vs. Li^+^/Li [20,176,177].

(vii) Kong et al. [199] determined that the substitution of S with O in Li_6_PS_5_*X* (*X* = Cl, Br) led to the decrease in room-temperature conductivity by several order of magnitudes, to ~10^−9^ S cm^−1^; moreover, the *E*_a_ of the O-containing compound was 0.66 eV. The observed low conduction mechanism was further confirmed by Rao and Adams [181] using bond valence studies.

(viii) Kasemchainan et al. [200] and Doux et al. [201] reported the critical current density limits for Li plating on Li_6_PS_5_Cl and studied the stack pressure limits of Li_6_PS_5_Cl, respectively.

(ix) Yokokawa [202] examined the thermodynamic stability of the sulfide electrolyte/oxide interface of ASSBs; they proposed a potential diagram approach, in which the phase relationships at the interfaces could be investigated by comparing the proper chemical potentials associated with the target devices. Understanding the aforementioned parameters is crucial for both fundamental and industrial applications.

(x) In 2019, Rao et al. [188] reported the new Li_15_(PS_4_)_4_Cl_3_ and Li_14.8_Mg_0.1_(PS_4_)_4_Cl_3_ phases with the *I*-43*d* space group and lattice parameters *a* of 14.308 and 14.323 Å, respectively, which were isostructural with the Ag_15_(PS_4_)_4_Cl_3_ phases; in addition, they reported that Mg^2+^ doping led to the increase in ionic conductivity from 4 × 10^−8^ S cm^−1^ for Li_15_(PS_4_)_4_Cl_3_ to 2 × 10^−7^ S cm^−1^ for Li_14.8_Mg_0.1_(PS_4_)_4_Cl_3_.

**Figure 7:**
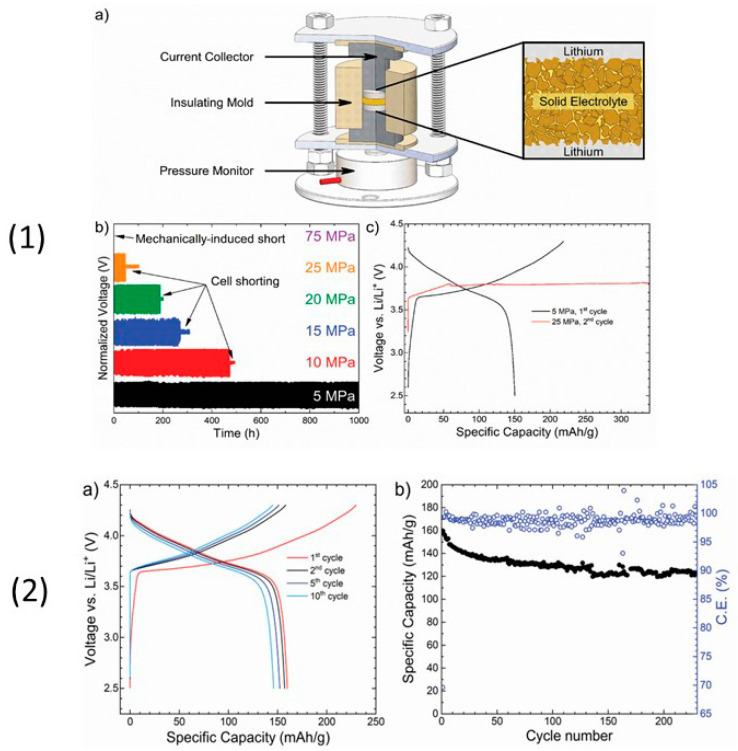
(**1**) (**a**) Design of solid-state Li symmetric cell that allows control and monitoring of the pressure during cycling. (**b**) Normalized voltage of Li symmetric cells as a function of the plating and stripping times at different stack pressures. At 75 MPa, the cell already mechanically short-circuited before cycling began. At 5 MPa, no short-circuit was observed for over 1000 h. (**c**) Voltage profile of a full cell with Li metal anode. The first cycle was run at a stack pressure of 5 MPa. The stack pressure was subsequently increased to 25 MPa before the second cycle, during which the cell short-circuited. (**2**) (**a**) Voltage profiles of the 1st, 2nd, 5th, and 10th cycles and (**b**) cycle life of a Li metal|Li_6_PS_5_Cl|LiNbO_3_-coated LiNi_0.8_Co_0.15_Al_0.05_O_2_ all-solid-state Li-ion battery cycled at C/10 and a stack pressure of 5 MPa (black and blue dots are specific capacity and coulombic efficiency data, respectively). No short-circuiting behavior was observed. The average Coulombic efficiency (C.E.) over 229 cycles was 98.86%, and the capacity retention of the cell was 80.9% over 100 cycles. The active material loading was 3.55 mg cm^−2^. Reproduced with permission from [201]. Copyright 2020 Wiley.

Many reports have been published on Li_6_PS_5_*X* (*X* = Cl, Br, I) sulfide electrolytes for ASSBs. Herein, we highlight one of the recently published reports. Kasemchainan et al. [200] studied the effect of the current density (0.1–4.0 mA cm^−2^) and pressure (3 and 7 MPa) on Li|Li_6_PS_5_Cl|Li. Recently, Doux et al. [201] studied the effect of the stack pressure on the cycling of the Li|Li_6_PS_5_Cl|Li cell and performed cycling studies on a mixture of 2 wt.% LiNbO_3_ (LNO)-coated LiNi_0.80_Co_0.15_Al_0.05_O_2_, Li_6_PS_5_Cl, and carbon black with a weight ratio of 11:16:1 that was obtained using an agate mortar and pestle. For this study, 12 mg of composite electrode was pressed on one side of the electrolyte pellet at a pressure of 370 MPa and Li-In powder or a Li metal disc were subsequently pressed at 120 or 25 MPa, respectively, on the other side of the electrolyte pellet. The effects of different stack pressures in the range of 5–25 MPa on the fabricated Li symmetric cells during plating and stripping were reported (Figure 7) [201]. The possible reasons for the good cycling are presented in the schematic diagram in Figure 7(1). It was observed that at the stack pressure of 5 MPa, no short-circuit occurred for up to 1000 h; moreover, the capacity retention of the cell was 81% after 100 cycles (Figure 7(2)). In addition, it was noted that as the pressure increased from 1 to 5, 10, 15, 20, and 25 MPa, the impedance decreased from >500 Ω, to 110, 50, 40, 35, and 32 Ω, respectively. In conclusion, at low stack pressure (5 MPa), Li plating occurred on the surface of the pellet because the pressure was not sufficient to allow Li to pass into the pores of the electrolyte. Conversely, a pressure of 25 MPa led to the surface modification of the electrolyte pellet, in which Li^+^ ions passed into the pores of the electrolyte along the interface. At the high stack pressure of 75 MPa the cell underwent mechanical shorting before plating and stripping.

**Figure 8:**
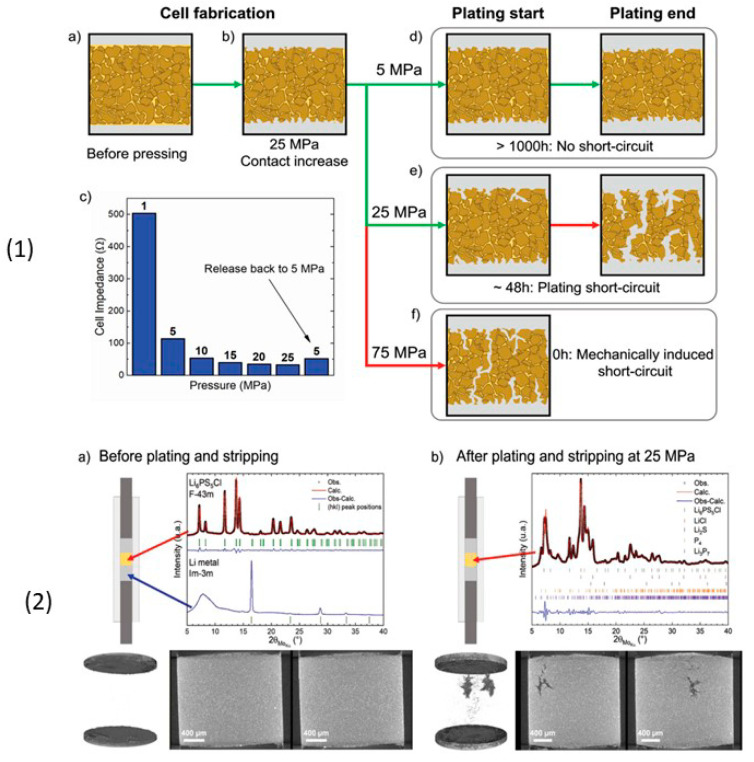
(**1**) Schematic of the effect of the stack pressure on the short-circuiting behavior of Li metal solid-state batteries. (**a**) During cell fabrication, the contact between the electrolyte and Li metal was poor before the Li metal was pressed on the electrolyte pellet. (**b**) Pressing the Li metal at 25 MPa allowed the proper wetting of the electrolyte and (**c**) induced a large decrease in the impedance of the symmetric cell even when the pressure was later decreased to 5 MPa. (**d**) Plating and stripping at a stack pressure of 5 MPa. Li did not creep inside the solid-state electrolyte (SSE) pellet, and therefore, the cell cycled for more than 1000 h. (**e**) At a stack pressure of 25 MPa, Li slowly crept between the grains of the SSE and plating occurred on the dendrites, which eventually short-circuited the cell after 48 h. (**f**) When the stack pressure was too high, Li crept through the electrolyte and formed dendrites that mechanically short-circuited the cell. (**2**) Schematic of the cell used for X-ray tomography and X-ray diffraction (XRD) analyses; profile matching of the XRD and X-ray tomography patterns of a Li|Li_6_PS_5_Cl|Li symmetric cell cycled under a stack pressure of 25 MPa (**a**) before plating and stripping and (**b**) after short-circuiting. Before plating and stripping, only Li_6_PS_5_Cl was detected in the electrolyte and Li metal was present on both sides. The X-ray tomography pictures confirmed that Li was not present in the electrolyte. After the cell short-circuited, several additional phases, mainly Li_2_S, LiCl, P_4_, and Li_3_P_7_, were detected inside the electrolyte; these were components of the solid electrolyte interphase that formed when Li was in contact with Li_6_PS_5_Cl. The X-ray tomography pictures illustrate that a large quantity of low-density dendrites formed in the electrolyte. Reproduced with permission from [201]. Copyright 2020 Wiley.

Moreover, Koerver et al. [203] and Kim et al. [204] applied high pressure in the range of 50–70 MPa on β-LPS, which led to distinct differences in the stack pressures, which affected the mechanical properties of the electrolyte. Furthermore, the structure and morphology of β-LPS were studied using XRD and X-ray tomography on 2 mm diameter with an experimental resolution of 1 µm over the entire volume. The tomography images and XRD patterns before and after the 25 MPa plating and stripping are illustrated in Figure 8(1) [201]. The tomography images after plating and stripping at 25 MPa (Figure 8(2)) illustrate large low-density structures within the electrolyte.

Furthermore, the images revealed that Li dendrites formed and propagated between the electrolyte grains along grain boundaries. Moreover, the XRD patterns revealed the presence of LiCl, Li_2_S, and other P_4_ and Li_3_P_7_ phosphorous phases in the Li_6_PS_5_Cl structure [201]. Zhang et al. [205] reported the inter- and intracycle interfacial evolution of a LiNi_0.8_Co_0.1_Mn_0.1_O_2_ (NMC)|Li_6_PS_5_Cl|Li cell using impedance measurements, Raman spectroscopy, and scanning electron microscopy (SEM) studies. Furthermore, Zhou et al. [206] studied the Li_6_PS_5_*X* (*X* = Cl, Br, I) and Li_6−*y*_PS_5−*y*_Cl_1+*y*_ argyrodites, while Feng et al. [207] investigated Li_6−*x*_PS_5−*x*_Cl_1+*x*_. Recently, Arnold et al. [208] reported an improved conductivity of 0.53 × 10^−3^ S cm^−1^ at RT for Li_6_PS_5_Cl doped with LiCl and they showed the enhanced electrochemical properties with cells assembled with Li||LTO (Li_4_Ti_5_O_12_) using bare and doped electrolyte. Although Li_6_PS_5_Cl presented good ionic conductivity, further studies on large-scale packs and the improvement in the air stability and surface protection of argyrodites are required to facilitate their large-scale applications. Transport properties of sulphide solid electrolytes, i.e., room temperature ionic conductivity s_RT_ and activation energy *E*_a_ are summarized in Table 1.

### 3.2. Lithium Phosphorus Sulfide Electrolyte

The lithium phosphorus sulfide (Li_3_PS_4_, LPS) electrolyte was derived from the (100 − *x*)Li_2_S–*x*P_2_S_5_ binary system for *x* = 25 [203,204,209,210,211,212,213,214,215,216,217,218,219,220,221,222,223,224,225,226,227,228,229,230,231,232,233,234,235,236,237,238]. The first report on LPS was published by Tachez et al. [212] in 1984; later on, Eckert et al. [213] performed solid NMR studies on these systems. It was not until 2002 that Tatsumisago et al. [214] reexplored the Li_2_S–P_2_S_5_ glass system and studied in detail its structure and storage properties. More studies on the synthesis, crystal structure, stability, and fabrication of ASSBs based on these electrolyte systems have been performed since. LPS presents three polymorphs, viz. α-, β- and γ-LPS, of which the γ and β phases presents the lowest (3 × 10^−7^ S cm^−1^) and highest (~10^−4^ S cm^−1^) conductivities, respectively. Herein, we highlight the most important observations on the β-LPS electrolyte reported in the literature as follows.

**Figure 9:**
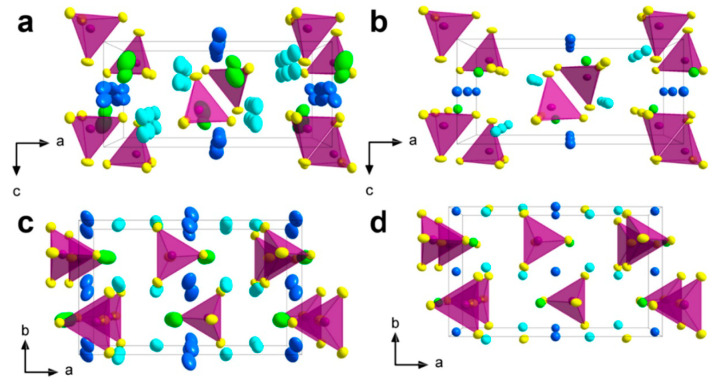
(**a**) Schematic representation of the structure of orthorhombic Li_3.25_[Si_0.25_P_0.75_]S_4_ derived from single-crystal data. (**b**) Structure of β-Li_3_PS_4_ (β-LPS) along the [010] direction. Views of (**c**) Li_3.25_[Si_0.25_P_0.75_]S_4_ and (**d**) β-LPS along the [001] direction. Here, the violet tetrahedra, turquoise spheres, Li(8*d*)-2 (blue) in Li_3.25_[Si_0.25_P_0.75_]S_4_/Li(4*b*)-2 (blue) in β-Li_3_PS_4_, green spheres, and yellow spheres denote Li(4*c*)-3A/B, and S atoms, respectively. Reproduced with permission from [222]. Copyright 2019 American Chemical Society.

(i) Eckert et al. [213], Tatsumisago et al. [214], Minuzo et al. [215], Hayashi et al. [216,217], and Murayama et al. [218] reported the synthesis of LPS using mechanical and solid-state methods, and that of glass–ceramic LPS using ball milling. The room-temperature conductivity of LPS was reported to be 3.2 × 10^−3^ S cm^−1^ (see Table 1) [229,301,302]. Subsequently, many research groups explored the composition of LPS, to elucidate the crystal structure, ionic conductivity, and fabrication of LPS-based ASSBs. Garcia-Mendez et al. [219] reported the effect of molding pressure on mechanical and ionic conductivity values of LPS electrolyte, and recently, Ohno et al. [220] summarized various other factors which influence the electrical properties of sulfate electrolytes.

(ii) Homma et al. [221] studied the crystal structure and phase transitions of LPS. High-temperature synchrotron XRD and thermal studies were used to determine that LPS exhibited three phase transitions at different temperatures. The γ, β, and α phases were present at low, medium (300−450 °C), and high (473 °C) temperature. Among all phases, the β-phase has been the most studied owing to its high ionic conductivity. Zhou et al. [222] reported that Li_3.25_[Si_0.25_P_0.75_]S_4_ is an entropically stabilized fast-ion conductor. The β-LPS phase presents orthorhombic structure with the space group *Pnma*, and its lattice parameters have been reported to be *a* = 13.066(3) Å, *b* = 8.015(2) Å, and *c* = 6.101(2) Å (Figure 9a–d) [222].

(iii) Haruyama et al. [223] analyzed the LiCoO_2_/β-Li_3_PS_4_ (LCO/β-LPS) and LCO/LNO/LPS (where LNO was the buffer layer) oxide/electrolyte interfaces using computational methods, i.e., density functional theory (DFT) and U framework studies, and determined that surface protection was essential for long-term electrochemical cycling. Their research was followed by many experimental studies on surface-coated NMC cathodes such as LNO, LPO, and Li_2_O–ZrO_2_, which were aimed at reducing the cathode/electrolyte interfacial reactions during electrochemical cycling. Few other computational studies, such as that of Richards et al. [224], who predicted the formation of the Li_3_P and Li_2_S phases at on LPS/Li interface and the formation of Co(PO_3_)_2_, CoS_2_, and S, at the LiCoO_2_/Li interface during electrochemical cycling, have been published.

Tsukasaki et al. [209,225,226] and Atarashi et al. [211] reported the synthesis, solid-state battery fabrication, electrochemical cycling, and thermal stability study of bare and coated LiNi_1/3_Mn_1/3_Co_1/3_O_2_ (NMC) and LPS electrolytes, and indicated that their reversible capacity after 50 cycles was approximately 80 mAh g^−1^. Ex situ XRD [211] and in situ synchrotron XRD [227] measurements were performed to analyze the thermal stability of LNO-coated–NMC–LPS composites. When heated above 300 °C, the NMC cathode decomposed into transition metal sulfides, such as CoNi_2_S_4_ and MnS, and led to the formation of O_2_ gas; conversely, LPS transformed to crystalline LPO owing to the oxidation reaction between the electrolyte and generated O_2_ [226]. From the aforementioned thermal studies, we concluded that the exchange reaction between S and O in LPS can be avoided by P (Li_3_PS_4_), Sn (Li_4_SnS_4_) [227], or Sb (Li_3_SbS_4_) [228], which gives strong bond strength with S and could decrease the reactivity with O_2_ and H_2_O in air. The slow reactions between Sb and Sn and Li metal to form Li_4.4_Sn or Li_3_Sb, which occur during electrochemical cycling, are possible drawbacks of these materials. Furthermore, although these electrolytes are stable in air, the Li–Sn–S electrolyte presents low conductivity of 1.5 × 10^−6^ S cm^−1^ at room temperature, which hindered the use of Sn and Sb electrolytes for SSB applications.

**Figure 10:**
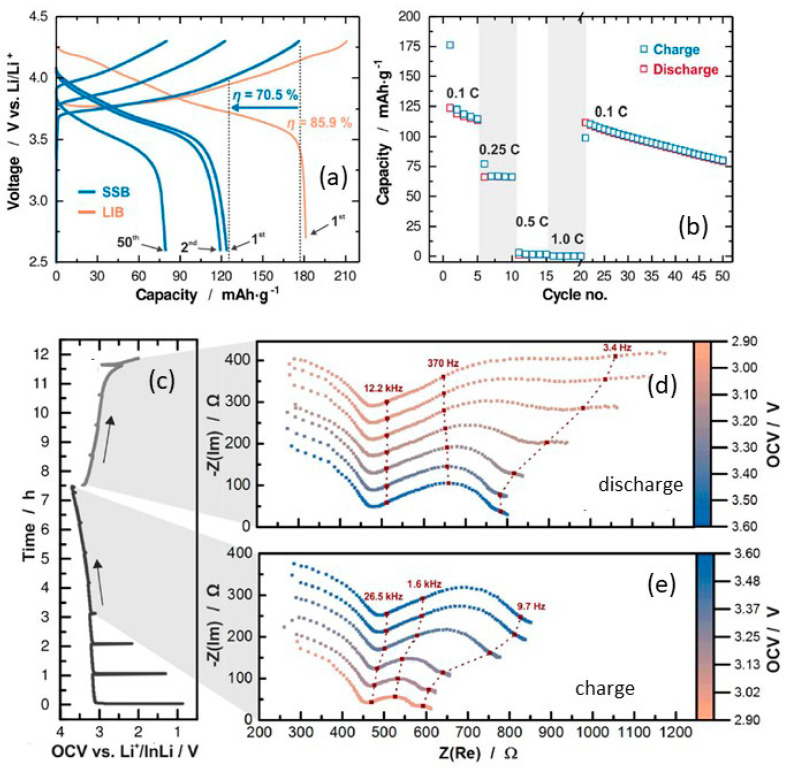
(**a**) Representative charge–discharge profiles of an ASSLB for the 1st, 2nd, and 50th cycle (blue). The ASSLB was cycled between 2.7 and 4.3 V vs. Li^+^/Li. The orange curve represents the 1st charge-discharge cycle of a liquid Li-ion battery (LIB) with NMC-811 as the cathode, and it was included for comparison. The current density for all cycles was 0.1C. (**b**) Rate test and long-term cyclability of the SSB at the current densities of 0.1, 0.25, 0.5, and 1C, followed by open-end cycling at 0.1C. A large first cycle capacity loss was observed for the ASSLB, which did not occur for the LIB. Impedance spectra recorded intermittently during galvanostatic battery cycling. (**c**) First cycle charge and discharge profile of a Li–In|β-Li_3_PS_4_|NCM811/β-Li_3_PS_4_ cell at 0.1 C showing current interruption corresponding to the periods of impedance measurement. Impedance spectra during (**d**) charge and (**e**) discharge periods. Measurements were conducted after 1 h of charging or discharging, respectively. Spectra are stacked with an offset of 40 Ω in the −*Im*(Z) direction. Reproduced with permission from [203]. Copyright 2017 American Chemical Society.

Dietrich et al. [229] analyzed the crystal structure of LPS electrolytes using synchrotron XRD, Raman spectroscopy, NMR, and conductivity studies and Koerver et al. [203] investigated the fabrication of the Li-In|b-LPS|NMC811|b-LPS ASSB (Figure 10a–e). They highlighted the importance of the interfacial reactivity, cathode/electrolyte interphase (CEI) formation, and electro-chemo-mechanical processes of the SSB active materials. The CEI formation, which mainly occurred during the first cycle, was monitored using in situ impedance spectroscopy, X-ray photoemission spectroscopy (XPS), and SEM imaging. The initial irreversible capacity loss corresponding to a decomposition of the β-Li_3_PS_4_ solid electrolyte is due to an additional resistance (Figure 10a,b). Impedance spectra during (Figure 10d) charge and (Figure 10e) discharge periods were conducted after 1 h of charging or discharging, respectively [203]. The XPS data suggested that the largest passivating layer fraction was formed during the first charge and the layer continued to grow slowly upon further cycling, which led to the slow capacity fading of the cell during cycling. Furthermore, based on these observations, it was concluded that the capacity loss during the first cycle was due to the changes in the chemical composition at the solid electrolyte/electrode interface (oxidation) and the contraction of the NMC particles during delithiation (charging). Moreover, it was proposed that protecting the surface of the cathode using different metal oxide coatings could help to improve the capacity fading and irreversible capacity loss of the cell. Different metal oxides have been used for this purpose, and LiNbO_3_ has been one of the most promising coating materials for the NMC cathode.

**Figure 11:**
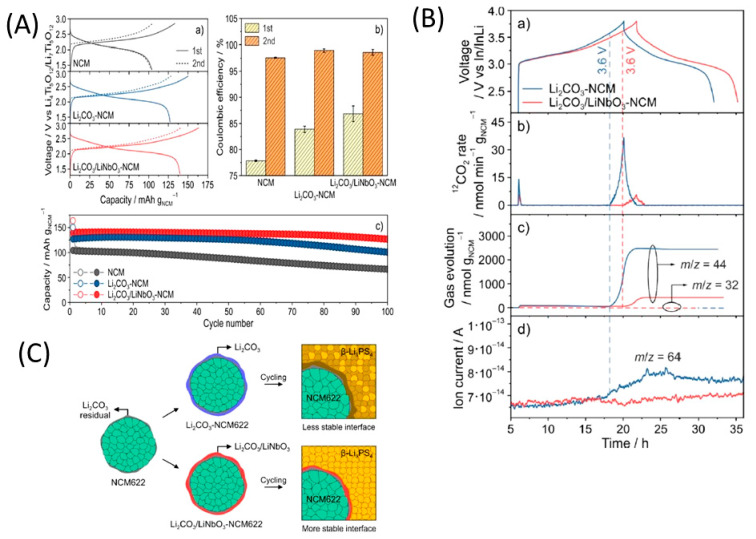
(**A**) (**a**) The 1st and 2nd cycle voltage profiles, (**b**) corresponding Coulombic efficiencies, and (**c**) cycling performance at a rate of C/10 and 25 °C of solid-state batteries (SSBs) using bare (gray), Li_2_CO_3_-coated (blue), and Li_2_CO_3_/LiNbO_3_-coated NMC622 (red) cathodes. In (**b**), the error bars indicate standard deviations. (**B**) (**a**) The 1st cycle voltage profile at a rate of C/20 and 45 °C of SSBs using Li_2_CO_3_-coated (blue) and Li_2_CO_3_/LiNbO_3_-coated NMC622 (red) cathodes. (**b**) The CO_2_ mass signals (*m*/*z* = 44) and (**c**) cumulative amounts. (**d**) Time-resolved ion current for the evolution of SO_2_ (*m*/*z* = 64). (**C**) Illustration of different interfacial reactivities of the Li_2_CO_3_-coated (indicated by the oxidation of the solid electrolyte in dark brown) or Li_2_CO_3_/LiNbO_3_-coated NMC622 cathodes of β-LPS–based SSBs. Reasonably stable solid electrolyte/cathode active material interfaces were achieved only for the Li_2_CO_3_/LiNbO_3_ hybrid coating. Reproduced with permission from [204]. Copyright 2019 American Chemical Society.

In 2019, Kim et al. [204] studied the influence of the hybrid Li_2_CO_3_/LiNbO_3_ coating on the surface of NMC622 cathode in solid-state cell using β-LPS as SSE. They characterized the surface coating well using transmission electron microscopy (TEM), energy-dispersive X-ray spectroscopy, high-angle annular dark-field scanning transmission electron microscopy, electron energy loss spectroscopy, inductively coupled plasma optical emission spectroscopy, XPS, differential electrochemical mass spectroscopy (DEMS), and infrared and impedance spectroscopy. The Li_2_CO_3_-LiNbO_3_–coated NMC SSB presented improved capacity and cycling stability, and it delivered the initial charge–discharge capacities of 157 and 136 mAh g^−1^, respectively, and exhibited a capacity retention of 91% up to 100 cycles when cycled at a current rate of 0.1C. The improved cycling stability of the SSB was attributed to its low interfacial resistance of approximately 25 Ω at the end of 100 cycles compared with those of the SSBs with bare NMC (900 Ω) and Li_2_CO_3_-coated NMC (60 Ω) cathodes. The interfacial reactions were further studied using XPS, and the results revealed that S oxidation occurred during cycling irrespective of the surface modification of the NMC cathode; however, the decrease in thickness of the interfacial layer was observed from the bare NMC to the Li_2_CO_3_-coated NMC and Li_2_CO_3_/LiNbO_3_-coated NMC cathodes. Furthermore, the presence of P*_x_*O*_y_* species was noted and was ascribed to the reaction of the electrolytes with the gases evolved at the cathode during electrochemical cycling. The results of the DEMS analysis of the coated samples in charged state at 3.6 V vs. Li-In are presented in Figure 11A–C [204]. The CO_2_ evolution of the Li_2_CO_3_-coated NMC cathode exceeded that of the Li_2_CO_3_/LiNbO_3_-coated NMC cathode. Furthermore, because the mass ratio between SO_2_ and the Li_2_CO_3_-coated NMC cathode was approximately *m*/*z* = 64, it was demonstrated that the formed O_2_ species reacted with the electrolyte to produce corrosive SO_2_ gas. Based on this study, it was concluded that the decomposition of the surface carbonate resulted in the formation of highly reactive ^1^O_2_ species, which further reacted with β-LPS to form SO_2_. Subsequent SEM studies indicated that the decomposition of the solid electrolyte was negligible when it was paired with the Li_2_CO_3_/LiNbO_3_-coated NMC cathode. Lastly, it was concluded that the interfacial mechanism of solid electrolyte decomposition strongly depended on the coating technique and surface chemistry, and the results are illustrated in Figure 11C.

Neumann et al. [230] further studied the LPS electrolyte/NMC622 microstructure and interface topology using X-ray tomography and 3D microstructure–resolved simulations and combined impedance technique and electrochemical studies that revealed the low electronic conductivity of in the fully lithiated NMC622 material (σ = 1.42 × 10^−4^ S cm^−1^ for Li = 0.4 down to 1.6 × 10^−6^ S cm^−1^ for Li = 1). This inherent restriction prevents a high cathode utilization, and also geometrical properties and morphological changes of the microstructure interact with internal and external interfaces, which significantly affect the capacity retention at higher current rates. Nakamura et al. [231] further improved the coating technology of electrodes and electrolytes and reported uniformly coating LPS on an NMC111 cathode using the dry-coating technique. This technique is advantageous owing to its amenability for large-scale preparation and good dispersion of the cathode and electrolyte. Recently Shi et al. [232] used a Li_2_O–ZrO_2_ (LZO)-coated NMC cathode and an amorphous 75Li_2_S–25P_2_S_5_ (LPS) solid electrolyte. They reported that a high cathode utilization was obtained by reducing the solid electrolyte particle size and increasing the active cathode material particle size, over 50 vol.%. This concept was confirmed computationally using ab initio MD and a model related to the ionic percolation in the cathode composite. Ito et al. [233] adopted a sulfide-based electrolyte, Li_2_S–P_2_S_5_ (80:20 mol%) and LZO-coated LiNi_0.8_Co_0.15_Al_0.05_O_2_ (NCA) cathode to fabricate ASSBs, which retained 80% of their initial capacity after 100 cycles. Camacho-Forero et al. [184], Kim et al. [234], and Pan et al. [235] performed additional computational studies on β-LPS. Smith and Siegel [236] showed that the “paddlewheel” mechanism combines the Li ion migration with quasi-permanent reorientations of PS_4_^3-^ anions in Li_2_S-P_2_S_5_ glasses.

**Figure 12:**
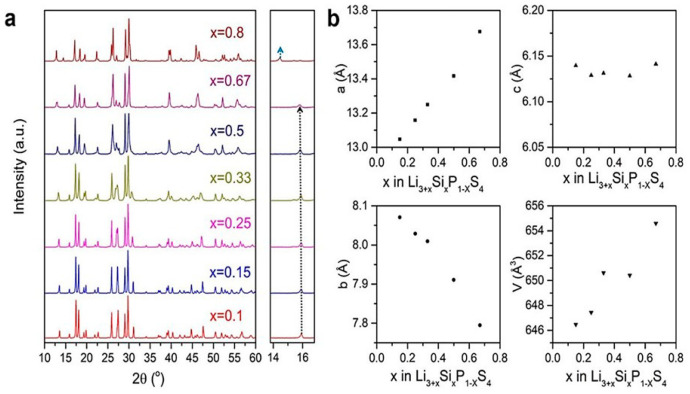
(**a**) Selected X-ray diffraction patterns of as-synthesized Li_3+*x*_[Si*_x_*P_1−*x*_]S_4_ (*x* = 0.1, 0.15, 0.25, 0.33, 0.5 0.67, 0.8); the black arrow indicates the (101) reflection for orthorhombic Li_3+*x*_[Si*_x_*P_1−*x*_]S_4_ (*x* = 0.1, 0.15, 0.25, 0.33, 0.5, 0.67) and the blue arrow indicates the (100) reflection for monoclinic Li_3.8_[Si_0.8_P_0.2_]S_4_. (**b**) Changes in lattice parameters and unit cell volume of orthorhombic Li_3+*x*_[Si*_x_*P_1–*x*_]S_4_ phases with the Si content for single crystal structure solutions at 280 K. Reproduced with permission from [222]. Copyright 2019 American Chemical Society.

In 2019, Zhou et al. [222] investigated the ionic conductivity of Li_3+*x*_[Si*_x_*P_1−*x*_]S_4_ (0.15 < *x* < 0.33) prepared by solid solution methods using a mixture of Li_2_S, P_2_S_5_, Si, and S; 5 wt.% excess S was added to the mixture to fully oxidize Si. First, the powder was pelletized, then it was placed in a glassy-carbon crucible in a sealed quartz tube under vacuum. The sample was heated to 750 °C, slowly cooled to 725 °C for 18 h, and then cooled to room temperature at the rate of 5 °C min^−1^. The material was further characterized using XRD, neutron diffraction, NMR, bond valence calculations, and conductivity measurements. Crystal structure studies revealed that Li_3+*x*_[Si*_x_*P_1−*x*_]S_4_ was isostructural with β-LPS (Figure 9); however, slight differences existed in the values of the lattice parameters *a* and *c*. Li_3+*x*_[Si*_x_*P_1−*x*_]S_4_ presented orthorhombic structure with *Pnma* space group; *a* = 13.158(2) Å, *b* = 8.029(0) Å, and *c* = 6.129(1) Å (Figure 12a,b) [222]. The XRD patterns of LPS revealed that the values of the lattice parameters *a* and *c* monotonically increased and decreased, respectively, when the LPS lattice was doped with Si (Figure 12), which confirmed the formation of solid solutions. ^29^Si and ^31^P magic angle spinning NMR studies on Li_3+*x*_[Si*_x_*P_1−*x*_]S_4_ (*x* = 0.25, 0.33, 0.67) revealed the presence of peaks at the chemical shifts, of ∼5 and ∼86.5 ppm, which corresponded to the SiS_4_^4–^ and PS_4_^3–^ moieties, respectively.

**Figure 13:**
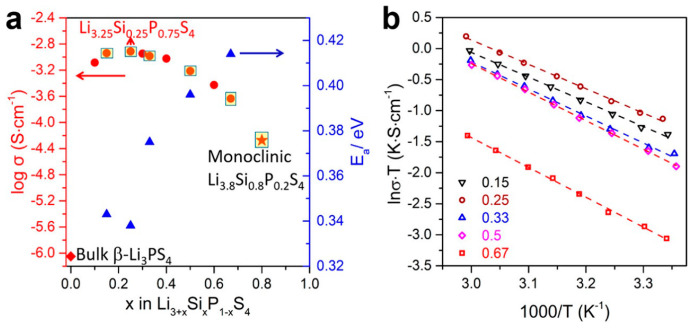
(**a**) Ionic conductivity (red dots) at room temperature and activation energy (*E*_a_) (blue triangles) of Li_3+*x*_[Si*_x_*P_1−*x*_]S_4_ as function of the Si content (*x*); the squares around the data points indicate the compositions for which the structure has been solved using single crystal diffraction. (**b**) Arrhenius plots of Li_3+*x*_[Si*_x_*P_1−*x*_]S_4_ (*x* = 0.15, 0.25, 0.33, 0.5, 0.67). Reproduced with permission from [222]. Copyright 2019 American Chemical Society.

Li_3.25_Si_0.25_P_0.75_S_4_ presented the highest ionic conductivity of 1.22 mS cm^−1^ at room temperature of all Li_3+*x*_[Si*_x_*P_1−*x*_]S_4_ (*x* = 0.1, 0.15, 0.25, 0.33, 0.5 0.67, 0.8) solid solutions (Figure 13a,b); moreover, its ionic conductivity was three orders of magnitude higher than that of bulk β-LPS [222]. Using soft bond valence calculations, Zhou et al. [222] predicted that Li_3.25_[Si_0.25_P_0.75_]S_4_ presented a 3D Li^+^ ion diffusion pathway and lower overall *E*_a_ (~0.2 eV) than β-LPS and suggested that the Li^+^ ion diffusion occurred both along the *b*-axis and in the (*a*,*c*) plane. Owing to its flexible and ductile nature, the Li_3+*x*_[Si*_x_*P_1−*x*_]S_4_ electrolyte could be more easily processed and densified than sulfide and oxide electrolytes. Moreover, owing to its synthesis temperature being similar to that of the cathode, this electrolyte could be useful for the preparation of ASSB oxide/sulfide composite electrolytes.

**Figure 14:**
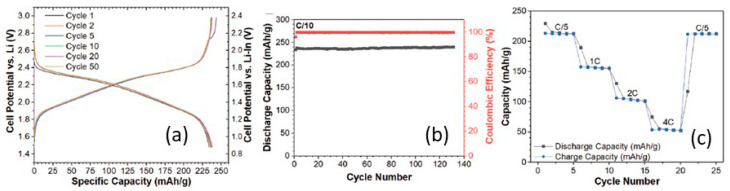
(**a**) Charge–discharge curves of Li-In|LIBOSS|TiS_2_ all-solid-state battery cycled at C/10 at 25 °C and (**b**) cycling performance of the battery cycled at C/10 at 25 °C. C rate capability study: discharge capacity (black line), coulombic efficiency (red line). (**c**) cycling data at 60 °C at a rate of 1C. Reproduced with permission from [237]. Copyright 2020 Wiley.

Kaup et al. [237] studied 30Li_2_S–25B_2_S_3_–45LiI–*x*SiO_2_ (Li_1.05_B_0.5_Si*_x_*O_2*x*_S_1.05_I_0.45_) (0 ≤ *x* ≤ 1) quaternary superionic Li oxythioborate glasses. The prepared compositions presented negligible H_2_S evolution on pellets upon exposure to ambient air and a stable capacity of 230 mAh g^−1^ up to 230 cycles, at a rate of 0.1C when paired with a TiS_2_ intercalation cathode (Figure 14a–c). Such a cell showed an average voltage of ~2.2 V vs. Li much lower than that of pristine layered NMC cathode [2].

### 3.3. Li_7_P_3_S_11_

Li_7_P_3_S_11_ has been widely investigated in the form of either glass or ceramic [210,238,239,240,241,242,243,244,245,246,247,248,249,250,251,252,253,254,255,256,257,258,259,260]. Minami et al^.^ [238,239,240,241,242,243], Yamane et al^.^ [244], Hayashi et al. [245,246,247], and Kowada et al. [248] reported the synthesis of Li_7_P_3_S_11_ from the (100−*x*)Li_2_S–*x*P_2_S_5_ (*x* = 30) glass composite and evaluated the effects of the ball milling time and crystallization temperature on the conductivity (~0.2 mS cm^−1^) and electrolytic stability for ASSBs. Ujiie et al. [249,250] further analyzed the compositions (100−*y*)(0.7Li_2_S·0.3P_2_S_5_)·*y*Li*X*, i.e., 0 ≤ *y* ≤ 20 mol%, by substitution of Li*X* (*X* = F, Cl, Br) for Li_7_P_3_S_11_. They noted that the crystallinity of the Li*X*-substituted Li_7_P_3_S_11_ decreased with increasing the Li*X* content and the highest conductivity of 6.5 × 10^−6^ S cm^−1^ was achieved for the LiBr-substituted material.

Onodera et al. [251] analyzed the origin of the ionic conductivity and crystal structure of the Li_7_P_3_S_11_ electrolyte using neutron diffraction and XRD and performed early computational studies to investigate the Li defects in this electrolyte by Xiong et al. [252] and combined computational and experimental studies by Chu et al. [253]. Furthermore, Mori et al. [254], Wohlmuth et al. [255], Busche et al. [256], and Wenzel et al. [257] performed solid-state NMR interface studies. Liu et al. [258] carried out XPS studies on the formation of the solid electrolyte interphase between Li_7_P_3_S_11_ and Li metal. Wang et al. [259] reported the wet chemical synthesis of Li_7_P_3_S_11_ and noted that its conductivity was lower than that of the Li_7_P_3_S_11_ synthesized using the solid-state method. Jung et al. [210] fabricated Li_2_OHBr-substituted Li_7_P_3_S_11_ electrolytes, i.e., (100−*x*)Li_7_P_3_S_11_–*x*Li_2_OHBr (*x* = 0, 2, 5, 10, 20, 30, 40, 50), to improve the electrolyte stability. The conductivity of 90Li_7_P_3_S_11_–10Li_2_OHBr (4.4 × 10^−4^ S cm^−1^ at room temperature) was the highest value of all prepared samples; moreover, the reversible capacity of 90Li_7_P_3_S_11_–10Li_2_OHBr was 135 mAh g^−1^. Preefer et al. [260] reported a rapid microwave assisted synthesis of Li_7_P_3_S_11_ material, which was characterized by XRD, XPS, and Raman techniques and showed a comparable conductivity of the material prepared by melt quenched method.

### 3.4. Li_7_P_2_S_8_I

Rangasamy et al. [261] reported that the room-temperature conductivity and *E_a_* of Li_7_P_2_S_8_I were 6.3 × 10^−4^ S cm^−1^ and 0.31 eV, respectively (Table 1). Later, Kang and Han [262] analyzed the crystal structure and transport behaviors of solid electrolytes using DFT calculations and ab initio MD simulations. They reported that the orthorhombic lattice (*Pnma* space group) parameter values were *a* = 9.46 Å, *b* = 7.81 Å, and *c* = 11.74 Å, and β = 75.17°, and these values were different than those previously reported. Furthermore, computational studies demonstrated that the Li^+^ ions preferred to diffuse along the *c*-axis over the *a*- or *b*-axis; moreover, the conductivity at room temperature was 0.3 mS cm^−1^, which is in good agreement with the experimentally reported value. Rangasamy et al. [261] reported a conductivity value of 6.3 × 10^−4^ S cm^−1^ (Table 1). Rao et al^.^ [188] performed the crystal structure refinements on the Li_x_(PS_4_)_y_*X*_z_ (*X* = Cl, Br, I) system and reported that it contained a mixture of two phases: 13% LiI and 87% tetragonal Li_4_(PS_4_)I, whereas the LPS:LiI (2:1) sample comprised three phases: 72.5% Li_4_(PS_4_)I, 15% Li_4_P_2_S_6_, and 12.5% unreacted LPS. Wang et al. [263] fabricated ultrathin Li-thiophosphate solid electrolyte membrane β-Li_3_PS_4_ stable with metallic lithium anode up to 5 V.

**Figure 15:**
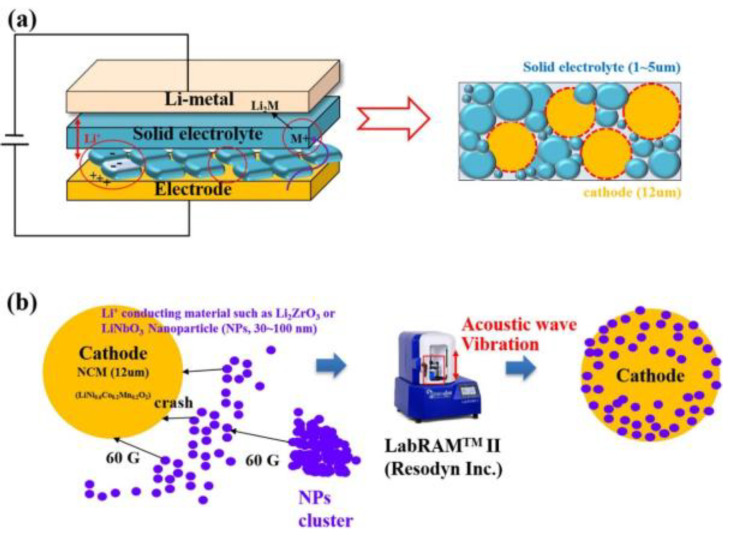
Schematic diagrams of (**a**) critical drawbacks of all-solid-state Li batteries and (**b**) resonant acoustic dry coating technique. Reproduced with permission from [265]. Copyright 2020 Elsevier.

Choi et al. [264] studied the cell with a composite cathode/electrolyte LNO-NMC622/Li_7_P_2_S_8_I/conducting carbon (75:23:2) pressed at 30 MPa and Li metal anode. When the pellet-type test cell was tested at a current rate of C/50 and the slurry-type cell was cycled at 55 °C and current rate of C/50, they delivered the initial discharge capacities of ∼150 and ~120 mAh g^−1^, respectively. Kim et al. [265] analyzed a cell with 1–3 wt.% LiNbO_3_-and-LiZr_2_O_3_-coated (LiNi_0.6_Mn_0.2_Co_0.2_)O_2_ and Li_7_P_2_S_8_I as the cathode and electrolyte, respectively, using the resonant acoustic dry coating technique (Figure 15a,b).

**Figure 16:**
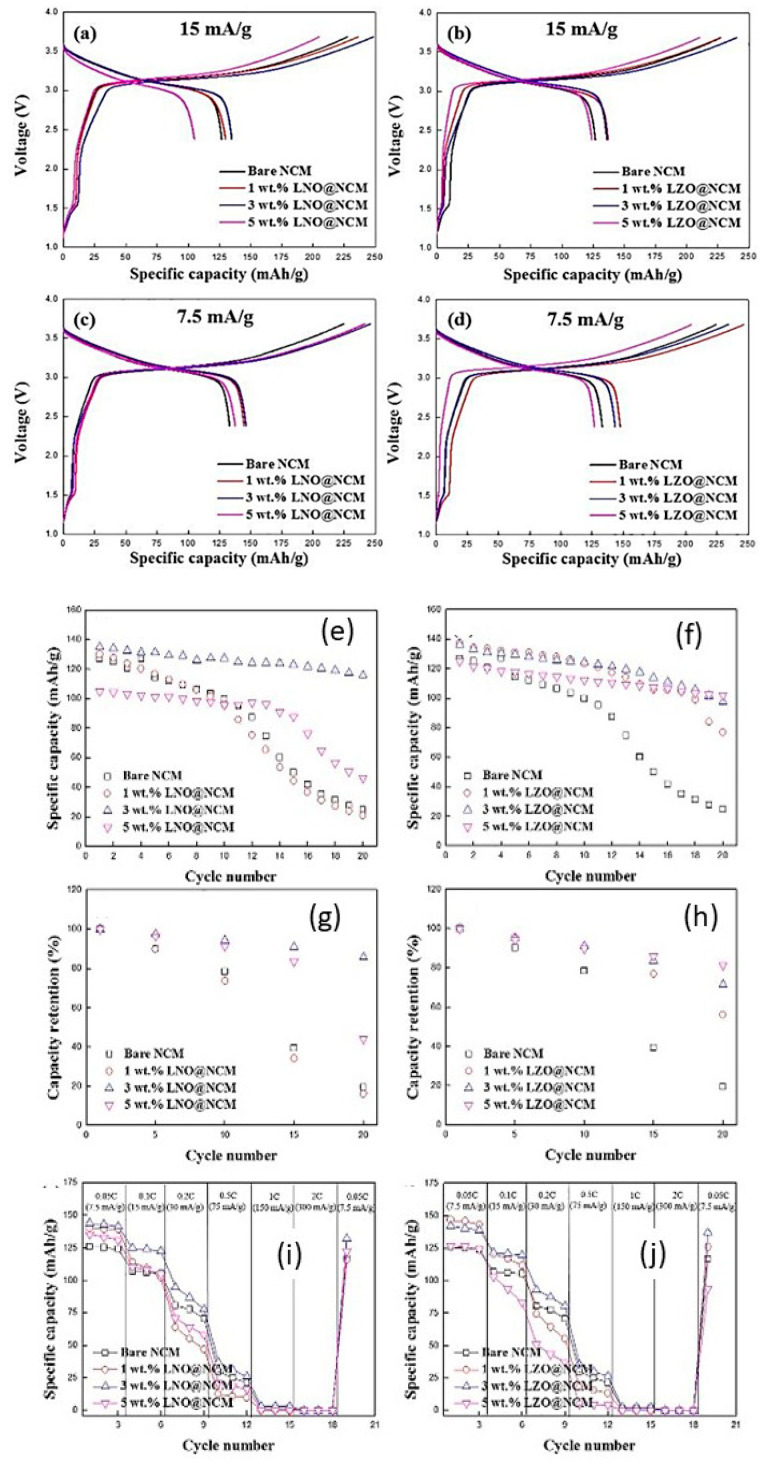
Charge–discharge curves of (**a**) LiNbO_3_ (LNO)-coated LiNi_0.8_Co_0.1_Mn_0.1_O_2_ (NMC), (**b**) Li_2_ZrO_3_-coated NMC at a current density of 0.1C (current rate of 15 mA g^−1^), and (**c**) LNO-coated NMC, (**d**) Li_2_ZrO_3_-coated NMC at a current density of 0.05C (current rate of 7.5 mA g^−1^) obtained using the Li_0.5_In|Li_7_P_2_S_8_I|LiNi_0.6_Co_0.2_Mn_0.2_O_2_ cell in the range of 3.68–2.38 V. Cycle performances of: (**e**) LNO-coated NMC, (**f**) Li_2_ZrO_3_-coated NMC at a current density of 0.1C (current rate of 15 mA g^−1^). Cycle retentions of (**g**) LNO-coated NMC and (**h**) Li_2_ZrO_3_-coated NMC. C-rate performances of (**i**) LNO-coated NMC and (**j**) Li_2_ZrO_3_-coated NMC at different current densities of 0.05, 0.1, 0.2, 0.5, 1, 2, and 0.05C obtained using the Li_0.5_In|Li_7_P_2_S_8_I|LiNi_0.6_Co_0.2_Mn_0.2_O_2_ cell in the range of 3.68–2.38 V. Reproduced with permission from [265]. Copyright 2020 Elsevier.

A zirconia container was accelerated using acoustic waves and vibration energy of up to 60 G; the LiNbO_3_ cluster was broken into nanoparticles, and the particles were deposited on the surface of an NMC cathode. Subsequently, the aforementioned electrolyte and cathode were paired with a Li_0.5_In alloy anode, which was manufactured by mixing Li and In powders (1:2 mole ratio), to fabricate an ASSB. They improved high capacity with 3 wt.% coated NMC up to 20 cycles (Figure 16a–j [265].

### 3.5. Li_11−x_M_2−x_P_1+x_S_12_ (M = Ge, Sn, Si) (LGPS)-Type Structures

**Figure 17:**
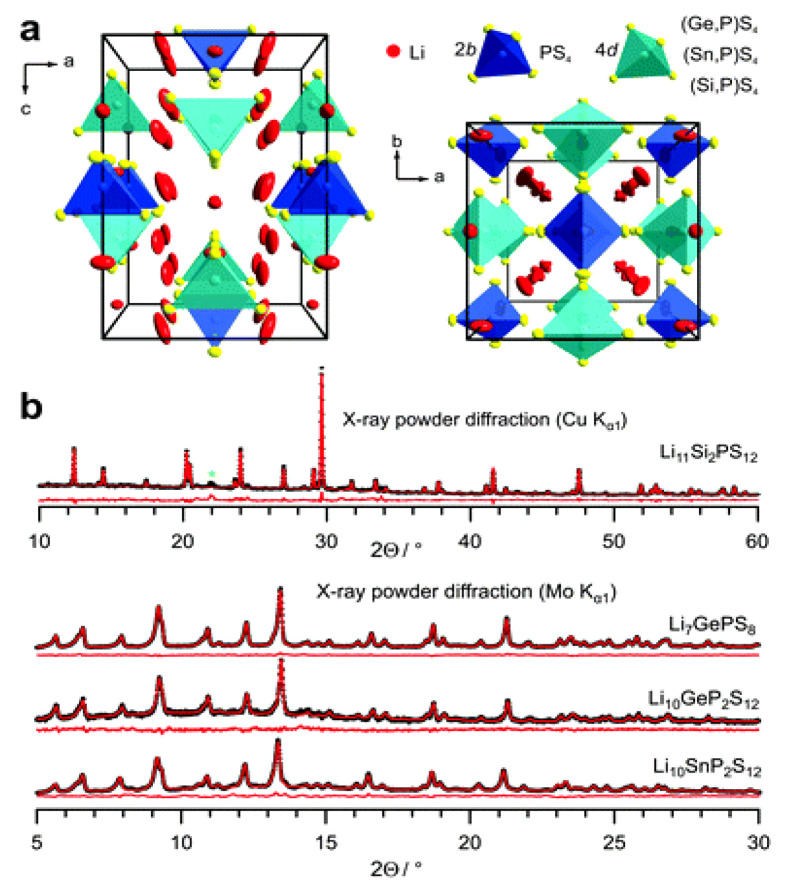
(**a**) Crystal structure of tetragonal Li_10_GeP_2_S_12_ (LGPS) obtained using single-crystal X-ray diffraction. (**b**) X-ray powder diffraction patterns and Rietveld refinements of Li_11_Si_2_PS_12_ and Li_10_SnP_2_S_12_ compared with those previously reported for Li_10_GeP_2_S_12_ and Li_7_GePS_8_. The side phase was marked with a green asterisk. Reproduced with permission from [270]. Copyright 2014 Royal Society of Chemistry.

In 2011, Kamaya et al. [266] synthesized the Li_10_GeP_2_S_12_ (LGPS) solid electrolyte and reported a conductivity of 9 × 10^-3^ S cm^−1^ (Table 1) and electrochemical properties of a LiCoO_2_-LGPS|LGPS|In cell. Moreover, other researchers have extensively analyzed this system [267,268,269,270,271,272,273,274,275,276,277]. LGPS presented tetragonal crystal structure with the lattice parameters *a* = 8.708 Å and *c* = 12.605 Å and consisted of negatively charged PS_4_^3−^ and GeS_4_^4−^ tetrahedra surrounded by (mobile) Li^+^ ions for charge compensation as shown in Figure 17a, and X-ray powder diffraction patterns and Rietveld refinements of Li_11_Si_2_PS_12_ and Li_10_SnP_2_S_12_ are compared with those previously reported for Li_10_GeP_2_S_12_ and Li_7_GePS_8_ in Figure 17b [270]. The tetrahedrally coordinated Li1 and Li3 sites generated channels for the facile Li^+^ ion diffusion along the *c*-axis and the octahedrally coordinated Li2 positions between those channels were assumed to be inactive for diffusion [268].

Adams et al. [267] performed bond valence calculations and MD simulations on LGPS, and Kuhn et al. [268,269] analyzed the structure dynamics of LGPS using various techniques, such as XRD, electron diffraction, NMR, and impedance studies. They confirmed the previously reported high ionic conductivity of LGPS of ∼10^−2^ S cm^−1^ and *E*_a_ of ~0.22 eV (Table 1). Furthermore, Kuhn et al. [270] utilized the high-pressure synthesis method used to fabricate Li_11_Si_2_PS_12_ for obtaining other Li_11−*x*_*M*_2−*x*_P_1+*x*_S_12_ (*M* = Ge, Sn) LGPS-type structures, such as Li_10_GeP_2_S_12_, Li_7_GePS_8_, and Li_10_SnP_2_S_12_, and reported that the Li^+^ ion diffusion coefficients of Li_11_Si_2_PS_12_, Li_10_Ge_2_P_2_S_12_, and Li_10_Sn_2_P_2_S_12_ were 3.5 × 10^−12^, 2.2 × 10^−12^, and 2.8 × 10^−12^ cm^2^ s^−1^, respectively, which correspond to Li jump rate of 1.5 × 10^4^ s^−1^ at 125 K, 1.4 × 10^4^ s^−1^ at 135 K and 145 K obtained from NMR studies. Weber et al. [137] also studied the structure and 3D diffusion pathways of LGPS-type structures. Using first principles computation methods, Han et al. [271] calculated the intrinsic electrochemical stability window of Li_10_Ge_2_P_2_S_12_, addressing the challenging problems of the interfacial stability and internal resistance. Ong et al. [272] and Mo et al. [273] performed first-principles calculations on Li_10±1_*M*P_2_*X*_12_ (*M* = Ge, Si, Sn, Al, P, and *X* = O, S, Se) and analyzed in detail the phase stability, electrochemical stability, and Li^+^ ion conductivity of the aforementioned superionic conductors. Their computational studies were very useful for researchers studying sulfide electrolytes and led to better understanding of the stability of the electrolyte and electrode materials. In addition, Hu et al. [274] and Du et al. [275] performed computational analysis on LGPS-type structures, Binninger et al. [276] investigated the electrochemical stability window of LGPS-type structures, and Gorai et al. [277] performed electronic structure and defect chemistry calculations for LGPS-type structures.

**Figure 18:**
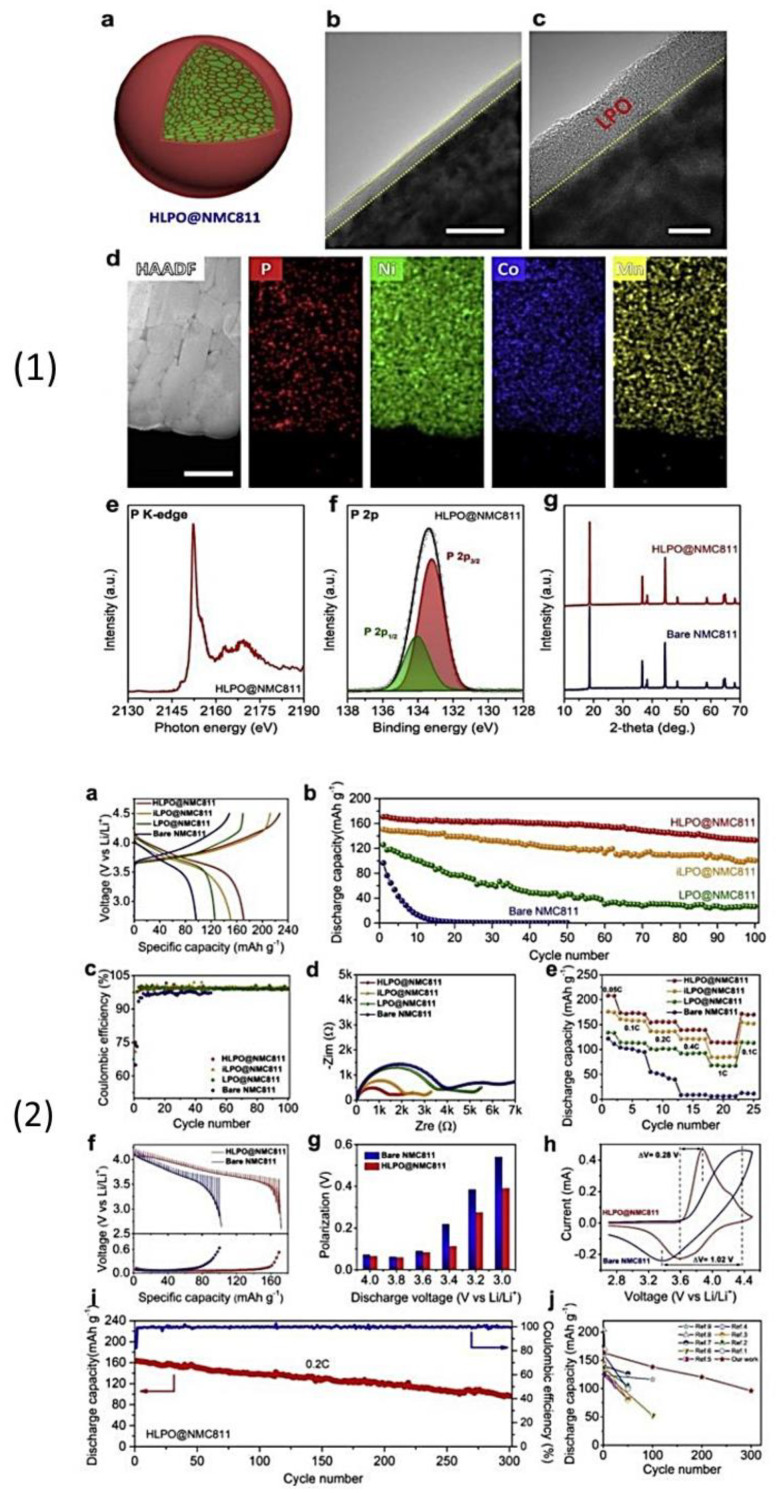
(**1**) (**a**) Schematic illustration of the detailed structure of HLPO@NMC811, (**b**–**c**) HR-TEM images of the secondary LPO coating layer on the HLPO@NMC811 surface at different magnifications, (**d**) EDX mappings of the cross-sectional HLPO@NMC811, (**e**–**f**) P K-edge XANES and P 2p XPS spectra of HLPO@NMC811, (**g**) XRD patterns of the bare NMC811 and HLPO@NMC811. Scale bars in (**b**), (**c**), and (**d**) are 20 nm, 5 nm, and 500 nm, respectively. (**2**) Effectiveness of various Li_3_PO_4_ modifications for the performance of all-solid-state Li-ion batteries. (**a**) First cycle charge–discharge curves, (**b**) cycling stabilities at the current rate of 0.1C, (**c**) corresponding Coulombic efficiencies, (**d**) electrochemical impedance spectroscopy plots after 100 cycles, and (**e**) rate capabilities of four types of NMC811 cathodes. (**f**) Galvanostatic intermittent titration technique curves during the discharge process (top) and corresponding polarization plots (bottom), (**g**) polarization plots at selected discharge voltages, (**h**) cyclic voltammetry profiles at the first cycle of the optimal HLPO@NMC811 and bare NMC811 cathodes. (**i**) Long-term cycling stability of HLPO@NMC811 cathode at 0.2C. (**j**) Cycling performance of the Ni-rich Li(Ni_x_Mn_y_Co_z_)O_2_ cathodes in sulfide-based all-solid-state Li-ion batteries. Reproduced with permission from [280]. Copyright 2020 Elsevier.

Li et al. [278] fabricated ASSBs and performed interfacial studies on LiNi_0.85−*x*_Co_0.15_Al*_x_*O_2_ (*x* = 0.05, 0.15, 0.25) and Li_10_GeP_2_S_12_ using in situ and ex situ Raman and impedance spectroscopy. They noted that the capacity and capacity retention of the Al-doped sample (*x* = 0.15) were higher than those of the undoped sample; moreover, less reactions occurred at the electrode/electrolyte interface of the Al-doped sample than at the interface of the undoped one. Mei et al. [279] measured the ionic conductivity measurements of poly(ethylene oxide) (PEO)_18_–LiClO_4_–*x* wt.% LGPS. Deng et al. [280] fabricated hierarchical LPO-coated NMC 811 (HLPO@NMC811) using the atomic layer deposition (ALD) technique. A battery was fabricated using a 10 mm diameter commercial LGPS disk subjected to 2 ton (~250 MPa) of pressure as the electrolyte. Then, a mixture of LPO-coated NMC811 and LGPS powders (70:30 w/w) was subjected to 3 ton (~380 MPa) of pressure. In addition, the In/Li foil used as the anode was placed on the opposite side of the LGPS pellets and the ensemble was subjected to 0.5 ton (~65 MPa) of pressure. Stainless-steel rods were used as the current collectors. No additional pressure was applied during the electrochemical cycling of the battery. The battery delivered a specific capacity of 170 mAh g^−1^ at a current rate of 0.1C, a capacity retention of 77.9%, and retained a capacity of 96 mAh g^−1^ after 300 cycles (Figure 18(1),(2)), when the LPO-coated NMC cathode was optimized; the charge–discharge experiments were performed in the potential range of 2.7–4.5 V vs. Li^+^/Li at room temperature. The reported improvement in cycling stability was further confirmed using XPS and X-ray absorption near edge structure studies, which demonstrated that the formation of SO_x_ was suppressed for the LPO-coated NMC811 sample; however, more side reactions that generated SO_x_ were noted for the bare NMC/LGPS electrodes. Zhang et al. [281] studied the chemical stability of LGPS and improved the Li interface by coating Li with a protective LiH_2_PO_4_ layer. The ASSB fabricated using LNO-coated LCO presented the reversible capacities of 131 and 114 mAh g^−1^ for the 1st and 500th cycles, respectively, at a current rate of 0.1C; moreover, the capacity retention of the ASSB was 86.7%. Zheng et al. [282] and Philip et al. [283] studied LGPS/PEO composites and Paulus et al. [284] conducted NMR experiments that demonstrated the relaxation coupling of the ^7^Li (*I* = 3/2) longitudinal magnetization order in the LGPS electrolyte. Electrochemical performance of sulfide-based electrolytes for all-solid-state batteries are listed in Table 2.

Zhang et al. [285] prepared LGPS via planetary ball milling followed by heating. In addition, Kim et al. [286] conducted studies on ionic liquids and LGPS composites. Few attempts were made to improve the structural stability of the LGPS lattice via Ba, Al, or Si doping. Sun et al. [287] reported that the ionic conductivity of Ba-doped LGPS (Li_9.4_Ba_0.3_GeP_2_S_12_) was 7.04 × 10^−4^ S cm^−1^ at 25 °C. Moreover, they ascribed the improvement in the structural stability of the LGPS lattice to the strong Coulombic interactions between the Ba^2+^ and Li^+^ ions. Although LGPS presented reasonably good conductivity, the high cost of Ge and reaction with Li to form Li*_x_*Ge alloys limit the use of LGPS for large-scale applications for SSBs. 

**Figure 19:**
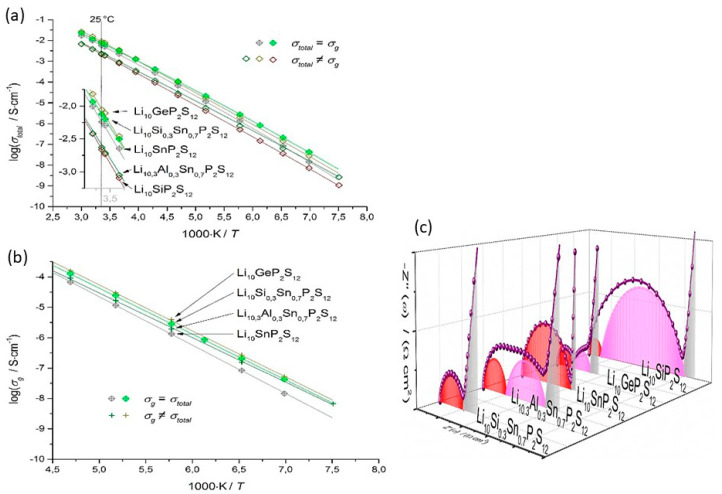
(**a**) Arrhenius plot of the total conductivities and quadratic best fit curves. The magnified image illustrates the temperature range of 0–40 °C. (**b**) Arrhenius plot of the grain conductivities and corresponding linear best fits in the temperature range of −140 to −60 °C. (**c**) Nyquist plots of electrolyte compositions. Reproduced with permission from [292]. Copyright 2016 Elsevier.

Further efforts have been devoted to the search for new inexpensive electrolytes with good electrochemical stability. Whiteley et al. [288] used Li_2_S–SiS_2_–P_2_S_5_ to prepare the Li_10_SiP_2_S_12_ (LSiPS) electrolyte via cold pressing. The obtained electrolyte was isostructural with LGPS and delivered a room-temperature conductivity of 2.3 × 10^−3^ S cm^−1^, and this value was close to those reported by Bron et al. [292] (Table 1). Moreover, LSiPS presented good stability when paired with Li metal and good cycling voltage window when paired with a cathode material. The conductivity of LSiPS could be further improved via hot pressing, and therefore, this could be a promising ASSB electrolyte. Fitzhugh et al. [289] performed computational studies on Li_10_SiP_2_S_12_ paired with a coated cathode. Kim and Martin [290] analyzed the effect of O-doping on the crystal structure of Li_10_SiP_2_S_12−*x*_O*_x_* (LSiPSO) (0 ≤ *x* ≤ 1.75) using XRD, Raman, Fourier transform infrared, and solid-state NMR spectroscopies, and ionic conductivity measurements. They noted that at low oxygen doping levels (*x* = 0.7 and 0.9), the structure of the LSiPSO phases (Li_10.35_P_1.65_Si_1.35_S_12_ with lattice parameters *a* = 8.66 Å and *c* = 12.52 Å) became more homogeneous with minor amounts of β-LPS impurity, while, at high oxygen doping levels, the structure of the LSiPSO samples resembled to that of LGPS. For *x* = 0, the compound is a mixture of LSiPSO and β-LPS impurity phase. Conductivity measurements revealed that the Li ionic conductivity increased with the decrease in the amount of β-LPS phase, and the highest Li ionic conductivity of 3.1 × 10^−3^ S cm^−1^ at 25 °C was achieved for *x* = 0.7 and 1.6 × 10^−3^ S cm^−1^ for *x* = 0. The ionic conductivity decreased when *x* ≥ 0.9 owing to the degradation of the crystalline LGPS-like phase and generation of the O-rich LPO phase. Harm et al. [291] reported a new Li_7_SiPS_8_ electrolyte, which is isostructural with the LGPS electrolyte and presented a tetragonal structure with the *P*42/*nmc* (no. 137) space group and the lattice parameters *a* = 8.690(5) Å and *c* = 12.570(3) Å. The room-temperature conductivity of this electrolyte was up to 2 mS cm^−1^. Bron et al. [292,293] determined the conductivities of Li_10_Si_0.3_Sn_0.7_P_2_S_12_ and other two superionic conductors, viz. Li_10_SnP_2_S_12_ and Li_10_GeP_2_S_12_ (Figure 19a–c).

Li_10_Si_0.3_Sn_0.7_P_2_S_12_ and Li_10_SnP_2_S_12_ presented low grain boundary resistance; moreover, the conductivity of Li_10_Si_0.3_Sn_0.7_P_2_S_12_ was 8 mS cm^−1^ at 25 °C with *E*_a_ of 0.29 eV, which was similar to that of LGPS (Table 1). They complemented the mechanisms using time-resolved impedance studies [293] of solid electrolytes sandwiched between Li foils using two airtight electrode cells. The overall cost of using this electrolyte for large-scale applications was lower than that of using the LGPS electrolyte. Nam et al. [294] performed first-principles density functional theory calculations and ab initio MD simulations on Li_10−x_SnP_2_S_12−x_Cl_x_. Sun et al. [295] further studied Li_10+*δ*_[Sn*_y_*Si_1–*y*_]_1+*δ*_P_2−*δ*_S_12_ solid solutions that were prepared using the solid-state method. Among all analyzed samples, Li_10.35_[Sn_0.27_Si_1.08_]P_1.65_S_12_ presented the highest room-temperature ionic conductivity of 1.1 × 10^−2^ S cm^−1^, and this value was similar to the previously reported ionic conductivity of LGPS.

**Figure 20:**
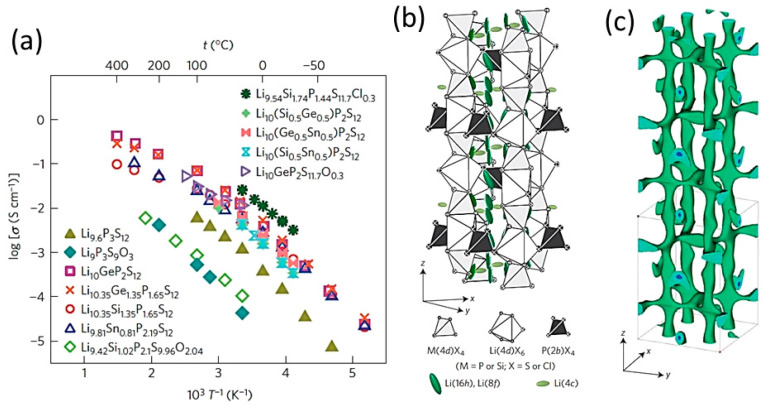
(**a**) Arrhenius conductivity plots of Li_11−_*_x_M*_2−*x*_P_1__+*x*_S_12_ (*M* = Ge, Sn, Si) structures, Li_9.6_P_3_S_12_, and Li_9.54_Si_1.74_P_1.44_S_11.7_Cl_0.3_ electrolytes. (**b**) Crystal structure of Li_9.54_Si_1.74_P_1.44_S_11.7_Cl_0.3_. The thermal ellipsoids were drawn with 50% probability. The framework structure consists of one-dimensional polyhedral chains (edge-sharing M(4*d*)*X*_4_ and Li(4*d*)*X*_6_) connected by P(2*b*)*X*_4_ tetrahedra. Conducting Li is located at the interstitial Li(16*h*), Li(8*f*) and Li(4*c*) sites. (**c**) Nuclear distributions of Li atoms in Li_9.54_Si_1.74_P_1.44_S_11.7_Cl_0.3_ at 25 °C calculated using the maximum entropy method at the iso-surface level of −0.06 fm Å^-3^. Reproduced with permission from [296]. Copyright 2016 Springer.

In 2016, Katto et al. [296] investigated Li_9.54_Si_1.74_P_1.44_S_11.7_Cl_0.3_, a new Li superionic conductor. The excellent conductivity of this material of 2.5 × 10^−2^ S cm^−1^ (Table 1) was twice as high as that of the LGPS electrolyte (Figure 20a–c). This excellent ionic conductivity could be ascribed to the 3D conduction pathway for Li^+^ ions. Later, Bai et al. [297] synthesized Li_9.54_Si_1.74_P_1.44_S_11.7_*X*_0.3_ (*X* = F, Cl, Br, I) and reported that the conductivity of Li_9.54_Si_1.74_P_1.44_S_11.7_I_0.3_ was high as 1.35 mS cm^−1^. Choi et al. [298] reported studies on electronic structures of Li_9.54_Si_1.74_P_1.44_S_11.7_I_0.3_ by atomic simulation.

**Figure 21:**
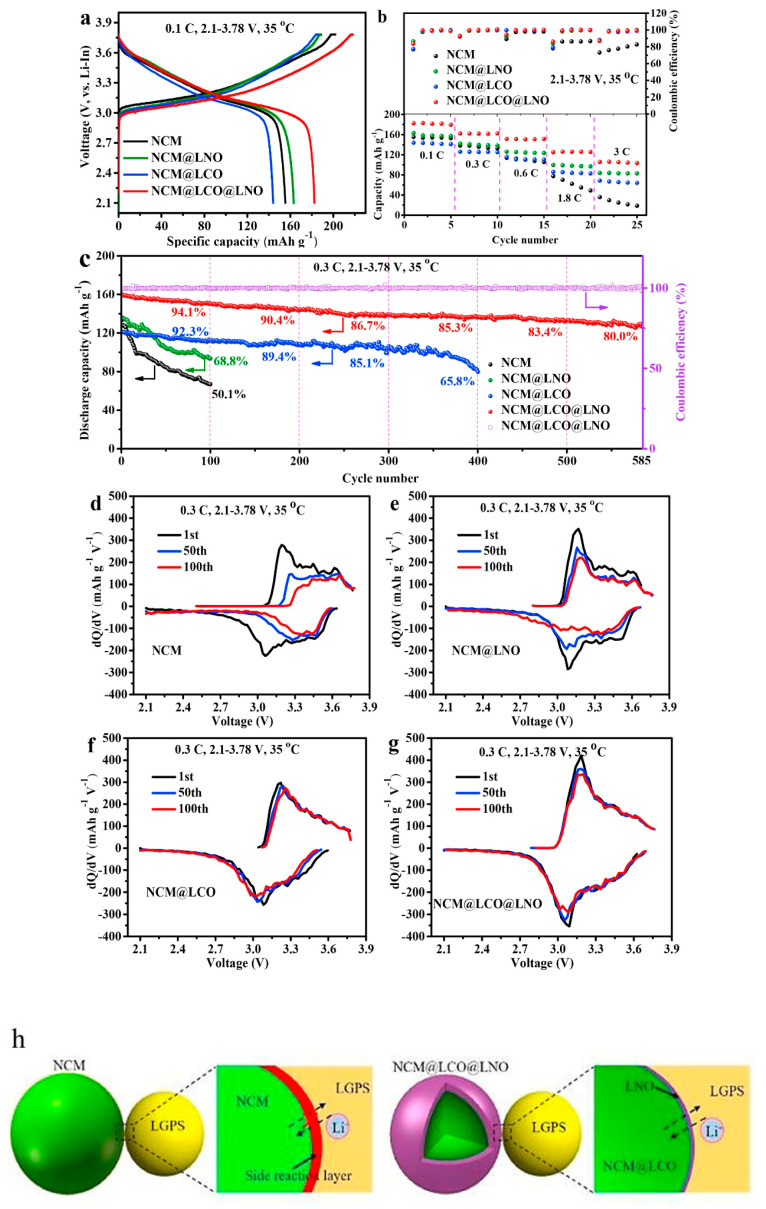
Electrochemical performances of NMC-811, NMC@LNO, NMC@LCO, and NMC@LCO@LNO cathodes for all-solid-state Li-ion batteries (ASSLB) with Li_9.54_Si_1.74_P_1.44_S_11.7_Cl_0.3_ as the solid electrolyte at 35 °C. Here NMC811, LCO, and LNO denote LiNi_0.8_Co_0.1_Mn_0.1_O_2_, Li[(Ni_0.8_Co_0.1_Mn_0.1_)_0.9_Co_0.1_]O_2_, and LiNbO_3_, respectively. (**a**) Initial charge–discharge, (**b**) rate performance, and (**c**) cycle performance curves after the rate performance test (1C = 200 mA g^−1^). (**d**)–(**g**) d*Q*/d*V* curves of the four ASSLB cathodes at the 1st, 50th, and 100th cycle at 35 °C. (**h**) Schematic diagrams of the mitigation of the side reaction by NBO coating. Reproduced with permission from [298]. Copyright 2020 Elsevier.

Recently, Li et al. [299] reported that the cells formed with a core-shell material, i.e., LiNi_0.8_Co_0__.1_Mn_0__.1_O_2_ (NMC-811) and LiNbO_3_-coated LiCoO_2_ (LNO@LCO), and Li_9__.54_Si_1__.74_P_1__.44_S_11.7_Cl_0.3_ (73:27) pressed at 280 MPa, and a 10 mm Li-In alloy foil disk pressed at 300 MPa as the cathode active materials, solid electrolyte, and anode, respectively, presented good cycling stability. They used a cathode mass loading of approximately 14.0 mg cm^−2^ and voltage range of 2.1–3.8 V for their experiments. The LNO-coated NMC@LCO cathode presented a reversible capacity of 197 mAh g^−1^ and high cycle performance with a capacity retention of 82.3% after 500 cycles at 35 °C and a current rate of 0.3C (Figure 21a–h). Recently, Zhang et al. [300] prepared the above electrolyte via elemental synthesis gasifying separation route and carbothermal reduction ethanol-dissolution technique to synthesize pure SiS_2_ and Li_2_S raw materials and they obtain a conductivity of 1.5 mS cm^−1^.

In 2012, Ooura et al. [301] prepared the (100−*x*)Li_3_PS_4_·*x*LiAlS_2_ (mol%) amorphous glassy electrolyte system via high-energy ball milling. When *x* = 0–13.1, the obtained samples were amorphous and when *x* ≥ 18.2, a crystalline Al_2_S_3_ phase formed. Among all samples, the one with *x* = 13.1 presented the best conductivity of 6.0 × 10^−4^ S cm^−1^ at 20 °C; in addition, the *E*_a_ of the sample was 39 kJ mol^−1^. The Li_4.4_Si|a-86.9Li_3_PS_4_·13.1LiAlS_2_|LiNi_1/3_Mn_1/3_Co_1/3_O_2_ ASSB was fabricated and the NMC cathode delivered an initial discharge capacity of 100 mAh g^−1^ at a current density of 0.1 mA cm^−2^ in the potential range of 2.0–4.0 V. The capacity faded during cycling owing to interfacial reactions. At the end of the 35th cycle, the specific capacity was 185 mAh g^−1^ when TiS_2_ was used as the cathode at the current rate of 64 μA cm^−2^ in the potential range of 1.0–2.5 V. Zhou et al. [300] synthesized the Li_11_AlP_2_S_12_ electrolyte, which presented a thio-LISICON analogous structure. The conductivity of this electrolyte was 8.02 × 10^−4^ S cm^−1^ at 25 °C and its *E*_a_ was 25.4 kJ mol^−1^ (0.254 eV) showing an excellent electrochemical stability up to 5 V against Li metal.

## 4. Oxide Solid Electrolytes

Oxide electrolyte materials present large energy gaps between their valence and conduction bands, which confer them high stability at high voltages; furthermore, the ionic mobility of oxide electrolytes is higher than that of glass or polymer electrolytes [29,305,306,307,308,309,310,311,312,313,314,315,316,317,318,319,320,321,322,323,324,325,326,327,328,329,330,331,332,333,334,335,336,337,338,339,340,341,342,343,344,345,346,347,348,349,350,351,352,353,354,355,356,357,358,359,360,361,362,363,364,365,366,367,368,369,370,371,372,373,374,375,376,377,378,379,380,381,382,383,384,385,386,387,388,389,390,391,392,393,394,395,396,397,398,399,400,401,402,403,404,405,406,407,408,409,410,411,412,413,414,415,416,417,418,419,420,421,422,423,424,425,426,427,428,429,430,431,432,433,434,435,436,437,438,439,440,441,442,443,444,445,446,447,448,449,450,451,452,453,454,455,456,457,458,459,460,461,462,463,464,465,466,467,468,469,470,471,472,473,474,475,476,477,478,479,480,481,482,483,484,485,486,487,488,489,490,491,492,493,494,495,496,497,498,499,500,501,502,503,504,505,506,507,508,509,510,511,512,513,514,515,516,517,518,519,520,521,522,523,524,525,526,527,528,529,530]. Table 3 summarized the structural and electrical properties of various oxide solid electrolytes. Oxide electrolytes are relatively stable in air and easier to handle than sulfide electrolytes. In 1976, Goodenough et al. [305] conducted Na^+^ ion transport studies on Na_1+*x*_Zr_2_Si*_x_*P_3-*x*_O_12_, which presented a conductivity of ≤ 5 S cm^−1^ at 300 °C for *x* ≈ 2; the observed conductivity value was comparable to that of β-alumina [306], which was one of the best solid electrolytes at the time. Furthermore, it was mentioned that the exchange of Na^+^ ions with Li^+^, Ag^+^, and K^+^ ions was possible. This early concept led to the further development, applications, and search for new Li-analogues, and the promising NASICON-type structure series of materials were explored owing to their structural framework and high Li^+^ ion conductivities at room and elevated temperatures.

In 1966, Otto [307], following from the work. in 1978 by Levasseur et al. [308,309], conducted more studies on borate-type amorphous oxide-based glassy electrolytes, and their conductivities were >10^−4^ and 10^−6^ S cm^−1^ at 350 and 25 °C, respectively. In 1973, West [310] prepared Ge-, Ti-, and Zn-doped Li_4_SiO_4_ electrolytes and reported conductivities in the range of 10^−3^–10^−4^ S cm^−1^ at 300 °C. In 1977, Shanon et al. [311] described a series of electrolyte systems, viz. Li_2+*x*_C_1−*x*_B*_x_*O_3_, Li_3−*x*_B_1−*x*_C*_x_*O_3_, Li_4+*x*_Si_1−*x*_Si_1−*x*_Al*_x_*O_4_, Li_4−*x*_Si_1−*x*_P*_x_*O_4_, Li_4−2*x*_Si_1−*x*_S*_x_*O_4_, and Li_5−*x*_Al_1−*x*_Si*_x_*O_4_. Li_0.8_Zr_1.8_Ta_0.2_P_3_O_12_. Subsequently, many researchers attempted on the electrolytes as additives or electrolytes.

Different types of oxide electrolyte systems based on NASICON-, perovskite-, and garnet-type crystalline materials have been reported in the literature [312,313,314,315,316,317,318,319,320,321,322,323,324,325,326,327,328,329,330,331,332,333,334,335,336,337,338,339,340,341,342,343,344,345,346,347,348,349,350,351,352,353,354,355,356,357,358,359,360,361,362,363,364,365,366,367,368,369,370,371,372,373,374,375,376,377,378,379,380,381,382,383,384,385,386,387,388,389,390,391,392,393,394,395,396,397,398,399,400,401,402,403,404,405,406,407,408,409,410,411,412,413,414,415,416,417,418,419,420,421,422,423,424,425,426,427,428,429,430,431,432,433,434,435,436,437,438,439,440,441,442,443,444,445,446,447,448,449,450,451,452,453,454,455,456,457,458,459,460,461,462,463,464,465,466,467,468,469,470,471,472,473,474,475,476,477,478,479,480,481,482,483,484,485,486,487,488,489,490,491,492,493,494,495,496,497]. Among all compositions, the garnet-based Ta-, Ga-, Al-doped Li_7_La_3_Zr_2_O_12_ (LLZO) and Li_1.3_Al_0.3_Ti_1.7_(PO_4_)_3_ (LATP) oxides have been well studied for ASSBs owing to their good conductivities. Note that most of the ceramic solid electrolytes (LLZO, LATP) are polycrystalline and demonstrate grain/grain-boundary microstructure (see Section 2).

### 4.1. Garnet-Type Electrolytes

Garnet-based Li^+^ ion conductors are attractive candidates for ASSBs owing to their high chemical stability when paired with Li metal, and good ionic conductivity. Several seminal articles on the synthesis of Li-stuffed garnets [312], Li_5_La_3_*M*_2_O_12_ (*M* = Nb, Ta) [313], Li_6_*A*La_3_Ta_2_O_12_ (*A* = Sr, Ba) [314], and Li_7_La_3_Zr_2_O_12_ named as Li_5_, Li_6_, and Li_7_ phases, respectively, have been published between 2003 and 2007. Among all, LLZO presented good room-temperature ionic conductivity in the range of 10^−3^–10^−4^ S cm^−1^. This led to the further search for and optimization of fast ion conducting ASSB oxide electrolytes. Hundreds of papers have been published on the synthesis, doping, and ionic conductivity of ASSB electrolytes, and only a few on their fabrication. Thangadurai et al. [26], Samson et al. [73], Ramakumar et al. [314], and Zhao et al. [315] reviewed garnet-based electrolytes, and their most important findings are summarized below.

**Figure 22:**
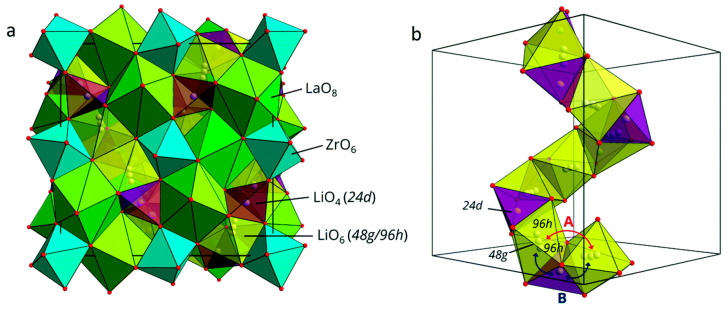
(**a**) Crystal structure of cubic Li_7_La_3_Zr_2_O_12_ (LLZO) and (**b**) Wyckoff positions of the Li^+^ ions. The centers of the tetrahedral and octahedral sites are denoted as 24*d* and 48*g* sites, respectively, and the 96*h* sites are slightly displaced off the 48*g* sites; LiO_6_ and LiO_4_ connections and two possible Li migration pathways (A and B); pathway B is the most likely Li^+^ ion mechanism migration in LLZO. Reproduced with permission from [73]. Copyright 2019 Royal Society of Chemistry.

(i) The general formula of garnet-based materials is *A*_3_*B*_2_(*X*O_4_)_3_, where *A* = Ca, Mg, La, Y, or rare earth metals; *B* = Al, Fe, Ga, Ge, Mn, Ni, or V; and *X* = Si, Ge, or Al. In addition, *A*, *B*, and *X* are eight-, six-, and four-O coordinated cation sites, respectively. The typical crystal structure of Li_7_La_3_Zr_2_O_12_, a Li-based cubic garnet, is illustrated in Figure 22a,b [73]. Li atoms randomly and partially occupy the interstices of the framework structure within two types of sites: The tetrahedral 24*d* and octahedral 48*g* or off-centered 96*h* and 96*h* sites are displaced off the 48*g* sites, the framework contains eight-fold coordinated LaO_8_ dodecahedra (24*c*) and six-fold coordinated ZrO_6_ octahedra (16*a*). The 48*g* to 96*h* site displacement is ascribed to the Li^+^–Li^+^ repulsions across shared site faces. The 24*d* tetrahedral cage faces are face-shared with four neighboring octahedral cages and form a 3D network of conduction pathways (a segment of this network is illustrated in Figure 22b).

(ii) Various synthesis strategies, including solid-state synthesis [73], ball milling [316], wet-chemical solution (sol-gel) methods [317], combustion synthesis [318], electrospinning [319], molten salt methods [320,321], spark plasma sintering (SPS) route [322,323], and the pulsed laser deposition (PLD) technique [324], could be used to stabilize the cubic structure. The reaction conditions, such as temperature and sintering time, and also *M*-site doping have been reported for the Li_3_*M*_3_Te_2_O_12_ (*M* = Y, Pr, Nd, Sm, Lu) Li_3_-phases, Li_5_La_3_*M*_2_O_12_ (*M* = Nb, Ta, Sb) Li_5_-phases, Li_6_*A*La_3_*M*_2_O_12_ (*A* = Mg, Ca, Sr, Ba; *M* = Nb, Ta) Li_6_-phases, and Li_7_La_3_*M*_2_O_12_ (*M* = Zr, Sn) Li_7_-phases. Among all phase series, the Li_7_-phases present promising potential as ASSB electrolytes owing to their high ionic conductivity and good stability when paired with Li metal.

(iii) Most Li_3_-, Li_5_-, Li_6_-, and Li_7_-garnet phases present cubic lattices, and their lattice parameters are in the ranges of 12.15–12.56, 12.66–13.06, 12.69–13.0, and 12.82–13.0 Å, respectively. Li_7_La_3_Zr_2_O_12_ presents both cubic and tetragonal phases (*a* = 13.12 Å, *c* =12.66 Å); Li_7_La_3_*M*_2_O_12_ (*M*= Zr, Sn, Hf) and Li_7_Nd_3_*M*_2_O_12_ present only tetragonal lattices (*a* = 12.94–13.12 Å and *c* =12.63–12.71 Å) [26].

(iv) The Li^+^ ion conductivity of the garnet-type electrolytes increases with increasing Li content in the garnet structure, and the maximum Li^+^ ion conductivity was achieved when the Li content was in the range of 6.4–7.0.

(v) Among all Li_7−*x*_La_3_Zr_2−*x*_Ta*_x_*O_12_ Ta-doped compounds, materials with the cubic structure (*x* = 0.25) reported by Allen et al. [317] presented a bulk Li^+^ ion conductivity of 0.87 × 10^−3^ S cm^−1^ and *E*_a_ of 0.22 eV (Table 3); in addition, the ionic conductivity and *E*_a_ of Li_6.15_La_3_Zr_1.75_Ta_0.25_Al_0.2_O_12_ were 0.37 × 10^−3^ S cm^−1^ and 0.30 eV, respectively, and those of Li_6.15_La_3_Zr_1.75_Ta_0.25_Ga_0.2_O_12_ were 0.41 × 10^−3^ S cm^−1^ and 0.41 eV, respectively [317]; moreover, the ionic conductivity of Li_7−*x*_La_3_Zr_2−*x*_Ta*_x_*O_12_ (*x* = 0.6) at 25 °C was 1.0 × 10^−3^ S cm^−1^ [325]. Owing to the good conductivity and stability of Ta-doped LLZOs, many researchers focused on the optimization of sintering temperature and synthesis techniques.

(vi) The ionic conductivity of the tetragonal polymorph of Li_7_La_3_Zr_2_O_12_ was one to two orders of magnitude lower than that of the cubic phase, particularly at low temperatures.

(vii) All Ta-doped garnets presented good chemical stability when paired with Li metal at potentials of up to 6 V vs. Li^+^/Li at room temperature [26].

(viii) The cubic phase of Li_6.25_La_3_Zr_2_Al*_x_*O_12_ (*x* = 0.2–0.3) can be stabilized via intrinsic Al-doping at high temperatures from the reaction with the Al crucible used for preparation. The ionic conductivity of the low-temperature synthesized bare LLZO (1 × 10^-6^ S cm^−1^) was approximately two orders of magnitude lower that than that of Al-doped LLZO (σ = 2 × 10^-4^ S cm^−1^) [496].

(ix) The Li^+^ ion conduction mechanism was analyzed using solid-state NMR experiments [326] and computational calculations, indicating that the Li conduction occurred mostly between the octahedral sites. Moreover, the Li^+^ ions that occupied those sites were connected to each other in a 3D network that allowed the Li^+^ ions to hop from one edge of the shared octahedra to another. Furthermore, the Li^+^ ion conduction pathways appear to be correlated with the concentration of Li in the garnet structures [26].

(x) Li–garnet-based oxide electrolytes undergo proton exchange reactions in water, aqueous LiCl/LiOH solutions, and dilute acids, and the exchange appears to be favored at the tetrahedral sites. Li_5_La_3_*M*_2_O_12_ undergoes proton exchange reactions more readily than other Li-rich phases, such as the Li_6_- and Li_7_-garnet phases. More details on the chemical and electrochemical stability in aqueous solution or in the presence of moisture/humidity, CO_2_, and Li metal are included in the recent review published by Hofstetter et al. [327].

(xi) Few researchers have focused on the chemical stability of LLZO solid electrolytes paired with LiFePO_4_, LiCoO_2_, LiMn_2_O_4_, LiCoMnO_4_, LiFe_0.5_Mn_1.5_O_4_, LiNi_0.5_Mn_1.5_O_4_, Li(Ni_1/3_Co_1/3_Mn_1/3_)O_2_ (NMC) cathode materials [26,328,329]. For these studies, typically 1:1 w/w mixtures of electrolytes and cathodes were used, and the electrolytes were sintered in the temperature range of 800–900 °C. Among all cathodes, LCO and NMC111 presented better stability when paired with Ta-LLZO electrolytes. Few reports indicated that the additional reactive phases that formed during sintering were LaCoO_3_, Co_3_O_4_, or La_2_Zr_2_O_7_.

(xii) SSBs were fabricated using different forms of electrolytes, i.e., solid, bare, and composite semi-solid/liquid electrolytes, and few efforts were devoted to sintering them with additives like Li_3_BO_3_, Li_2.3_C_0.7_B_0.3_O_3_, Li_3_PO_4_, and Li_4_SiO_4_. The melting points of Li_3_BO_3_ and Li_2.3_C_0.7_B_0.3_O_3_ of 700 and 690 °C, respectively, were the lowest of all analyzed solid electrolytes [330,331,332]. Ohta et al. [333] fabricated an ASSB using Nb-doped LLZO as the solid electrolyte and Li_3_BO_3_ as the solid electrolyte mixed with the LiCoO_2_ cathode. Few case studies on SSBs are discussed in detail in the following. The reactivity of the cathode–electrolyte pairs varies with the reaction temperature, reaction time, and sintering conditions, such as the pressure and atmosphere (air, Ar, or O_2_).

(xiii) Critical current limits have been studied, and it was revealed that Li plating occurred at current densities above ~0.5–1.0 mA cm^−2^ during the charging penetration of Li in the solid electrolyte [334,335], which led to short circuiting. This low operating current limits the use of these oxide electrolytes for large-scale electric vehicle battery applications, which require discharge current rates in the range of ~1–10 mA cm^−2^.

(xiv) Gong et al. [336] performed in situ TEM studies on Ag|Ta-LLZO|LCO and revealed that the Li extraction mechanism in solid electrolytes was different than in liquid electrolytes; moreover, hexagonal phase transitions occur when LCO was cycled using commercial liquid electrolytes [337]. Based on TEM observations, LCO single crystal became a polycrystalline material with 5–15 nm grains after delithiation and formed coherent twin boundaries and antiphase domain boundaries along its (010) axis.

(xv) Researchers have determined that the shortcomings at the LLZO/electrode interfaces, for both the Li anode and cathode, must be addressed using advanced techniques to render solid-state Li-ion batteries useful for commercial large-scale applications. The interface drawbacks of SSBs have been highlighted in 1986 by Hagenmuller [338] at the international seminar on solid-state devices in Singapore. He mentioned the need for stable highly conductive electrolytes, the concerns associated with the fabrication technology, and highlighted the importance of the cooperation between scientists and engineers [339].

Thangadurai et al. [26] and Samson et al. [73] reviewed the literature on LLZO electrolytes published until early 2019. Herein, we discuss a few additional, more recent publications on LLZO electrolytes, as follows. Posch et al. [340] studied the ion dynamics of Al-doped Li_6.46_Al_0.15_La_3_Zr_1.95_O_11.86_ (Al-LLZO) using solid-state NMR and conductivity measurements. The measured ionic conductivity of Al-LLZO (8.3 × 10^−5^ S cm^−1^) was slightly lower than the value 10^−4^ S cm^−1^ reported for polycrystalline Al-LLZO [26]. It was noted that when the Al content was optimal (0.2–0.3 mol.% Al^3+^) the Al-LLZO samples reached conductivities of up to 10^−3^ S cm^−1^. Solid-state NMR spin-lattice relaxation measurements revealed that the *E*_a_ of the samples was in the range of 0.18–0.38 eV; these values describe both the local barriers of the elementary jump processes and diffusion on a wider length scale, and were similar to that obtained via conductivity measurements (*E*_a_ = 0.36 eV). Marbella et al. [341] performed solid-state NMR analysis on the Li|Li_6.5_La_3_Zr_1.5_Ta_0.5_O_12_|Li solid electrolyte system during Li-stripping and plating and noted that the growth of Li dendrites increased with increasing cycle time; moreover, dense Li microstructures that grew into the electrolyte pellet surface were observed before short-circuits occurred during the electrochemical measurements at low current rates < 0.5 mA cm^−2^.

Recently, Bock et al. [342] reported that the thermal conductivity of Li_7_La_3_Zr_2_O_12_ was approximately 0.47 ± 0.009 W K^−1^ m^−1^. Moreover, de Klerk and Wagemaker [343] reported the mathematical space charge model of the LLZO electrolyte and electrode materials, such as graphite and LCO. In addition, Binninger et al. [276] determined the electrochemical stability window of the LLZO electrolyte using computational techniques. Few other reports on doping Li_7_-garnet series have been recently published [344,345,346,347]. Farooq et al. [344] reported that the ionic conductivities of the Ba-doped Li_6.5_La_2.5_Ba_0.5_TaZrO_12_ solid electrolytes sintered at 1100 to 1200 °C were 1.07 × 10^−6^ and 6.62 × 10^−5^ S cm^−1^, respectively, at 26 °C. In addition, Huo et al. [322] used other dopants to substitute the La sites of the Li_6.5_La_2.5_*A*_0.5_TaZrO_12_ (*A* = Ca, Sr, Ba) compounds via SPS, and among all, the Sr-doped garnets presented the highest Li^+^ ion conductivity of 3.08 × 10^−4^ S cm^−1^ at 20 °C and lowest *E*_a_ of 0.35 eV. Furthermore, they analyzed the effect of structural stability, ion mobility, and interfacial mechanisms during air exposure.

Kotobuki and Koishi [323] prepared the dense (99.7%) Y-doped LLZO (Li_7.06_La_3_Zr_1.94_Y_0.06_O_12_, LLYZ) solid electrolyte using the SPS technique. The samples were sintered in the temperature range of 800–1100 °C for 10 min and under the pressure of 40 MPa, and the reported total conductivity of the pellet sintered at 1100 °C was 9.8 × 10^−4^ S cm^−1^, which was higher than that of the pellet prepared using the conventional synthesis method; moreover, the sample presented good stability in the potential window of 0–9.0 V vs. Li^+^/Li. Recently, Paolella et al. [345] studied the effect of chemical phase evolution of bare and doped LLZO in relation with the Li loss at high temperature.

Owing to their good electrolyte/cathode interface properties, a series of polymer solid composite electrolyte have been developed for Li batteries. After the introduction of the polymer electrolyte concept for Li batteries by Armand [346], many attempts have been made to use polymers and metal oxides, such as TiO_2_ and SiO_2_, as solid electrolytes. Mei et al. [279] measured the ionic conductivity of PEO_18_–LiClO_4_–*x* wt.% Li_6.4_La_3_Zr_1.4_Ta_0.6_O_12_. Zhang et al. [347] prepared organic–inorganic composite protective membranes that consisted of poly(vinylidene fluoride-co-hexafluoropropylene) (PVDF-HEP) and LLZO composites using the tape-casting method. Xu et al. [348] synthesized a LLZO/polyacrylonitrile composite with gel polymer electrolyte used in cell with LiFePO_4_ cathode. Gao et al. [349] studied the performance of the lithiated Nafion (Li-Nafion)-garnet ceramic Li_6.25_La_3_Zr_2_Al_0.25_O_12_ (LLZAO) composite in LiFePO_4_||Li cell at 30 °C and reported that the specific discharge capacity of the cell was 160 mAh g^−1^, its capacity retention was 97% after 100 cycles at a current rate of 0.2C, and the retained capacity after 500 cycles at 1C was 126 mAh g^−1^. Liu et al. [350] studied the Ta-LLZO/liquid electrolyte interface. Zhang et al. [351] used a SPE-based composite with lithium bis(trifluoromethanesulfonyl)imide (LiTFSI) as Li salt and reported that the cell with 10 wt.% PEO-LiTFSI Li_6.7_La_3_Zr_1.7_Ta_0.3_O_12_ composite solid electrolyte and LiFePO_4_ cathode delivered a reversible capacity of 140 mAh g^−1^ at the current rate of 0.2C at 60 °C; moreover, the cell retained a capacity of 139 mAh g^−1^ after 200 cycles.

Thangadurai et al. [26] and Samson et al. [73] dedicated considerable efforts to the analysis of the fundamental aspects of garnet electrolytes. In addition, Lobe et al. [352,353], who are considered experts in the fabrication of solid oxide fuel cells, explored the fabrication of ASSBs using thin film deposition. Furthermore, Tsai et al. [13,335] evaluated the screen-printing technique and investigated the sol–gel and solid-state preparation methods.

**Figure 23:**
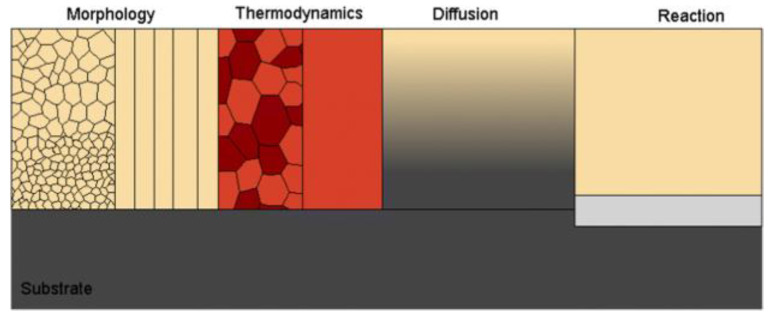
Schematic representation of factors that are affected by the substrate temperature during the thin-film deposition of garnet-structured electrolytes. Reproduced with permission from [352]. Copyright 2018 Springer.

Herein, we summarize a few recent advances on the fabrication technology of garnet electrolytes, which could lead to further improvements in the fabrication technology of ASSBs. Lobe et al. [352,353] reported the challenges of thin film deposition of garnet electrolytes for ASSBs. In addition, they analyzed the ionic conductivity of garnet-structured thin films obtained using the radio-frequency (RF)-sputtering deposition technique, and optimized the deposition parameters such as the substrate temperature, power, total pressure, and target substrate distance required to achieve films with optimal chemical composition, morphology, thermodynamics, diffusion, and reactivity (Figure 23). They noted that the large-scale fabrication of batteries would be hindered owing to the high sintering temperature. In addition, appropriate, inexpensive, low reactive substrates and well-sintered and high-ionic-conductivity membranes with optimum composition, which must be nonreactive with the cathode or electrolyte, would be needed.

**Figure 24:**
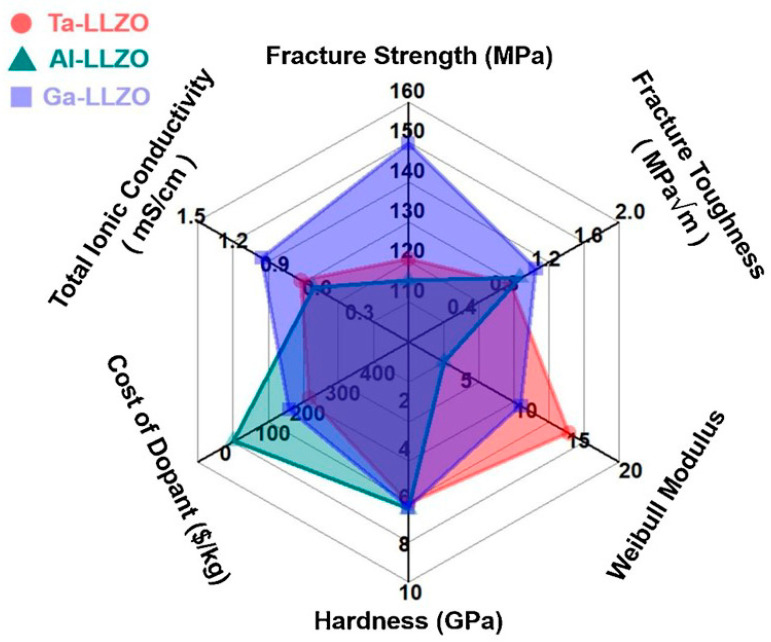
Spider chart of mechanical and electrical properties of hot-pressed Ta-LLZO, Al-LLZO, and Ga-LLZO. Here, LLZO denotes Li_6.25_La_3_Al_0.25_Zr_2_O_12_. Reproduced with permission from [355]. Copyright 2020 Elsevier.

Wang at al. [354] analyzed the effects of the stack pressure on the conductivity of LLZO electrolytes. Recently, Han et al. [355] studied the mechanical and electrical properties of hot-pressed Ta-, Al-, and Ga-doped LLZO fabricated at a constant pressure of 47 MPa for 40 min in Ar flow as follows: Li_6.25_La_3_Al_0.25_Zr_2_O_12_ (Al-LLZO) at 1225 °C, Li_6.50_La_3_Ta_0.50_Zr_1.5_O_12_ (Ta-LLZO) at 1225 °C, Li_6.25_La_3_Ga_0.25_Zr_2_O_12_ (Ga-LLZO) at 1100 °C. They noted that the Ga-doped LLZO possessed the highest fracture stress (~143 MPa) and fracture toughness followed by Ta-LLZO and Al-LLZO. The mechanical properties and costs of all dopants are summarized in Figure 24. The room-temperature bulk and (total) conductivities of 5.9 mm thick Au-coated Al-LLZO, Ta-LLZO, and Ga-LLZO pellets were determined to be 0.75 (0.68), 0.79 (0.75), and 1.5 (1.04) mS cm^−1^, respectively (see Table 3). The bulk and total conductivities of thinner (1.2–1.3 mm) pellets were similar. Therefore, Ga was considered to be the best dopant in this study, owing to its cost and mechanical properties of the doped samples. Other properties, such as the chemical and structural stability achieved when these cathodes were paired with Li metal anodes or the cathode/electrolyte interface properties, were not evaluated in this paper; however, these parameters are very important for the fabrication of ASSBs.

Recently, Tsai et al. [13] studied the ASSB formed when Ta-doped Li_6.6_La_3_Zr_1.6_Ta_0.4_O_12_ (LLZO) solid electrolyte fabricated via solid-state sintering at 1175 °C in air was paired with LCO as the cathode without interface modifications. Ta-doped LLZO was used as the electrolyte owing to its good chemical stability when paired with the LCO cathode, which is known to be the highest electronic conductivity. The thermal expansion coefficient of LLZO (1.5 × 10^−5^ K^−1^) was similar to that of LCO (1.3 × 10^−5^ K^−1^). To fabricate the ASSB, LCO and Ta-LLZO (1:1 w/w) were weighed and milled using Y-stabilized zirconia balls and ethanol as the solvent for 24 h to reduce the particle size distribution range to *D*(n, 0.5) = 1.03 µm followed by drying the slurry at 60 °C. Then, the screen-printing ink slurry was prepared by a three-roll milling using composite powder (5 wt.%), 6 wt.% ethyl cellulose in terpineol (3 wt.%):8250 thinner (2 wt.%). A brush was used to paint the ink on ~0.6 mm thick Ta-LLZO discs, which were cut using a diamond saw, at 55 °C in air. Subsequently, the painted disks were heated to 600 °C (heating rate of 2 °C min^−1^) followed by heating to 1050 °C in air for 30 min in a tube furnace. After sintering, the non-painted side of the Ta-LLZO disk was polished to remove impurities using SiC paper (~300 µm) and the surface was cleaned via plasma etching. Lastly, a thin Au film was sputtered on the surface of the composite electrode, electrolyte, and top surface of the Ta-LLZO disk using a desktop sputter coater to facilitate In adhesion. An indium foil was used as the anode to improve the interface with Ta-LLZO heated up 200 °C on a hot plate, before placing it into a Swagelok cell.

**Figure 25:**
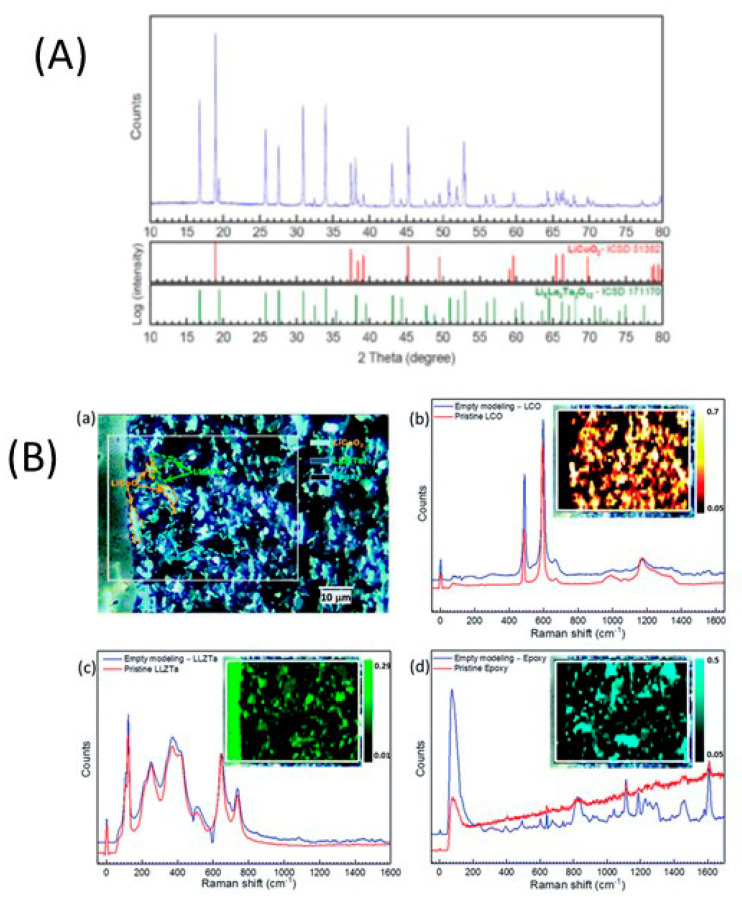
(**A**) X-ray diffraction patterns of LiCoO_2_/Li_6.6_La_3_Zr_1.6_Ta_0.4_O_12_ composite cathode with the mass ratio of 1:1 that was sintered at 1050 °C for 30 min in air. (**B**) High-resolution micro-Raman mapping of the cross-section of the ASSLB. (**a**) Optical image of the ASSLB cross-section and its mapping area. Raman mappings and spectra of (**b**) LiCoO_2_, (**c**) Ta-LLZO, and (**d**) epoxy. Reproduced with permission from [13]. Copyright 2019 Royal Society of Chemistry.

**Figure 26:**
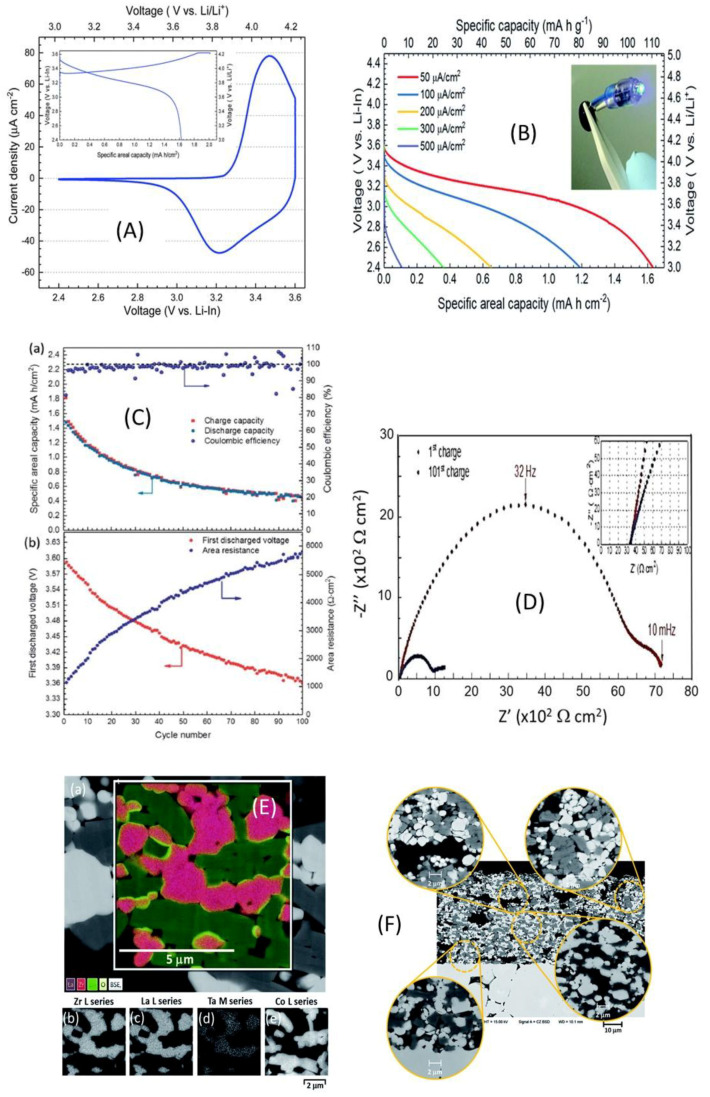
(**A**) Cyclic voltammogram of the LCO|Ta-LLZO|Li-In ASSB collected in the voltage range of 2.4–3.6 V *vs.* Li–In. The inset illustrates the first cycle charge–discharge performance of the SSLB at a constant current density of 20 μA cm^−2^ before it was subjected to CV scanning. (**B**) Discharge profile of the SSLB at different current densities. The discharge profiles of the cell were obtained in sequence from the lowest to the highest current density. Therefore, the capacity fading owing to the cycling of the cell was not taken into account for capacity calculations. The inset depicts the SSLB, which features a black composite polymer electrolyte in front, which lights up an LED. (**C**) Long-term charge–discharge cycling of SSLB (**a**), and first discharge voltage points for the cycles and calculated area resistance of the SSLB (**b**). (**D**) Electrochemical impedance spectroscopy diagram of the SSLB before and after long-term galvanostatic cycling. (**E**) (a) SEM and energy-dispersive X-ray spectroscopy (EDS) mapping of the sintered composite positive electrode. Monochromatic EDS mappings of (**b**) Zr, (**c**) La, (**d**) Ta, and (**e**) Co. (**F**) Scanning electron microscopy (SEM) cross-section images of the SSLB that underwent 100 galvanostatic charge–discharge cycles at 50 °C. Reproduced with permission from [357]. Copyright 2019 Royal Society of Chemistry.

No reaction byproducts of LCO or Ta-LLZO were observed in the XRD profile and Raman spectra of the composites sintered at for 1 h at 1050 °C in air (Figure 25A,B) [13]. The LaCoO_3_ or Co_3_O_4_ phases were absent from the high-resolution Raman spectra and a weak band at 689 cm^−1^ was observed in the spectrum of the Ta-LLZO grains, which indicated that the concentration of Co that was diffused into the Ta-LLZO grains was low. The calculated ionic transport number of the sintered Ta-LLZO was ~1, which indicated the negligible self-discharge of the fabricated ASSB. A good reversible peak at 3.47/3.20 V vs. Li-In (4.09/3.82 V vs. Li^+^/Li) was observed in the cyclic voltammogram of the battery during the anodic (positive) and cathodic (negative) scans (Figure 26A–F). This was the first time well-defined LCO redox peaks reported when Ta-LLZO was used as the ASSB solid electrolyte. In contrast with the use of standard liquid electrolyte, i.e., 1 mol L^−1^ LiPF_6_ (EC:DMC) with LiCoO_2_, the main redox couple peaks (~4.0/3.8 V) and other additional hexagonal phase transitions (~4.2/4.15, ~4.57/4.44, ~4.65/4.53 V) were observed as a function of the preparation temperature and Li content of molten salt synthesized LiCoO_2_ [356]. Authors noted that Li_1+*x*_CoO_2_ cathode with well sintered sample showed improved capacity due to suppression of hexagonal phase transformation.

Researchers should consider analyzing the performance of the SSB with the excess Li-doped LCO cathode. The galvanostatic charge–discharge profiles (Figure 26B) of the ASSB revealed that the first charge and discharge capacities were 2.01 mAh cm^−2^ (140 mAh g^−1^) and 1.62 mAh cm^−2^ (113 mAh g^−1^), respectively, and the irreversible capacity loss and at end of the 100th cycle was approximately 27 mAh g^−1^, because the capacity of 1.62 mAh cm^−2^ (36 mAh g^−1^) was retained [13] (see Table 4). The irreversible capacity was correlated with the decrease in the number of Li^+^ ion conduction pathways and irreversible formation of new interfaces. Irrespective of the good redox potential observed in the cyclic voltammogram (CV) of the ASSB, the capacity faded with the cycle number owing to the gradual increase in cell polarization with cycling (Figure 26).

Possible mechanisms of interface evolution were proposed using the energy-dispersive electron spectroscopy mapping of the sintered composite electrode, which revealed the presence of clean edges for La and Co between LCO and Ta-LLZO, and therefore, confirmed that no diffusion occurred during cycling. In addition, microcracks were observed on the composite electrode and electrolyte (Figure 26), which were caused by the repetitive expansion and contraction of the electrode and caused the capacity degradation of the ASSB. The pressure applied during electrochemical cycling and its effects on further technology optimization should be studied in more detail. Although LLZO-based solid state batteries are easier to handle than those using sulfide electrolytes, their capacity and cycling stability should be improved for expanding their practical applications. Overall, Ta-LLZO and LCO were sintered at 1050 °C, and it was noted that shortening the sintering time at high temperature could prevent the element inter-diffusion and minimize crack formation. In addition to bare cathode and electrolyte composite sintering, the use of coatings and additives has also been experimentally investigated. Ohta et al. [333] used Li_3_BO_3_ as an additive for Nb-doped LLZO/LiCoO_2_–Li_3_BO_3_.

**Figure 27:**
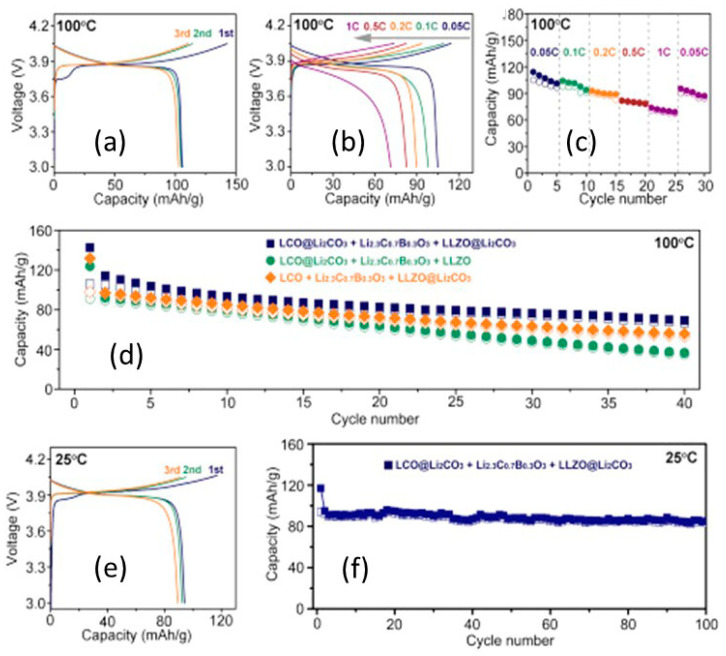
(**a**) Charge–discharge profiles of the interphase-engineered all-ceramic Li| Li_6.4_La_3_Zr_1.4_Ta_0.6_O_12_|LiCoO_2_ (Li|LLZO|LCO) cell for the first three cycles at 0.05C and 100 °C. (**b**) Charge–discharge profiles of the interphase-engineered all-ceramic Li|LLZO|LCO cell at different current rates in the range of 0.05–1C at 100 °C. The profiles at the different rates were obtained using fresh cells after one activation cycle at 0.05C. (**c**) Rate performance of the interphase-engineered all-ceramic Li|LLZO|LCO cell at 100 °C. The capacities at the different current rates were obtained using fresh cells, and each cell is represented using a different color. (**d**) Cycling performance of the interphase-engineered all-ceramic Li|LLZO|LCO cell at 0.05 C and 100 °C. The cycling performances of all-ceramic Li|LLZO|LCO cells with cathode composites consisting of uncoated LCO (LCO + Li_2.3_C_0.7_B_0.3_O_3_ + LLZO@Li_2_CO_3_) and uncoated LLZO (LCO@Li_2_CO_3_ + Li_2.3_C_0.7_B_0.3_O_3_ + LLZO) are also included. (**e**) Charge–discharge profiles of the interphase-engineered all-ceramic Li|LLZO|LCO cell for the first three cycles at 0.05 C and 25 °C. (**f**) Cycling performance of the interphase-engineered all-ceramic Li|LLZO|LCO cell at 0.05C and 25 °C. The specific capacity was calculated based on the weight of LCO in the cathode composite. Reproduced with permission from [357]. Copyright 2018 Elsevier.

Ohta et al. [333] used Li_3_BO_3_ as an additive for Nb-doped LLZO/LiCoO_2_–Li_3_BO_3_. Later, Han et al. [357] reported the low cathode/electrolyte interfacial resistance obtained by thermal soldering of the Li_2_CO_3_-coated LCO cathode and Ta-LLZO (Li_6.4_La_3_Zr_1.4_Ta_0.6_O_12_) solid electrolyte together using Li_2.3_C_0.7_B_0.3_O_3_ as additive, which has an ionic conductivity of 10^−5^ S cm^−1^ at 100 °C. The advantage of this additive is a reasonably low melting point of approximately 690 °C and can be well soldered with the Li_2_CO_3_-coated cathode and the LLZO electrolyte. Li_2.3_C_0.7_B_0.3_O_3_ powder was prepared by heating a mixture of Li_2_CO_3_ and Li_3_BO_3_ in air at 650 °C for 10 h. A thin Li_2_CO_3_ layer was deposited on LCO as follows. The as-prepared LCO was soaked in a mixed 1 mol L^−1^ LiOH and 0.25 mol L^−1^ LiNO_3_ aqueous solution for 30 min. The obtained solid was then filtered, dried in a vacuum oven, and heated to 250 °C in CO_2_ atmosphere for 5 h. Subsequently, Li_2_CO_3_ was coated on the Ta-LLZO SSE by exposing the milled powder for 1 h and then stored in air. The results of the electrochemical studies performed using 1–3 mg of active material revealed the irreversible capacity loss of 32 mAh g^−1^ during the first cycle and reasonably good stability during cycling (Figure 27a–f) [357]. The low mass of active material used in this study cannot be compared with the higher loadings reported in the literature; moreover, in this study, the high Ta doping (0.6 wt.% Ta) led to the increase in the cost of the raw materials. For practical application, the concentration of Ta should be ≤ 0.25 mole.

**Figure 28:**
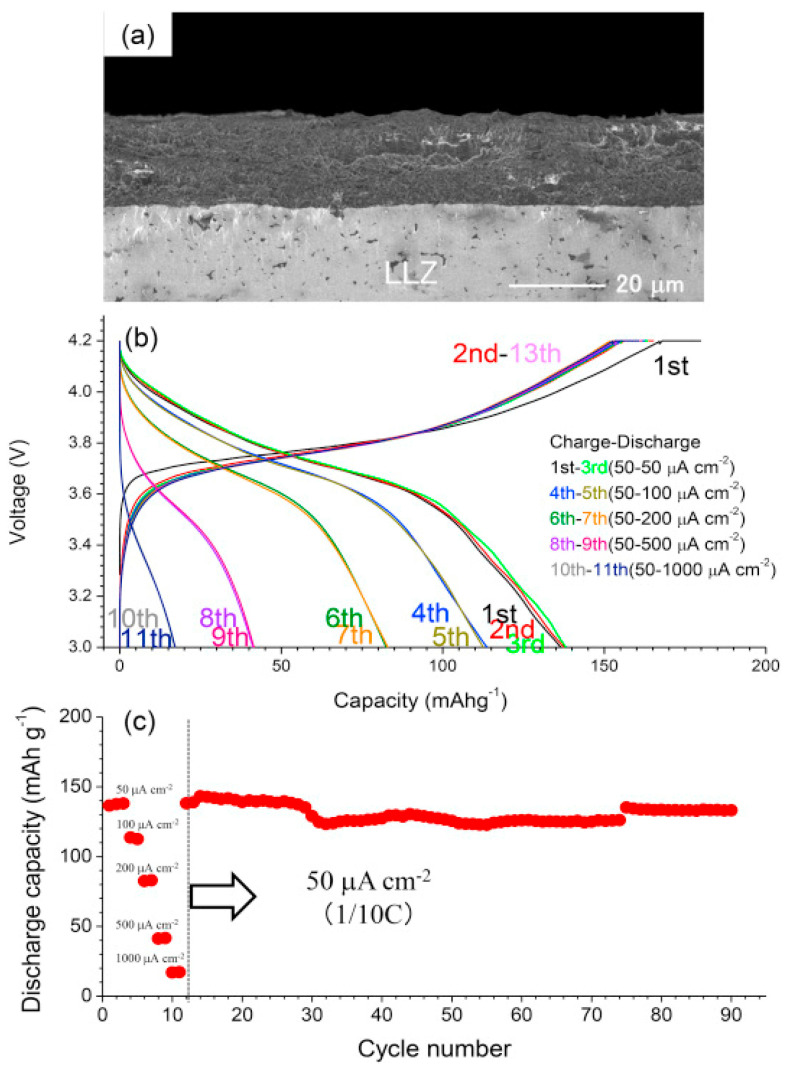
(**a**) Cross-sectional scanning electron micrograph of the LLZO/NMC-LATP composite film prepared using LTP-5. (**b**) Charge–discharge curves of the all-solid-state battery (ASSB) featuring the Li/LLZO/NMC-LATP composite film prepared using LTP-5 as the cathode. The measurements were performed at 100 °C, the charge current density was maintained at 50 μA cm^−2^, and the discharge current density was varied in the range of 50–1000 μA cm^−2^. (**c**) Specific discharge capacity of ASSB vs. cycle number. Reproduced with permission from [358]. Copyright 2016 Elsevier.

Kato et al. [358] deposited the LNMC + 5 wt.% LATP composite on LLZO pellets and reported that the areal capacity of the ASSB was 0.5 mAh cm^−2^ (specific capacity of approximately 150 mAh g^−1^) over 90 cycles at a current rate of 50 μA cm^−2^ (Figure 28a–c). In addition, the authors used stack pressure during cycling and the addition of 5 wt.% LATP to LNMC improve the interfacial contact between the electrode and electrolyte. These results should be of further interest for oxide-based electrolyte systems. Improvement of the interfacial contact between electrodes and polymer-based electrolyte composites has been obtained by mixing 10–20 wt.% LLZO with polymer, ionic liquids, and inorganic salts, such as 1 mol L^−1^ LiClO_4_ and 1 mol L^−1^ LiPF_6_. Thus, the optimization of the stack pressure during electrochemical cycling of hot-press–manufactured Ta-LLZO cathode materials is required for large-scale applications. Barai et al. [497] revealed the growth of Li dendrites through local inhomogeneities of polycrystalline LLZO-based ceramics and subsequent short-circuit of the ASSB. They developed atomistic simulations using a mesoscale model to estimate the dendrite growth velocity. Results showed that the average growth velocity increased with the lithium yield strength.

### 4.2. Li-Analogues of NASICON

**Figure 29:**
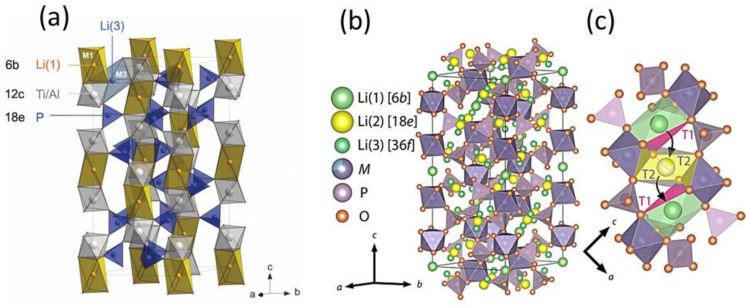
Structure of (**a**) Li_1+_*_x_*Al*_x_*Ti_2−_*_x_*(PO_4_)_3_ (LATP) and (**b**) Li_1.5_Al_0.5_Ge_1.5_P_3_O_12_ (LAGP). (**c**) The M_I_ and M_II_ intercalation sites correspond to the main occupied and excess (*x*) Li^+^ sites, respectively. Reproduced with permission from [49]. Copyright 2019 Wiley.

Sodium zirconium phosphate (NaZr_2_(PO_4_)_3_ (NZP) is the parent compound of the Na-based super ionic conductor named NASICON [359,360,361,362,363,364,365,366,367,368,369,370,371,372,373,374,375,376,377,378,379,380,381,382,383,384,385,386,387,388,389,390,391,392,393,394,395,396,397,398,399,400,401,402,403,404,405,406,407]. The crystal structure of NASICON (Na*M*_2_(PO_4_)_3_ *M* = Ge, Ti, Zr) was reported in 1968 by Hagman and Kierkegaard [359] to be hexagonal with the *R*-3/*c* space group. The crystal structure of NASICON consists of *M*O_6_ octahedra interconnected via corner sharing with PO_4_ tetrahedra, which share all their vertices to form a 3D network with interconnected channels. The Na^+^ or Li^+^ ions are located in these channels and can occupy two different sites in the crystal structure: The type I or M1 sites are six-fold coordinated directly between two *M*O_6_ octahedra; conversely, the Type II or M2 sites are eight-fold coordinated and are located between two columns of *M*O_6_ octahedra. For NZP, only the Type I sites are filled (Figure 29a–c). Cationic carriers move from one site to another through bottlenecks, and the size of the bottlenecks depends on the nature of the skeleton ions and carrier concentrations. Many efforts have been invested to chemically substitute the Na and Zr sites of NASICON and obtain a variety of isostructural Li compounds, such as Li(*M*_2_^4+^)(PO_4_)_3_, (*M* = Ti, Zr, Hf, Ge, Sn) [360,361,362,363], Li*M*^V^*M*^III^(PO_4_)_3_ (*M*^V^ = Nb, Ta; *M*^III^ = Al, Cr, Fe) [364], Li_1−*x*_*M*_2-−*x*_*M*′*_x_*P_3_O_12_ (*M* = Hf, Zr; *M*′ = Ti, Nb) [353], and Li_1+*x*_(*M*_2−*x*_^4+^,*N_x_*^3+^ )(PO_4_)_3_ (*M* = Ti, Zr, Hf, Ge, Sn; *N* = Al, Ga, In) [362]. Among the aforementioned electrolytes, hexagonal-type structures LATP and Li_1.5_Al_0.5_Ge_1.5_P_3_O_12_ (LAGP) (Figure 29) have been well studied owing to their high ionic conductivities. Although LAGP presents high ionic conductivity of up to 5 mS cm^−1^ its large-scale applications for Li batteries [49] or Li–air batteries [365] have been ruled out owing to the very high cost of Ge. 

DeWees and Wang [49] and Xiao et al. [82] have recently surveyed the literature on LATP electrolytes, and their findings can be summarized as follows.

**Figure 30:**
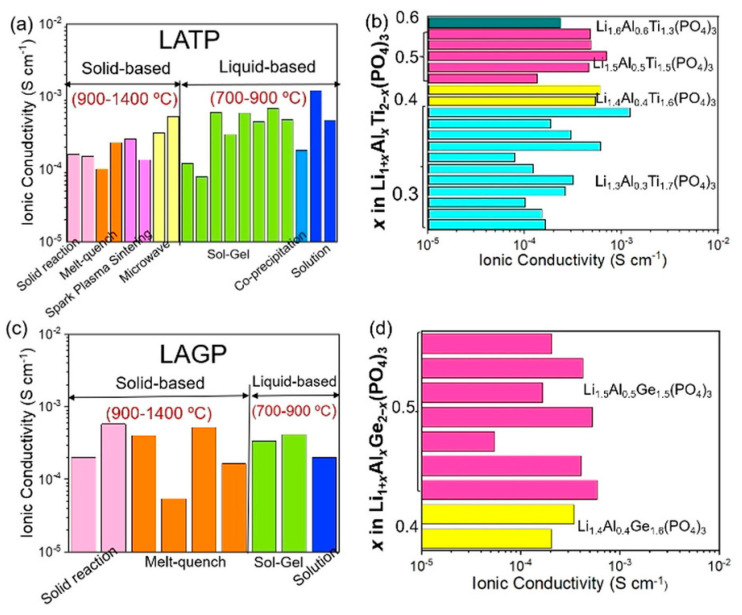
Dependence of ionic conductivities of (**a**) and (**b**) Li_1.5_Al_0.5_Ti_1.5_P_3_O_12_ (LATP) and (**c**) and (**d**) Li_1.5_Al_0.5_Ge_1.5_P_3_O_12_ (LAGP) solid electrolytes that were obtained using different synthesis methods and presented different Al contents. Reproduced with permission from [49]. Copyright 2019 Wiley.

(i) In 1986, Subramanian et al. [360] synthesized a NASICON-type LiTi_2_(PO_4_)_3_ (LTP) electrolyte and performed conductivity studies on it. The conductivity of LTP was 7.9 × 10^−8^ and 5.0 × 10^−3^ S cm^−1^ at room temperature and 300 °C, respectively. Its low conductivity and poor sinterability were disadvantageous. To improve the conductivity and densification of pellets, in 1989, Aono et al. [366] replaced a fraction of the Ti^4+^ ions (ion radius of 0.60 Å) in the parent LTP material with smaller trivalent cations, such as Al^3+^ (ionic radius of 0.53 Å) and obtained compounds such as Li_1.3_Al_0.3_Ti_1.7_(PO_4_)_3_ (LATP), and reported the successful increase in the total ionic conductivity up to 5 × 10^−4^ S cm^−1^, and the grain conductivity (without the limitations of grain boundaries, secondary phases, and porosity) of approximately 3 × 10^−3^ S cm^−1^. Later, Birke et al. [367] fabricated a Li_4_Ti_5_O_12_|Li_1.3_Ti_1.7_(PO_4_)_3_|LiMn_2_O_4_ solid-state cell with 15 wt.% (0.44 LiBO_2_:0.56LiF) additive in the cathode. Subsequently, Cretin et al. [368] prepared LATP using different synthesis routes such as sol–gel, solid-state, and co-grinding methods.

(ii) Many researchers have attempted to improve the Li^+^ ion conductivity of LATP electrolytes using different synthesis methods, such as the solid-state, sol–gel [363], melt quenching, co-precipitation [369], microwave-assisted reactive sintering, SPS [370], spray drying, spin coating [371], tape casting [372], and RF magnetron sputtering [373] methods, and different reaction conditions, such as different synthesis temperatures in the range of 700–1100 °C. Among all preparation methods, the sol–gel and solution-based ones generated LATP electrolytes with improved conductivity (Figure 30a–d). The crystallization of LATP starts at approximately 700 °C and its phase formation occurs in the range of 750–850 °C; in addition, decomposition (or phase segregation) occurs at 850 °C and leads to the formation of AlPO_4_, TiO_2_, and Li_4_P_2_O_7_ phases [374]. Further details on the synthesis of LATP can be found in recent reviews [49,82].

(iii) LATP presents a hexagonal lattice and its lattice parameters are in the ranges of *a* ≈ 8.50 Å and *c* ≈ 20.52 Å; cell volume of 1305 Å^3^. The crystal structure of LATP consists of TiO_6_ octahedra and PO_4_ tetrahedra sharing corners that are connected to form a 3D network structure (Figure 28), in which Li ions are located into two sites labeled *M*_I_ and *M*_II_. Three different Li sites (Li(1), Li(2), and Li(3)) can be distinguished in the LATP (or LiGe_2_(PO_4_)_3_) structure [49]. The Li(1) sites are expected to be fully occupied, whereas the Li(2) and Li(3) sites are only partially occupied. The increase in conductivity of LATP was correlated with the increase in the *M*–O bond strength and decrease in the Li–O bond strength upon the partial substitution of Ti^4+^ ions with Al^3+^ ions.

(iv) Nairn et al. [375] and Vinod-Chandran et al. [376] studied the Li^+^ ion conductivity and evaluated the diffusion coefficients of LATP via NMR. The obtained lithium diffusion coefficients and activation energies are in the range 0.3-5.0 × 10^-8^ cm^2^ s^−1^ and 0.16–0.17 eV, respectively, and the conductivity is close to 10^−3^ S cm^−1^ at 27 °C (Table 3) [377].

(v) Additives have been reported to improve the ionic conductivity of LATP. For example, the product obtained by sintering of a mixture of Li_2.9_B_0.9_S_0.1_O_3.1_ and LATP (mole ratio of 1:9) at 800 °C presented a total conductivity of 1.5 × 10^−5^ S cm^−1^ at room-temperature [378].

(vi) Owing to its high Li^+^ ion conductivity, LATP is an important ASSB ceramic electrolyte; however, when Li metal is used as the anode, the LATP membrane has to be separated from it using an additional protective layer to avoid the Ti^4+^/Ti^3+^ reduction reaction, because the presence of this redox couple during electrochemical cycling leads to slow structural phase transitions and lowers the Li^+^ ion conducting properties of the LATP electrolyte during cycling. The cycling performance of ASSBs at high charge–discharge rates remains challenging owing to the low conductivity of the decomposition products and small contact areas or space-charge layers. de Klerk and Wagemaker [343] proposed a mathematical model to elucidate the space charges of the LATP cathode.

**Figure 31:**
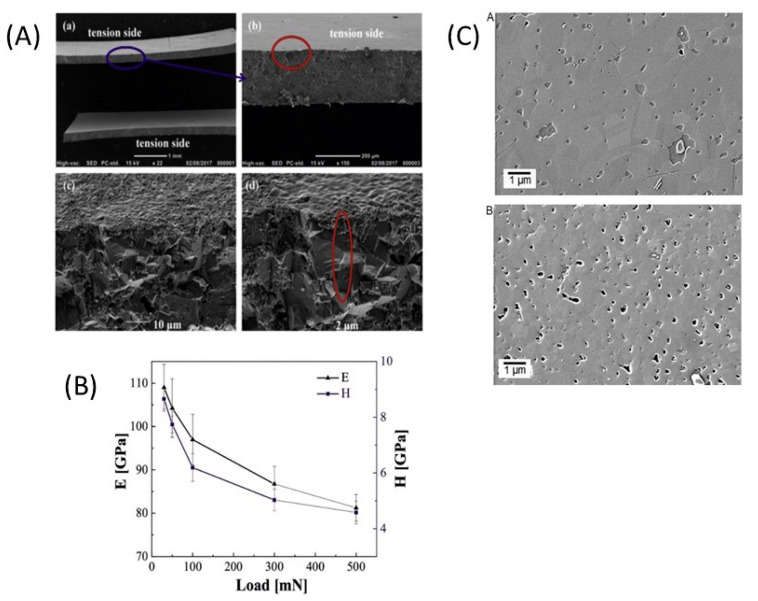
(**A**) Typical crack surface of a Li_1.5_Al_0.5_Ti_1.5_P_3_O_12_:Si (LATP:Si) sample. Images (**a**)–(**d**) illustrate the same sample at different magnifications. The area encircled in blue in (**a**) is magnified in (**b**), where the area encircled in red depicts the potential fracture origin; images (**c**) and (**d**) illustrate the highly magnified fracture surface. The area encircled in red in (**d**) illustrates the transgranular crack growth. (**B**) Elastic modulus and hardness of LATP:Si as functions of the indentation load. Reproduced with permission from [372]. Copyright 2020 Elsevier. (**C**) Microstructure of LATP ceramics fabricated by milling powder after spark plasma sintering at (**a**) 950 and (**b**) 1000 °C. The LATP main phase is interrupted by small amounts of secondary phases and residual porosity. Thereby, the grain growth with increasing temperature and the inclusion of intergranular pores are observed. Reproduced with permission from [378]. Copyright 2020 Elsevier.

Recently, Dashjav et al. [372] reported the microstructure, ionic conductivity, and mechanical properties of the LATP prepared using the tap cast technique. Using this technique, they obtained 99.8% highly dense sheets by adding 1.5% amorphous silica to the slurry; moreover, the elastic modulus and low-load hardness of LATP:Si were 109 ± 5 GPa and 8.7 ± 0.4 GPa, respectively (Figure 31A–C). These properties are important for the fabrication of SSBs. Moreover, the ionic conductivities of LATP and LATP:Si at 20 °C were reported be 0.1 and 0.2 mS cm^−1^, respectively. In addition, the films were sintered at 920 °C and it was concluded that the conductivity of the films increased with the sintering temperature. The microstructure of LATP ceramics fabricated by milling after spark plasma sintering at 950 and 1000 °C is shown in Figure 31C. The LATP main phase is interrupted by small amounts of secondary phases and residual porosity. Thereby, the grain growth with increasing temperature and the inclusion of intergranular pores are observed [378].

Recently, Kou et al. [379] reported the remarkable cycling stability of a spray-drying and assisted sintering-processed ASSB where Li_1.3_Al_0.3_Ti_1.7_(PO_4_)_3_ (LiATP), LCO, and Li metal were the electrolyte, cathode, and anode, respectively. They reported that the capacity of the cell was 150 mA g^−1^ at the rate of 0.1C. Moreover, the cell presented good charge–discharge profiles and cycling performances, similarly to that of liquid electrolyte cell showing the main redox couples (4.0/3.85 V) and hexagonal phase transformations of LiCoO_2_ around ~4.06, ~4.18, ~4.5 V vs. Li [356]. We note that it is not in the experimental part that authors used any liquid or polymer electrolyte to improve the wettability, as they may lead to improved cyclability. Kwatek et al. [380] examined the impact of Li_2.9_B_0.9_S_0.1_O_3.1_ glass additive on the structure and electrical properties of the LATP-based ceramics. Using high-resolution synchrotron-based X-ray and neutron powder diffraction, Monchak et al. [381] characterized the crystal structure of LATP samples prepared by a water-based sol-gel process and evaluated the possible lithium diffusion pathways using the difference bond-valence approach.

Hofmann et al. [382] fabricated LATP and LiCoPO_4_ thin films using the PLD technique and reported various surface analysis methods. Time-of-flight secondary-ion mass spectrometry studies on the as-deposited (unheated) films revealed well defined interfaces; conversely, the interdiffusion of Co and Ti ions was observed between the heat-treated electrolyte and cathode films. Atomic force microscopy analysis revealed that LATP presented well-defined smooth surface and XPS studies indicated that no changes occurred in the oxidation states of the ions at the electrode/electrolyte interface. Recently, Bock et al. [342] reported that the thermal conductivity of LATP was approximately 0.49 ± 0.2 W K^−1^ m^−1^.

Waetzig et al. [378] synthesized LATP using the sol–gel method followed by ball milling and further densification of the powders using the SPS technique. The LATP pellets sintered at 1000 °C presented the excellent room-temperature Li^+^ ion conductivity of 1 × 10^−3^ S cm^−1^, bulk density of 2.92 g cm^−3^, and relative density of 99.4%. In contrast, the Li^+^ ion conductivities of the samples sintered at 800 and 850 °C were 1.1 × 10^−4^ and 4.8 × 10^−4^ S cm^−1^, respectively, and their relative and (bulk) densities were 87.4 % (2.57 g cm^−3^) and 96.1 % (2.824 g cm^−3^), respectively. Although the excellent ionic conductivity of the LATP pellets sintered at 100 °C was ascribed to the samples being homogeneous and crack-free (Figure 31C), the optimum sintering temperature range for the NMC cathodes for ASSBs is 700–800 °C, as in this temperature range, the formation of a reactive phase at the cathode/electrolyte interface is avoided. However, the aforementioned surface morphology is of interest for the fabrication of ASSBs. Pogosova et al. [383] studied the effect of storing the LATP electrolyte in air and Ar atmosphere and reported that the total initial room-temperature conductivity of 4 × 10^−4^ S cm^−1^ decreased significantly, by 76% and 28% for the samples stored in air and Ar, respectively, after three months.

Recently, Case et al. [384] performed computational studies of LATP and Binninger et al. [276] analyzed the electrochemical stability window of the LATP electrolyte using computational methods. Furthermore, Siyal et al. [385] analyzed a gel polymer electrolyte with 15 wt.% LATP composite, and few other researchers studied bare and LATP composite electrodes [379,386,387,388]. Yen et al. [389] characterized LATP powders prepared by hydrothermal synthesis followed by calcination (900–1100 °C), cold pressing (90 MPa), and post sintering, which exhibit ionic conductivity of grain and grain boundary of 6.57 × 10^−4^ and 4.59 × 10^−4^ S cm^−1^, respectively. The fabricated NCM523|LATP|artificial graphite pouch cell delivered a high reversible capacity of 16.7 mAh at 0.5C after 360 cycles with 63.2% capacity retention (voltage range, 2.80–4.25 V).

**Figure 32:**
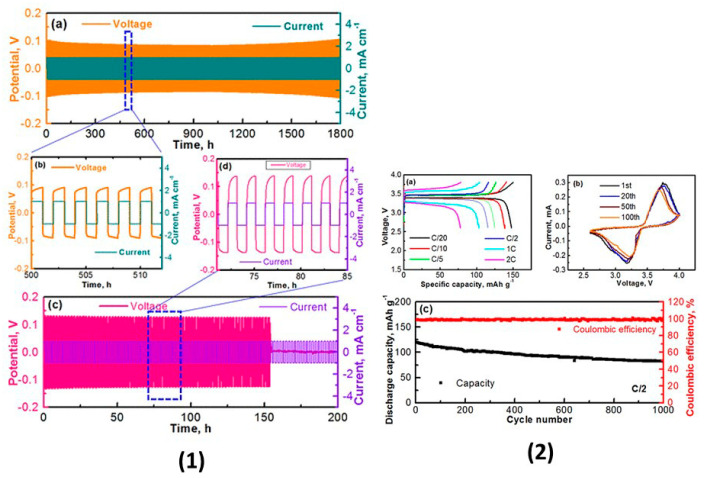
(**1**) Current and voltage profiles of the symmetric Li|PEO-LiCF_3_SO_3_-LATP|Li cell at a current density of ±1.0 mA cm^−2^ and 60 °C(**a**). Here PEO and LATP denote polyethylene oxide and Li_1.5_Al_0.5_Ti_1.5_P_3_O_12_, respectively. (**b**) Magnified profile of marked region of the current and voltage plots in (**a**). (**c**) Current and voltage plots of the symmetric Li|PEO-LiCF_3_SO_3_-LATP|Li cell at 60 °C. The applied current density was ±1.0 mA cm^−2^. (**d**) Magnified profile of the marked region of the current and voltage profiles in (**c**). (**2**) Rate capability of the Li|PEO-LiCF_3_SO_3_-LATP|LFP cell: (**a**) Charge−discharge profiles at various cycling rates at 60 °C, (**b**) cyclic voltammetry curves at different cycles, and (**c**) long-term electrochemical performances of the cell; Coulombic efficiency and discharge capacity vs. cycle number. The cell was operated at a rate of C/2 and 60 °C. Reproduced with permission from [393]. Copyright 2020 American Chemical Society.

Few attempts have been made to combine polymer electrolytes with LATP to obtain solid electrolyte composites. Ma et al. [390] paired a 10% LATP and polymer electrolyte/ionic liquid composite with a LiFePO_4_ cathode and reported a capacity of 138 mAh g^−1^ after 250 cycles with 98% capacity retention at 60 °C. In addition, Wang et al. [391] and Jin et al. [392] studied LATP polymer composites. Yu and Manthiram [393] fabricated a slurry cast PEO–LiCF_3_SO_3_–LATP composite membrane solid electrolyte and paired it with a LiFePO_4_ cathode. Moreover, they studied the effect of various LATP solid electrolyte and polymer compositions and reported that the highest ionic conductivity of 1.6 × 10^−4^ S cm^−1^ at 60 °C was achieved when the amount of LATP electrolyte was 25 wt.%; in addition, when the membrane was paired with a Li metal anode, it was stable for up to 1800 h (Figure 32(1)). The cell formed by combining this composite electrolyte with a LiFePO_4_ cathode and Li metal anode presented the charge capacities of 150 and 118 mAh g^−1^ at the rates of C/20 and C/2 (1C = 2.1 mA cm^−2^), respectively, at 60 °C (Figure 32(2)). These electrolyte systems were difficult operate at room temperature owing to their conductivity limitations. Further improvement in cycling temperature is possible via polymer backbone modifications (Table 4).

DeWees and Wang [49] reviewed various synthesis (see Figure 29) and ionic conductivity analysis methods for the LAGP electrolyte. It was concluded that the processing parameters such as heat-treatment and time and precursor compositions have a great importance in solid-state reaction and sol-gel method, respectively. For example, the use of phosphorous source (H_3_PO_4_) as precursor provides the best LAGP phase purity and the highest ionic conductivity of ~5 × 10^−4^ S cm^−1^ at 25 °C. In addition, few studies on the synthesis, conductivity (~4 × 10^−4^ S cm^−1^, see Table 3 [365]) and interface mechanisms, and physical and electrochemical properties of LAGP have been published since 2019 [49,342,394,395,396,397,398,399,400,401,402,403,404,405,406,407,408,409,410,411,412,413,414,415,416,417,418,419,420,421,422,423,424,425,426,427,428,429,430,431,432,433,434,435,436,437].

Bock et al. [342] reported that the thermal conductivity of LAGP was approximately 0.5 ± 0.2 W K^−1^ m^−1^; moreover, Rohde et al. [398] reported other thermo-physical properties of the (Li_1+*x*_Al*_x_*Ge_2−*x*_)(PO_4_)_3_ solid electrolyte with *x* = 0.3–0.7. Recently, Paolella et al. [438] reported the optimum conditions for densification of Li_1.5_Al_0.5_Ge_1.5_(PO_4_)_3_ at a low temperature of 650°C using hot-press technique (56 MPa applied pressure); this solid electrolyte was used in all-solid-state battery with LiFePO_4_ cathode without addition of any further polymer or liquid electrolyte additives.

**Figure 33:**
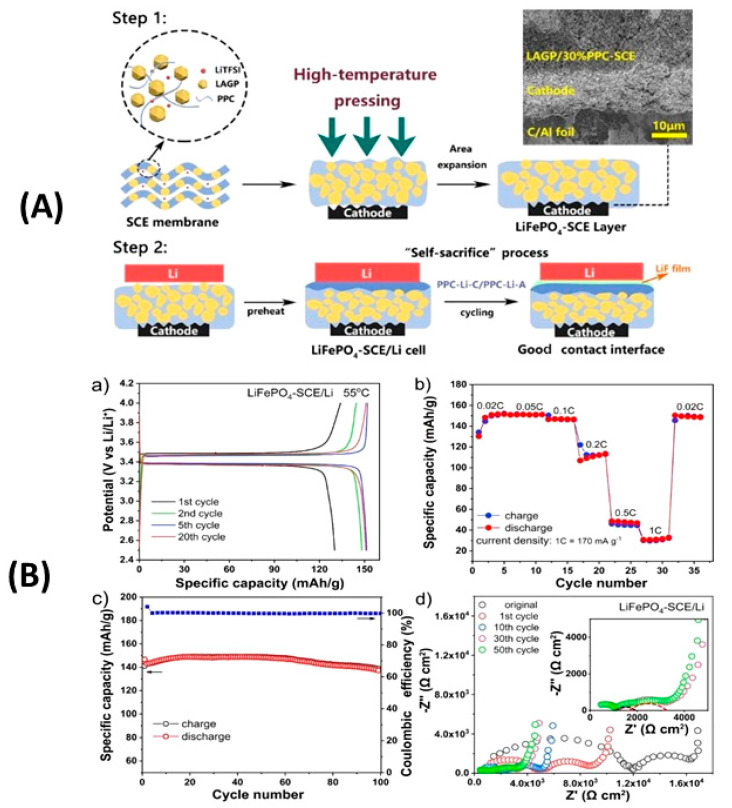
(**A**) Schematics of all-solid-state battery assembly. Step 1 illustrates the hot press progress of the LiFePO_4_ cathode and LAGP/30%-SCE electrolyte (where LAGP and SCE denote Li_1.5_Al_0.5_Ge_1.5_(PO_4_)_3_ and solid composite electrode, respectively) and scanning electron micrograph of the cross-section of the contact interface. Step 2 depicts the preactivation of the LiFePO_4_-SCE||Li cell. (**B**) Voltage profiles of the LiFePO_4_-SCE||Li cell at the current rate of 0.02C (**a**). Rate performance of the LiFePO_4_-SCE/Li cells at current rates in the range of 0.02–1C (**b**). Cycling stability of the LiFePO_4_-SCE/Li cell at the current rate of 0.05 C and 55 °C (**c**). Nyquist plots of the LiFePO_4_-SCE||Li cells before and after different cycles, and magnified areas of the plots in the inset (**d**). Reproduced with permission from [430]. Copyright 2019 American Chemical Society.

In 2019, Wang et al. [430] studied a composite solid electrolyte comprising LAGP embedded with 30% poly(propylene carbonate) (PPC) and compared it with the standard LiTFSI electrolyte using the steps illustrated in Figure 33A. They reported that the conductivity and *E*_a_, of the LAGP–30 wt.% PPC–SCE electrolyte are 5.5 × 10^−4^ S cm^−1^ at 50 °C and 0.506 eV, respectively, and *T*_g_ of 8.11 °C. An SSB was fabricated using LAGP–30 wt.% PPC–SCE, LiFePO_4_, and Li metal as the composite electrolyte, cathode, and anode, respectively. The battery was formatted at 80 °C for 12 h to ensure optimal contact between the electrodes and electrolyte, and then it was subjected to galvanostatic cycling at 55 °C. The cell presented good reversible charge–discharge profiles at ~3.5/3.4 V vs. Li and delivered a capacity of 151 mAh g^−1^ at a current rate of 0.05 C with 92.3% capacity retention (Figure 33B). Electrostatic impedance studies revealed that the electrode/electrolyte interfacial contact improved with cycling, and the overall resistance decreased with increasing cycle number. In 2007, Notten et al. [439] developed the concept of 3-D integrated all-solid-state rechargeable batteries. Pareek et al. [440] conducted a recent study on the conductivity of NASICON-type lithium tin zirconium phosphate (LiSnZr(PO_4_)_3_) with PVDF and LiTFSI polymer-salt matrix. Xie et al. [441,442], Prabhu et al. [443], and Cassel et al. [444] studied bare and Ca-doped LiZr_2_P_3_O_12_ and reported room-temperature conductivities in the range of 10^−4^–10^−6^ S cm^−1^. In addition, Abdel-Hameed et al. [445] investigated the effect of F^−^ and B^3+^ ions and heat treatment on the enhancement of electrochemical and electrical properties of nanosized LiTi_2_(PO_4_)_3_ glass-ceramic for ASSB and Kahlaoui et al. [446] examined the influence of preparation temperature on ionic conductivity of titanium-defective Li_1+4x_Ti_2−x_(PO_4_)_3_ NASICON-type oxide solid electrolytes.

### 4.3. Perovskite-Type Structure Electrolytes

In 1984, Latie et al. [447] reported the synthesis and transport properties of two-dimensional Li*_x_M*_1/3_Nb_1−*x*_Ti*_x_*O_3_ (*M* = La, Nd) perovskite (*AB*O_3_)-type oxides. In addition, they investigated the ion conduction mechanism of these materials using the NMR technique. Furthermore, in 1984, Kochergina et al. [448] published a report on Li_0.5_La_0.5_TiO_3_. Subsequently, the Li_3*x*_La_(2/3)−*x*_□_(1/3)−2*x*_TiO_3_ phase (with 0 < *x* < 0.16) (LLTO), where □ denotes a structural vacancy, and its related compounds, have been thoroughly studied by numerous workers [451,452,453,454,455,456,457,458,459,460,461,462,463,464,465,466,467]. Afterwards, in 1987, Belous et al. [449] studied the effect of the Li content on the structure of Li_3*x*_La_(2/3)−*x*_□_(1/3)−2*x*_TiO_3_ (0.04 ≤ *x ≤* 0.17) and performed conductivity measurements. In 1993, Inaguma et al. [450] studied the Li_0.34_La_0.5_TiO_2.94_ electrolyte. Among all Li_3*x*_La_(2/3)−*x*_□_(1/3)−2*x*_TiO_3_ structures, *x* ≈ 0.1 presented a conductivity of 1 × 10^−3^ S cm^−1^ at 25 °C [458] and an *E*_a_ of 0.40 eV. In 2003, Stramare et al. [459] reviewed the perovskite-type solid electrolytes in detail.

Herein, we summarize the findings of the previous reports and discuss a few recent additional studies as follows.

**Figure 34:**
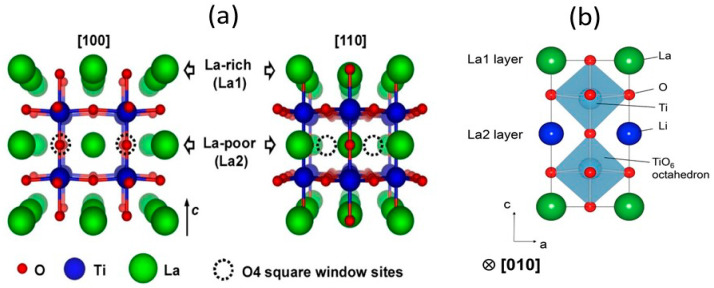
(**a**) Perspective views of the Li_3*x*_La_(2/3)−*x*_□_(1/3)−2*x*_TiO_3_ perovskite network along the [100]_p_ and [110]_p_ zone axes (where “p” refers to the cubic pseudoperovskite structure). Li atoms are not illustrated on account of the uncertainties regarding their positions reported in the literature. Reproduced with permission from [470]. Copyright 2013 American Chemical Society. (**b**) Crystal structure of tetragonal (La_0.5_Li_0.5_)TiO_3_ (*P*4/*mmm* space group). Reproduced with permission from [471]. Copyright 2015 Elsevier.

(i) Many efforts have been invested to elucidate the crystal structure and conduction mechanism of Li_3*x*_La_(2/3)−*x*_□_(1/3)−2*x*_TiO_3_ by (a) analyzing the effect of the preparation method: Solid-state [458], sol–gel [461], precipitation [459], electrospinning [462], and thin film (RF sputtering and PLD) [463], and reaction conditions, such as temperature and time; (b) investigating the concept of doping, i.e., substitution of La by other lanthanides (Pr, Nd, Sm, Gd, Dy, Y) [464], using various Li doping contents [451], or substituting other alkali ions, such as Na^+^ and K^+^ ions, or alkaline-earth ions, such as Sr^2+^ and Ba^2+^ ions, or Ag^+^ ions at the La sites; (c) in 2000, Mizumoto investigated the conductivity relaxation in various lithium ion conductors with the perovskite-type structure [465], and (d) considering doping the Ti sites with tri- (e.g., Al^3+^) [466], tetra- (e.g., Zr^4+^), penta- (e.g., Ta^5+^, Nb^5+^) [485], and hexavalent ions (e.g., W^6+^). It was determined that the conduction mechanism of the LLTO compounds varied with the composition, A-site deficiency, Li^+^ and La^3+^ ions concentration, and dopants [466]. For example, the decrease in *E_a_* and increase in ionic conductivity was noted with increasing the rare-earth metal ion size as follows: Sm^3+^ < Nd^3+^ < Pr^3+^ < La^3+^; furthermore, the microstructure, density, domain size, and composition of the domain boundaries affected the ionic conductivity and *E*_a_ values of the LLTO compounds [467,468,469,470]. Solid-state NMR studies revealed that the Li^+^ ions hopped between cages through the bottleneck in the *ab* plane at low temperature, whereas at high temperature, the Li^+^ ions hopped in all three directions. The reported conductivity values of Li_0.34_La_0.56_TiO_3_ range from ~7 × 10^−4^ to ~1 × 10^−3^ S cm^−1^ (Table 3).

(ii) The Li_3*x*_La_(2/3)−*x*_□_(1/3)−2*x*_(*A*)Ti(*B*)O_3_ perovskite electrolyte presents three different types of polymorphs [459], viz. simple cubic: *a* = 3.872 Å, for *x* = 0.97–0.11, tetragonal: *a* = *b*= 3.87 Å and *c* = 7.74 Å, for *x* = 0.11–0.2, and orthorhombic: *a* = 3.864 Å, *b* = 3.875 Å, *c* = 7.786 Å, for *x* = 0.03–0.09. Among all polymorphs, the cubic structure presented the highest conductivity followed by the tetragonal and orthorhombic ones, for the same bulk composition. The low ionic conductivity of the well-ordered phases was correlated with the uneven ordering of Li, La, and vacancies along the *c*-axis. The Li_3*x*_La_(2/3)−*x*_□_(1/3)−2*x*_(*A*)Ti(*B*)O_3_ LLTO presents perovskite (*AB*O_3_)-type structure, where the *A*-sites consist of La, alkaline (Li^+^, Na^+^, K^+^), or rare earth ions, which are arranged in the corners of a cube and the *B*-sites consist of transition metal (Ti) ions, which are located at the center of the cube; the face-center positions are occupied by O atoms. Typically, the *A*- and *B*-sites present 12- and 6-fold coordination (*B*O_6_), respectively, that share corners with each other (Figure 34a,b) [469,471]. The A-sites contain a large number of defects, and the composition of Li_3*x*_La_(2/3)−*x*_□_(1/3)−2*x*_(*A*)Ti*(B*)O_3_ can be written as La_2/3_TiO_3_, which is intrinsically A-cation deficient, with 1/3 of vacant *A*-sites. The La vacancies are partitioned into alternating La-rich and La-poor layers along one axis to form a partially ordered super lattice structure at room temperature. Depending on the Li content of the materials, they present different symmetries. The Li-poor (0.03 ≤ *x* < 0.1) compositions present orthorhombic symmetry, with high La-site occupancy (≥90%) in the La-rich layer and antiphase tilting of the TiO_6_ octahedra. Conversely, the Li-rich (0.1 ≤ *x* < 0.167) compositions present tetragonal symmetry, and the occupancies of the two types of La layers become less dissimilar as the Li content increases [471].

(iii) The experimental observations were further validated by the results of the computational study performed by Jay et al. [472]. They revealed the non-significant significant ordering of the A-site cations in the layers normal to the *c*-axis and indicated that the Li^+^ ions could also diffuse along *c*-axis. Computational studies offered further insight into the size of the bottleneck and indicated a possible increased using large rare-earth or alkaline-earth metal ions as *A*-site ions; moreover, changing the bond strength between the *B*-site cations and O also affects the conductivity of these electrolytes. In addition, Binninger et al. [276] performed computational studies on the electrochemical stability voltage window of these electrolytes.

(iv) The good room-temperature ionic conductivity values motivated researchers to further elucidate the reactivity of LLTO electrolytes with Li metal anodes and the processes that occur at the electrode/electrolyte interface. According to the early study conducted by Bohnke et al. [473] on the galvanostatic cycling of LLTO, the main redox peak occurred at approximately 1.5 V vs. Li. Owing to this drawback, at operating voltage below 2.8 V, the electrochemical reaction with Li leads reduction of Ti^4+^ to lower oxidation state. These studies revealed that the temperature dependence of the ionic conductivity can be modelized by a Vogel–Tamman–Fulcher (VTF)-type relationship. Klingler et al. [474] analyzed Li*_x_*La_(2−*x*/3)_TiO_3_ (*x* = 0.14, 0.23, 0.32, 0.35) and Pr-, Tb-, Cr-, and Fe-doped compounds with the cycling lower limit of up to 1.1 V vs. Li. Lithium intercalation was noted for all analyzed electrolytes, which led to the formation of the Ti^4+^/Ti^3+^ redox couple, which is a drawback when this electrolyte is used for ASSBs.

**Figure 35:**
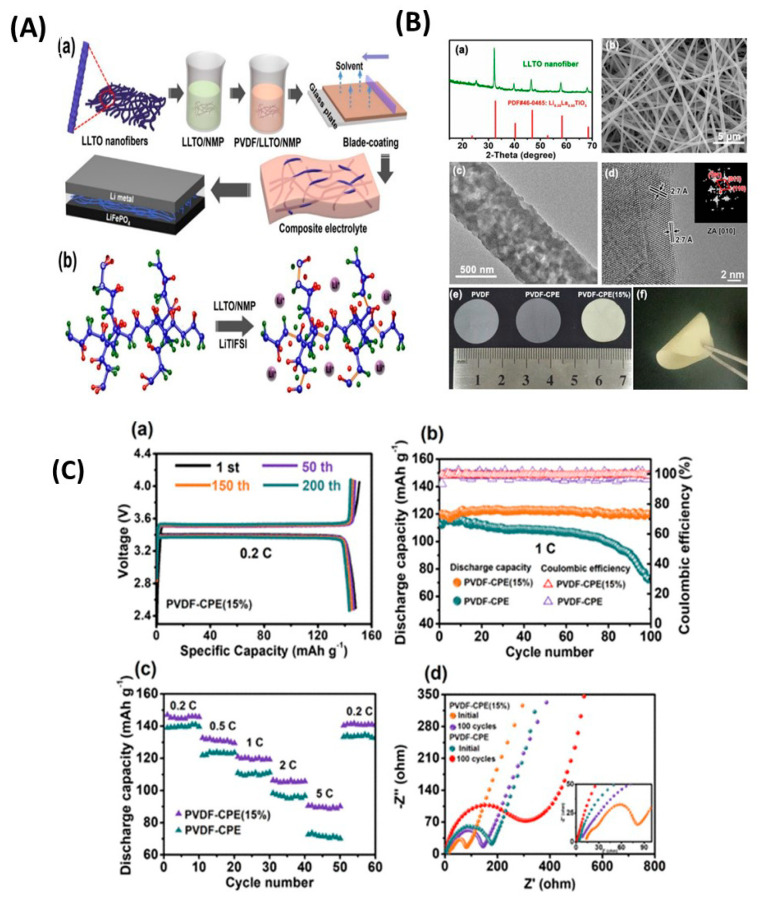
(**A**) Schematic illustration of the preparation procedure of the PVDF–CPEs, and illustration of the electrode configuration for the LMB. Here PVDF, CPE, and LMB denote polyvinylidene fluoride, composite polymer electrolyte, and lithium-metal battery, respectively (**a**). Schematic diagram of changes in PVDF molecular linkages in composite electrolytes(**b**). (**B**) X-ray diffraction pattern (**a**), field-emission scanning electron micrograph (**b**), transmission electron micrograph (**c**), and high-resolution transmission electron micrograph (**d**) of LLTO nanofibers; the inset in (**d**) is the corresponding fast Fourier transform pattern of the LLTO nanofibers. Digital photographs of PVDF, PVDF–CPE, and PVDF–CPE (15%) membranes (**e**). Digital photograph of bent PVDF–CPE (15%) illustrating its good flexibility (**f**). (**C**) Performances of all-solid-state batteries at 25 °C. Charge–discharge curves of the Li|PVDF–CPE (15%)|LiFePO_4_ cell at the current rate of 0.2C (**a**). Long-term cycling (**b**) and rate performances of PVDF–CPE and PVDF–CPE (15%) at the current rate of 1C (**c**). Electrochemical impedance spectroscopy profiles of batteries with PVDF–CPE and PVDF–CPE (15%) electrolytes before cycling and after 100 cycles at the current rate of 0.2C (**d**). Reproduced with permission from [495]. Copyright 2019 American Chemical Society.

Recently, Wenzel et al. [475] studied the LTO/Li metal interface and noted the reduction of Ti^4+^ to Ti^3+,2+, 0^ using XPS analysis. Owing to this drawback, only few reports on the application of the bare LLTO electrolyte for ASSBs have been published. However, for academic purposes, the study conducted by Araki et al. [476] on the fundamental physical properties of Li_3*x*_La_1/3−*x*_*M*O_3_ (*M* = Ta, Nb) revealed that the thermal expansion coefficient was ~3 × 10^−6^ K^−1^ above 400 K regardless of *x*. More studies were conducted to analyze modified synthesis methods, understand the interface mechanisms, and improve the conductivity using modified strategies [276,477,478,479,480,481,482,483,484,485,486,487,488,489,490,491] such as combining 10–15 wt.% LLTO electrolyte with polymer electrolytes/ionic liquid [492] or commercial 1 mol L^−1^ LiPF_6_ in mixture of ethylene carbonate+dimethyl carbonate+diethyl carbonate (EC:DMC:DEC) electrolytes with LLTO, and in some cases using polymer separators. These batteries are typically termed “hybrid composite SSBs”, and the reduction of Ti in the LLTO electrolyte cannot be suppressed in these cells. Lai et al. [493] developed an inter-phase film fabricated by sol-gel electrospinning, which consists of a Li_0.33_La_0.56_TiO_3_ nanofiber (NF) layer deposited on the top of thin lithiophilic Al_2_O_3_ NF layer. This electrolyte was used to form a cell using 1 mol L^−1^ LiPF_6_ (EC:DMC:DEC) and Celgard 2500, LiNi_0.8_Co_0.15_Al_0.05_O_2_, and Li metal as the separator, cathode, and anode, respectively, and the capacity of the cell was 133 mAh g^−1^ at a current rate of 5C in the voltage range of 2.7–4.3 V. Xu et al. [494] observed interdiffusion and amorphous film formation for the Li_0.33_La_0.57_TiO_3_/LiMn_2_O_4_ half-cell. Jiang et al. [486] formed a cell using the LLTO-41/PEO composite, LFP, and Li metal as the electrolyte, cathode, and anode, respectively, and reported that its capacity was 145 mAh g^−1^ with 86.2% capacity retention after 50 cycles; cycling was performed at 65 °C at the current rate of 0.1C. Li et al. [495] fabricated flexible CPE based on LLTO nanofibers embedded in a PVDF matrix with LiTFSI as Li salt and studied the sandwich type LiFePO_4_|PVDF, LiTFSI-CPE (15 wt.% LLTO)|Li cell, in which the 15 wt.% electrospun LLTO fibers (Figure 35A,B) were dispersed with PVDF. The room-temperature conductivity of the LiTFSI electrolyte membrane was 5.3 × 10^–4^ S cm^−1^; moreover, the membrane presented high mechanical strength (stress of 9.5 MPa and strain of 341%), and good thermal stability (thermal degradation at 410 °C). The reversible capacities of the fabricated battery at the current rates of 0.2, 0.5, 1, 2, and 5C were 147, 129, 120, 107, and 91 mAh g^−1^, respectively (Table 4); moreover, good capacity retention was noted at low and high current rates (Figure 35C). Several workers examined the local structure of LLZO; Jin at al. [496] synthesized Al-doped Li_7_La_3_Zr_2_O_12_ synthesized by a polymerized complex method, while Barai et al. [497] investigated the role of the polycrystalline grain/grain-boundary microstructure.

### 4.4. Li Superionic Conductor-Type Structure Oxide Electrolytes

In 1972, West [498] published a report on Li superionic conductor (LISICON)-type structure oxide electrolytes. The conductivities of Li_4_SiO_4_ [310,498,499] and Li_4_Si_0.6_Ti_0.4_O_4_ [310] were reported to be 2 × 10^−9^ and ~3 ×10^−4^ S cm^−1^ at room temperature and 300 °C, respectively. These materials present the γ-Li_3_PO_4_ structure, where Li^+^ ions that are located in the LiO_4_ tetrahedra diffuse between these tetrahedra and interstitial sites in the PO_4_ network. Different solid solutions could replace the P^5+^ ions in γ-Li_3_PO_4_ with tetravalent atoms, such as Si, Ti, and Ge, to create compositions such as Li_3+*x*_(P_1__−*x*_*M_x_*)O_4_.

In 1978, Hong [500] reported LISCON-type structured compounds, such as Li_14_Zn(GeO_4_)_4_ and doped Li_l6.2_*A_x_*(*B*O_4_)_4_, in which *A*^2+^ = Mg, Zn, *B*^4+^ = Si, Ge, and *x* = 1, 2, or 3. Among the analyzed specimens, Li_14_ZnGeO_4_ presented good conductivity (8 S cm^−1^ at 300 °C). Ivanov-Shitz and Kireev [501] reported that the conductivity of single crystal Li_3.34_P_0.66_Ge_0.34_O_4_ was ~1.8 × 10^−6^ and 3.7 × 10^−2^ S cm^−1^ at 40 and 400 °C, respectively.

Deng et al. [502] conducted both experimental and MD computational studies on several LISICON-related compositions, viz. Li_4±*x*_Si_1−*x*_*X_x_*O_4_ (*X* = P, Al, or Ge), Li_4_SiO_4_, Li_3.75_Si_0.75_P_0.25_O_4_, Li_4.25_Si_0.75_Al_0.25_O_4_, Li_4_Al_0.33_Si_0.33_P_0.33_O_4_, and Li_4_Al_1/3_Si_1/6_Ge_1/6_P_1/3_O_4_. They observed that the conductivities of the P-, Al-, and Ge- doped samples were higher than those of the other samples. In addition, the MD simulation studies revealed that the conductivity of Li_4_Al_1/3_Si_1/6_Ge_1/6_P_1/3_O_4_ was 0.9 mS cm^−1^; furthermore, its *E*_a_ of 0.28 eV was the lowest of all analyzed samples. Recently, Zhao et al. [503] studied the co-doped Li_3.75±*y*_(Ge_0.75_P_0.25_)_1−*x*_*M_x_*O_4_ (*M* = Mg^2+^, B^3+^, Al^3+^, Ga^3+^, and V^5+^) LISICON-type structures and reported that Li_3.53_(Ge_0.75_P_0.25_)_0.7_V_0.3_O_4_ presented the highest ionic conductivity of 5.1 × 10^−5^ S cm^−1^ at 25 °C of all samples, and also the low *E*_a_ of 0.43 eV (Table 3). 

The low room-temperature conductivity of bare oxide electrolytes is a drawback, and hence, very few studies have focused on their use for AASBs. However, some bare oxide electrolytes can be used for high-temperature applications, and according to some recent studies, once the interactions at the cathode/electrolyte interface are elucidated, a few compositions could be promising SSB electrolyte materials.

### 4.5. Amorphous Thin Film Electrolytes

Commercial Li-ion batteries for mobile applications use bulk electrode materials. Conversely, thin-film microbatteries have been explored for miniaturized device applications, such as smart cards, microwave microelectromechanical systems, and other biomedical applications. The electrode and electrolytes of microbatteries are a few microns thick and are deposited layer-by-layer using RF-sputtering, PLD, evaporation, and other techniques. These batteries can only be used for low-power applications owing to their thin film nature; in addition, the deposition technique used for fabricating these devices is expensive compared with the traditional slurry coating method used to manufacture Li batteries. Despite these limitations, after Oudenhoven et al. [117] proposed the concept and design of 3D microbatteries, the use of thin-film electrolytes for microelectronic applications has been explored by many researchers [504,505,506,507,508,509,510,511,512,513,514,515,516,517,518,519,520,521].

Lithium phosphorous-oxynitride (LiPON) is one of the most studied oxide-based electrolytes owing to its reasonably good ionic conductivity and stability when paired with Li metal anode Bates et al. [522] reported that the conductivity of the thin-film Li_3.3_PO_3.9_N_0.17_ electrolyte prepared via RF sputtering using an LPO target and N_2_ reactive gas, was 2 × 10^−6^ S cm^−1^ at 25 °C. Yu et al. [523] further explored LiPON electrolytes and determined that the conductivity, *E_a_*, electrochemical stability window, and bandgaps of Li_2.88_PO_3.73_N_0.14_ were 3.3 × 10^−6^ S cm^−1^ at 25 °C, 0.54 eV, 0–5.5 V, and 3.45 and 3.75 eV, respectively (Table 3). Hamon et al. [524] and Fleutot et al. [525,526,527] reported the effect of the RF-sputtering parameters, such as power, flow rate, and total pressure, under pure N_2_ gas atmosphere on the composition and conductivity properties of Li*_x_*PO*_y_*N*_z_* (*z* = 0.4–1.2) LiPON thin films, and noted that the ionic conductivity increased with the incorporation of N_2_ into the glassy structure. The correlations between composition, local structure (by XPS), and the electrical properties were reported for lithium borophosphate (Li_3_PO_4_B*_x_*, *x* = 0.08–0.24) thin films and for *x*LiBO_2_:(1−*x*)Li_3_PO_4_ (*x* = 5, 10, 15, 20, 25) glasses [527]. The effect of the B/P ratio on the conductivity of the electrolytes was analyzed demonstrating that the electrolyte with the B/P ratio of 0.1 presented the highest ionic conductivity of 1.1 × 10^−6^ S cm^−1^ and lowest *E*_a_ of 0.52 eV of all analyzed samples.

**Figure 36:**
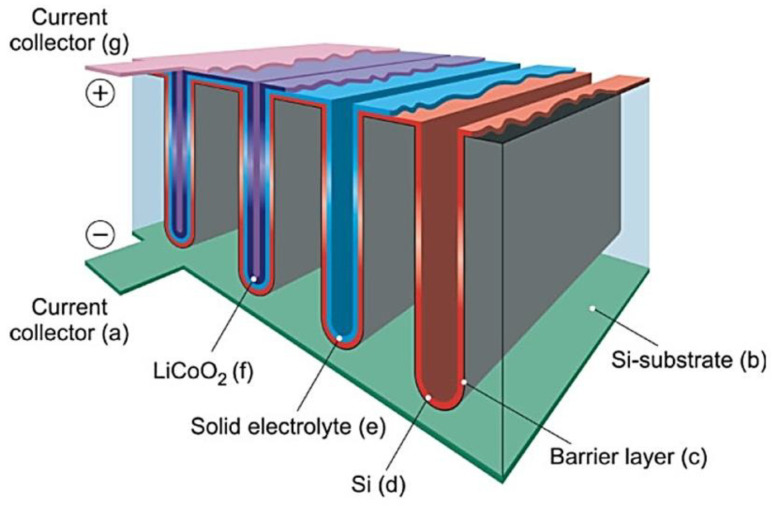
Schematic diagram of three-dimensional microbattery with lithium phosphorous oxynitride electrolyte. Reproduced with permission from [439]. Copyright 2007 Wiley.

Joo et al. [528] studied (1−*x*)LiBO_2_–*x*Li_2_SO_4_ (LiBSO) (*x* = 0.4–0.8) amorphous solid electrolytes thin films and reported that the ionic conductivity of the electrolyte increased with *x* and was the highest (~2.5 × 10^−6^ S cm^−1^) when *x* = 0.7 at room temperature. In addition, they noted that at *x* > 0.7 the conductivity values slowly decreased owing to the partial crystallization of the electrolytes. Furthermore, Schwenzel et al. [529] studied the LiAl|LiPON|LiCoO_2_ thin film battery and Notten et al. [439] fabricated 3D microbatteries, in which LiPON and LCO were used as the electrolyte and cathode, respectively (Figure 36). Recently, Famprikis et al. [530] reported that the maximum ionic conductivity and *E*_a_ of the Li_3+*x*_Si*_x_*P_1−*x*_O_4_ (LiSiPON) thin films obtained via RF sputtering under Ar and N_2_ atmospheres were 2.06 × 10^−5^ S cm^−1^ and 0.45 eV, respectively, and these values were one order of magnitude higher than those of LiPON thin films. Furthermore, Clancy and Rohan [531] conducted modelling studies of thin-film batteries and electrolytes.

### 4.6. Other Electrolytes

In 1981, Hellstrom and Van Gool [532] reported that the Li^+^ ion conductivity values of Li_2_ZrO_3_, Li_4_ZrO_4_ and LiScO_2_ were 3.3 × 10^−5^, 3.0 × 10^−4^, and 4.2 × 10^−7^ S m^−1^, respectively, at 300 °C. Although these materials presented low room-temperature conductivity, their chemical stability when paired with Li metal anodes was good. Furthermore, few studies focused on Zr-based fast ion conductors, such as bare and Ta-, Nb-, Y-, and In-doped Li_6_Zr_2_O_7_ [533,534,535]. The ionic conductivity of Ta-doped Li_6_Zr_2_O_7_ oxide was reported to be 1 × 10^−3^ S cm^−1^ at 300 °C [536].

## 5. Conclusions

In this review article, we summarized the recent advances and challenges of ASSBs with sulfides and oxide electrolyte systems. Owing to their excellent ionic conductivities, Li_3_PS_4_ and LiPS_5_Cl have been the most studied sulfide electrolytes. The AASBs formed when these electrolytes were paired with Ni-rich NMC cathodes achieved high energy densities. Although the room-temperature ionic conductivity of sulfide electrodes is good and these electrolytes can be easily fabricated, their stability should be further improved to expand their large-scale applications. To fabricate batteries with good electrochemical performance, the sulfide electrolyte should be paired with cathodes that are coated with protective layers of LiNbO_3_, Li_3_PO_4_, Li_2_ZrO_3_ or other metal oxides. Moreover, the surface protection of the cathodes involves additional costs, and therefore, a cost-effective novel approach for the large-scale manufacturing of ASSBs is needed. Sulfide electrolytes present a few other shortcomings, including short cycle life, low stability, narrow electrochemical voltage window, suboptimal electrode/electrolyte interface, and low stability in air.

Among all oxide-based electrolytes, garnet-based oxides, Ta-, Ga-, and Al-doped Li_7_La_3_Zr_2_O_12_, and NASICON-type LATP and LAGP have been studied in depth for ASSBs, owing to their good conductivity. Only few studies have been conducted on ASSBs with Ta-doped LLZO electrolytes, owing to their better stability when paired with Li metal anodes. Most oxide-based electrolytes use 15–25 wt.% inorganic superionic conductors (LATP, LAGP, LLTO) in polymer composites with combination of ionic liquid electrolytes. However, the progress in this field has been rather slow, mainly owing to the high cell resistance, which was attributed to the high-temperature sintering process required for better particle-to-particle contact between composite cathodes and electrolyte layers. Most of the best-reported garnet-based electrolyte used high content of Ta dopant (0.5–0.6 mol%) for large-scale application, which can be further reduced below 0.25 mol%. LATP, LAGP, and LLTO contain Ti and Ge, which undergo electrochemical reactions with Li metal, and thus, further improving the surface protection of the electrolytes is needed for large-scale applications and to reduce the cost associated with the use of Ge.

The most common shortcoming of ASSBs with sulfide and oxide electrolyte is their low electrochemical cycling performance at high charge–discharge rates, which is attributed to the poorly conducting decomposition products and small contact areas or space-charge between electrode and electrolyte layers. In addition, the roles of the microstructure adhesion and mechanical and surface interfacial properties of both Li metal and solid electrolytes should be further elucidated. Furthermore, the reactivities of Li metal, solid electrolytes, and cathodes should be further investigated. Currently, it is difficult to access the electrolyte/electrode interface using conventional post-mortem techniques without creating artifacts, and thus, further advances should be made on developing in situ analysis techniques. Moreover, the search for highly stable conductive electrolytes should continue. Lastly, an important aspect related to the fabrication of ASBB would be the cooperation between scientists and engineers, which could facilitate the fabrication of large-area cells and address the current transportation technology challenges.

## Figures and Tables

**Table 1:** Room temperature ionic conductivity σ_(RT)_ and activation energy *E*_a_ of sulphide solid electrolytes.

Electrolyte	Structure, lattice Parameters (Å)	σ_(RT)_ (S cm^−1^)	*E*_a_ (eV)	Ref.
Li_6_PS_5_Cl	amorphous crystalline cubic, a = 9.85	3.3 × 10^−5^ 1.9 × 10^−9^	0.38 0.35 ^a^	[181]
Li_6_PS_5_Br	amorphous crystalline, a = 9.98	3.2 × 10^−5^ 6.8 × 10^−3^	0.32 (0.32) ^a^	[181]
Li_6_PS_5_I	amorphous crystalline, a = 10.142	2.2 × 10^−4^ 4.6 × 10^−7^	0.26 0.25 ^a^	[181]
β-Li_3_PS_4_	amorphous	2.8 × 10^−4^	0.37	[229]
Li_3.25_Si_0.25_P_0.75_S_4_	crystalline, orthorhombic a = 13.158, b = 8.029, c = 6.129	1.22 × 10^−3^	0.20	[222]
Li_7_P_3_S_11_	crystalline, triclinic a=12.501, b= 6.031, c=12.530	0.1–0.2 × 10^−3^	0.2–0.4	[244]
Li_7_P_2_S_8_I	crystalline, orthorhombic	6.3 ×10^−3^	0.31	[261]
Li_7_P_2_S_8_I	crystalline, orthorhombic *a* = 12.703, *b* = 8.45, *c* = 5.94	6.07×10^−3^	0.27	[184]
Li_15_(PS_4_)_4_Cl_3_ Li_14.8_Mg_0.1_ (PS_4_)_4_Cl_3_	crystalline, *a* = 14.308 *a* = 14.323	4.0 × 10^−8^ 2.0 × 10^−7^	0.59 0.41	[188]
Li_10_GeP_2_S_12_	crystalline, tetragonal *a* = 8.717; *c* = 12.634	12 × 10^−3^	0.24	[266]
Li_10_GeP_2_S_12_	tetragonal *a* = 8.718, *c* = 12.660	9.0 × 10^−3^	0.22	[269]
Li_10_GeP_2_S_12_	crystalline, tetragonal *a* = 8.712, *c* = 12.617	10 × 10^−3^	0.30	[292]
Li_10_SiP_2_S_12_	crystalline, tetragonal *a* = 8.658, *c* = 12.519	2.0 × 10^−3^	0.30	[292]
Li_10_SiP_2_S_11.3_O_0.7_	crystalline, tetragonal *a* = 8.666, *c* = 12.529	3.1 × 10^−3^	0.32	[290]
Li_10_SnP_2_S_12_	crystalline, tetragonal *a* = 8.734, *c* = 12.773 Å	6.0 × 10^−3^	0.31	[292]
Li_10_Si_0.3_Sn_0.7_P_2_S_12_	crystalline, tetragonal *a* = 8.741, *c* = 12.757	8.0 × 10^−3^	0.29	[292]
Li_10.3_Al_0.3_Sn_0.7_P_2_S_12_	crystalline, tetragonal *a* = 8.743, *c* = 12.787	5.0 × 10^−3^	0.29	[292]
Li_9.42_Si_1.02_P_2.1_S_9.96_O_2.04_	tetragonal	1.1 × 10^−4^	0.23	[296]
Li_9.54_Si_1.74_P_1.44_S_11.7_Cl_0.3_	crystalline, tetragonal *a* = 8.709, *c* = 12.569	2.53 × 10^−2^	0.23	[296]
Li_11_AlP_2_S_12_	crystalline	8.02 × 10^−4^	0.25	[302]
β-Li_3_PS_4_	amorphous	2.0 × 10^−4^	0.34	[303]
β-Li_3_PS_4_	crystalline, orthorhombic *a* = 13.066, *b* = 8.015, c = 6.101 amorphous	1.6 × 10^−4^ 7.4 × 10^−5^	0.36	[304]

^a^ calculated by bond valence approach.

**Table 2:** Electrochemical performance of sulfide-based electrolytes for all-solid-state batteries.

Electrode Fabrication	Electrochemical Studies.	Specific Capacity Rate Capability Capacity Retention	Ref.
(Li−In|β-Li_3_PS_4_|NMC-811/β-LPS) s_RT_ (β-Li_3_PS_4_) = 3.2 × 10^−3^ S cm^−1^ Composite cathode/electrolyte ratio of 70:30 w/w. Powders pressed at 445 MPa	Voltage range 2.7−4.3 V vs. Li^+^/Li at 25 °C Pressure during electrochemical measurements was maintained at 70 MPa (areal loading of 10.7 mg cm^−2^)	Specific capacity of 125 mAh g^−1^ at 0.1C rate	[203]
Carbon-coated Li_4_Ti_5_O_12_ (LTO), β-LPS, and Super C65 carbon black (3:6:1) (30 mg, ~120 µm thick, pressed at 125 MPa)|β-LPS (60 mg, ~500 µm thick, pressed at 125 MPa)|Li_2_CO_3_, Li_2_CO_3_-LiNbO_3_–coated NMC622 (10–12 mg, ~90 µm thick, pressed at 375 MPa)	Voltage range 1.35–2.85 V vs. LTO (equivalent to 2.9−4.4 V vs. Li^+^/Li) at 25 °C. Pressure during electrochemical measurements was maintained at 55 MPa	Bare NMC capacity of 136 and 106 mAh g^−1^; rate of C/10; capacity retention of 64%. Li_2_CO_3_-coated NMC; capacity of 148 and 124 mAh g^−1^; capacity retention of 79% Li_2_CO_3_-LiNbO_3_–coated NMC; capacity of 157 and 136 mAh g^−1^; capacity retention of 91%	[204]
Li_0.5_In/Li_6_PS_5_Cl/|LiNi_0.8_Co_0.15_Al_0.05_O_2_ 2 wt.% coated LiNbO_3_	Voltage range 2.5–4.3 V Stack pressure during cycling of 5 MPa	150 mAh g^−1^ after 5 cycles at 0.1C rate Capacity retention of 80.9% over 100 cycles	[201]
Li0.5In/Li_7_P_2_S_8_I/|LiNi_0.6_Co_0.2_Mn_0.2_O_2_ 3 wt.% coated LiNbO_3_, Li_2_O–ZrO_2_	Voltage range 2.38–3.68 V, coin, no pressure applied during cycling	Specific capacity135 mAh g^−1^ Current rate of 0.1C (18 mA g^−1^)	[265]
LiIn/LPS/NMC111:SE(75:25) Composite electrode pressed at 360 MPa, Li/In foil pressed at 240 MPa	Voltage range 1.9–3.8 V Stack pressure during cycling of 25 MPa	Reversible capacity of 100 mAh g^−1^ and ~80 mAh g^−1^ after 50 cycles Current rate of 0.13 mA cm^−2^	[209]
In|90Li_7_P_3_S_11_–10Li_2_OHBr |Li(Ni_0.6_Co_0.2_Mn_0.2_)O_2_ (70:28:2) (Li(Ni_x_Mn_y_Co_z_)O_2_:electrolyte carbon)	Voltage range 2.38–3.62 V vs. In	Reversible capacity of 135 mAh g^−1^ Current density of 0.05 C (7.5 mA g^−1^)	[210]
Li/LGPS/Li_10_GeP_2_S_12_ hierarchical coverage Li_3_PO_4_-coated NMC811:LGPS (70:30) Composite electrode pressed at ~380 MPa	Voltage range 2.7–4.5 V vs. Li	Reversible capacity of ~133 mAh g^−1^ at 0.1C rate after 100 cycles (~96 mAh g^−1^ after 300 cycles)	[280]
Li-In/Li_9.34_Si_1.74_P_1.44_S_11.7_Cl_0_._3_/LNO@NMC811 composite electrode pressed at 300 MPa Li/In foil pressed at 280 MPa	Voltage range 2.1–3.8 V vs. Li	Reversible capacity of 197 mAh g^−1^ at 0.3C rate, 83% capacity retention after 500 cycles	[298]

**Table 3:** Room temperature ionic conductivity σ_(RT)_ and activation energy *E*_a_ of oxide solid electrolytes.

Electrolyte	Structure, Lattice Parameter (Å)	σ_(RT)_ (S cm^−1^)	*E*_a_ (eV)	Ref.
Li_7_La_3_Zr_2_O_12_	garnet type, cubic *a* = 12.82–13.01	10^−3^–10^−4^	0.31–0.34	[73]
Li_7_La_3_Zr_2_O_12_	crystalline, tetragonal *a* = 13.068, *c* = 12.66	10^-5^ -10^-6^	0.40-67	[317]
Li_6.75_La_3_Zr_1.75_Ta0.25O_12_	crystalline, cubic *a* = 12.96	0.87 × 10^−3^	0.22	[317]
Li_6.5_La_3_Zr_1.5_Ta0.5O_12_	crystalline, tetragonal *a* = 12.929	0.75 × 10^−3^	-	[355]
Li_6.15_La_3_Zr_1.75_Ta_0.25_Al_0.2_O_12_	crystalline, cubic, *a* = 12.95	0.37 × 10^−3^	0.30	[317]
Li_6.25_La_3_Zr_2_Al_0.25_O_12_	crystalline, cubic, *a* = 12.96	0.68 × 10^−3^	-	[355]
Li_6.15_La_3_Zr_1.75_Ta_0.25_Ga_0.2_O_12_	crystalline, cubic, *a* = 12.95	0.41 × 10^−3^	0.27	[317]
Li_6.25_La_3_Zr_2_Ta_0.25_Ga_0.2_O_12_	crystalline, cubic *a*= 12.97	1.04 × 10^−3^	-	[355]
Li_1.5_Al_0.5_Ti_1.5_P_3_O_12_	crystalline, hexagonal *a* = 8.50, *c* = 20.52	3.0 × 10^−3^	0.26	[377]
Li_1.5_Al_0.5_Ge_1.5_P_3_O_12_	crystalline, hexagonal *a* = 8.25, *c* = 20.65	4.0 × 10^−4^	0.35	[365]
Li_3x_La_(2/3)–x_□_(1/3)_–2xTiO_3_ (*x* = 0.1)	crystalline, cubic, *a* = 3.872	1.0 × 10^−3^	0.40	[458]
Li_0.34_La_0.56_TiO_3_	crystalline, cubic, *a* = 3.872	1.53 × 10^−3^	0.33	[466]
Li_0.34_La_0.56_TiO_3_	crystalline, tetragonal *a* = 3.87, *c* = 7.74	6.88 × 10^−4^	0.35	[466]
Li_4_Al_1/3_Si_1/6_Ge_1/6_P_1/3_O_4_	LISiCON type structure	0.9× 10^−3^	0.28	[502]
Li_3.53_(Ge_0.75_P_0.25_)_0.7_V_0.3_O_4_	LISICON-type	5.1 × 10^−5^	0.43	[503]
Li_2.88_PO_3.73_N_0.14_ (LIPON)	amorphous	3.3 × 10^−6^	0.54	[523]
Li_3+*x*_Si*_x_*P_1−*x*_O_4_ (LiSiPON)	amorphous	2.06 × 10^−5^	0.45	[530]

**Table 4:** Electrochemical performance of oxide solid electrolytes for all-solid-state batteries.

Electrode Fabrication	Electrochemical Studies	Reversible Capacity Current Rate Coulombic Efficiency	Ref.
LCO/Ta-LLZO|Ta-LLZO|Li-In Ta-LLZO is Li_6.6_La_3_Zr_1.6_Ta_0.4_O_12_ Composite cathode/electrolyte 1:1 w: w Volume ratio of 51.4:48.6 - ASSB thickness of ~50 µm - Electrolyte thickness of 300 µm	Voltage range 2.4−3.65 V vs. Li-In at 50 °C Tested using Swagelok cells No pressure was applied during the electrochemical measurements Composite mass loading of active material of 32 mg cm^−2^ gives 16 mA cm^−2^	Charge and discharge capacity of 1.48 mA cm^−2^ (117 mAh g^−1^) Current density of 50 µA cm^−2^ Coulombic efficiency of 81.5%	[13]
Li/LCO@Li_2_CO_3_ + Li_2.3_C_0.7_B_0.3_O_3_ +LLZO@Li_2_CO_3_ Li_6.4_La_3_Zr_1.4_Ta_0.6_O_12_ (LLZO) weight and corresponding volume ratios of 58:30:12 and 45:30:25	Mass of active material of 1–3 mg cm^−2^ Cathode layer thickness of 20 µm Tested using Swagelok cells - Voltage range 3.0–4.05 V Initially cells were placed in an oven at 100 °C to ensure good contact between the electrodes and electrolyte	Specific capacity of 94 mAh g^−1^ at the rate of 0.05 C at 25 °C. Capacity of 106 mAh g^−1^ at the rate of 0.05C at 100 °C. (1C = 115 mA g^−1^)	[357]
NMC + 5 wt.% LATP glass ceramic on LLZO pellet - cathode: NMC111	Voltage range 3.0–4.2 V at 100 °C Pressure applied during electrochemical cycling of 150 kPa	Specific capacity of 150 mAh g^−1^ Current rate of 50 μA cm^-2^	[358]
Li/ PEO–LiCF_3_SO_3_ LATP ((Li_1.5_Al_0.5_Ti_1.5_(PO_4_)_3_) electrolyte was 25 wt%/LiFePO_4_	Voltage range 2.5–3.8 V at 60 °C	Reversible capacities of 150 and 118 mAh g^−1^ at C/20 (42 µA cm^-2^) and C/2 (0.42 mA cm^-2^), respectively	[393]
Li/PPC (Poly-propylene carbonate)-SCE 30 wt.% LAGP (Li_1.5_Al_0.5_Ge_1.5_(PO_4_)_3_)–30 wt.%/LiFePO_4_	Voltage range 2.5–4.0 V at 55 °C	Capacity of 151 mAh g^−1^ at 0.05C 92.3% capacity retention at 100 cycles	[430]
Li/PVDF, LITSF-CPE (composite polymer electrolyte) (15 wt.% LLTO)/LiFePO_4_	Voltage range 2.5–4.0 V at 25 °C	Reversible capacities of 147, 129, 120, 107, and 91 mAh g^−1^, at 0.2, 0.5, 1, 2, and 5C rates	[495]
